# Transcriptome Sequencing Data Reveal LncRNA-miRNA-mRNA Regulatory Network in Calcified Aortic Valve Disease

**DOI:** 10.3389/fcvm.2022.886995

**Published:** 2022-05-26

**Authors:** Kai Huang, Lujia Wu, Yuan Gao, Qin Li, Hao Wu, Xiaohong Liu, Lin Han

**Affiliations:** Department of Cardiovascular Surgery, Changhai Hospital, Second Military Medical University, Shanghai, China

**Keywords:** bioinformatics analysis, calcified aortic valve disease (CAVD), LncRNA – long noncoding RNA, ceRNA, immune cells

## Abstract

**Background:**

Calcified aortic valve disease (CAVD) is one of the most common valvular heart diseases in the elderly population. However, no effective medical treatments have been found to interfere with the progression of CAVD, and specific molecular mechanisms of CAVD remain unclear.

**Materials and Methods:**

Transcriptome sequencing data of GSE55492 and GSE148219 were downloaded from the European Nucleotide Archive, and the microarray dataset, GSE12644 was acquired from the Gene Expression Omnibus database. Software, including FastQC, HISAT2, samtools, and featureCounts was applied to generate the read count matrix. The “Limma” package in R was utilized to analyze differentially expressed genes (DEGs). Thereafter, weighted gene co-expression network analysis, Gene Ontology (GO) and Kyoto Encyclopedia of Genes and Genomes (KEGG) enrichment analysis, and the protein-protein interaction (PPI) network were used to identify hub genes associated with CAVD, which were further validated by receiver operating characteristic curve (ROC) analysis using GSE12644. The long non-coding RNA (LncRNA)-mediated regulatory network was established based on the differentially expressed LncRNAs and hub genes, which were detected using quantitative real-time PCR (qRT-PCR) in clinical samples and valve interstitial cells. Moreover, CIBERSORT was used to calculate the expression distribution of immune cell infiltration in CAVD.

**Results:**

A total of 126 DEGs were included in the PPI network. PI3K-Akt signaling pathway, ECM-receptor interaction, hematopoietic cell lineage, cell adhesion molecules, and focal adhesion were the most enriched pathways revealed by KEGG. Four LncRNAs, including TRHDE-AS1, LINC00092, LINC01094, and LINC00702 were considered the differentially expressed LncRNA. SPP1, TREM1, GPM6A, CCL19, CR1, NCAM1, CNTN1, TLR8, SDC1, and COL6A6 were the 10 hub genes identified to be associated with CAVD. Moreover, the calcified aortic valve samples had a greater level of Tregs, naïve B cells, and M0 macrophages than the noncalcified ones, whereas CAVD samples had a lower M2 macrophage expression compared to the noncalcified valve tissues.

**Conclusion:**

The current study identified SPP1, TREM1, TLR8, SDC1, GPM6A, and CNTN1 as hub genes that could potentially be associated with CAVD. The LINC00702–miR-181b-5p–SPP1 axis might participate in the development of CAVD. Additionally, M2 macrophages, Tregs, naïve B cells, and M0 macrophages might possibly play a role in the initiation of CAVD.

## Introduction

Calcified aortic valve disease (CAVD), which is mainly caused by aortic valve degenerative stenosis, has gained popularity owing to the aging population (1). Although surgical treatment or transcatheter aortic valve implantation can alleviate the symptoms and improve prognosis, the potential cellular and molecular mechanisms of CAVD still need further exploration to develop new therapeutic strategies.

Accumulating evidence has revealed the role of long non-coding RNAs (LncRNAs) in various cardiovascular diseases, such as myocardial infarction (2), hypertension (3), aortic aneurysm (4), and atherosclerosis (5). Studies have found that LncRNA TUG1 promotes the phenotype switch in aortic valve interstitial cells through miR-204-5p/RUNX2 pathway (6). LncRNA MALAT1 could also sponge miR-204 to promote aortic valve calcification by upregulating SMAD4 (7). These findings highlight the important roles of LncRNAs in CAVD. Nonetheless, only a few studies have focused on the big picture of the LncRNA-associated regulatory network in CAVD.

The current study filtered out statistically significant LncRNAs and mRNAs between calcified aortic valves and noncalcified ones. Thereafter, a protein-protein interaction (PPI) network was established according to these mRNAs. CAVD-associated hub genes were further determined through sub-network and receiver operating characteristic curve (ROC) analysis. Based on differentially expressed LncRNAs, hub genes, and bioinformatics prediction, miRNA-mRNA and LncRNA-miRNA pairs were filtered out and the LncRNA-miRNA-mRNA regulatory network was established. Hub genes and specific LncRNAs in this network were verified through quantitative real-time PCR (qRT-PCR). In addition, immune cell infiltration in CAVD was also executed by using the CIBERSORT tool. This study has laid a solid foundation for further experimental verification and provided new insights into the mechanisms of CAVD.

## Materials and Methods

**FIGURE 1:**
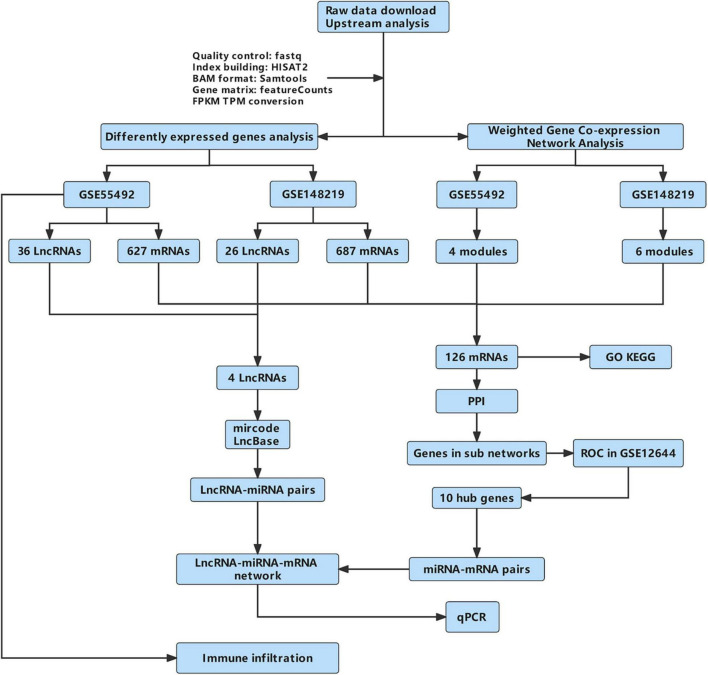
The flow diagram of this work. GO, Gene Ontology; KEGG, Kyoto Encyclopedia of Genes and Genomes; PPI, protein-protein interaction; qPCR, quantitative real-time reverse transcription-polymerase chain reaction.

The flow diagram of this work is shown in Figure 1.

### Data Acquisition and Processing

Transcriptome sequencing data for GSE55492 and GSE148219 were downloaded from the European Nucleotide Archive.^1^ The mRNA microarray dataset GSE12644 was downloaded from the GEO database.^2^ The dataset GSE55492 was carried out on GPL11154 (Illumina HiSeq 2000), including 10 noncalcified aortic valve samples and nine calcified tricuspid aortic valves. Series GSE148219 was performed on GPL16791 (Illumina HiSeq 2500) and contained six noncalcified aortic valves and seven calcified tricuspid aortic valves. Dataset GSE12644 was performed on GPL570, which included 10 normal and 10 aortic stenosis valves. Supplementary Table 1 shows the demographic information of patients in these three GEO datasets.

The quality control of these two transcriptome datasets was controlled by fastqc (version 0.11.9). The reference genome, Homo sapiens GRCh38.104, was downloaded from Ensembl.^3^ The index for the reference genome was built from HISAT2 (version 2.2.1). Thereafter, sequenced reads were aligned to the reference genomes using HISAT2 (version 2.2.1). Samtools (version 1.13) was used to convert the sequence alignment map (SAM) format derived from aligned RNA-Seq samples into binary alignment map (BAM) format. Finally, the read counts matrix for each gene was generated using featureCounts (version 2.0.1). The fragments per kilobase million (FPKM) and transcripts per kilobase million (TPM) matrices were also calculated according to a previous study (8). The read counts data were uploaded into R (software version 4.0.5), after which the differentially expressed gene (DEG) analysis was performed using the voom function in the Limma package (version 3.48.3). The threshold for DEGs was *P*-value < 0.05 and logFC > mean [abs (logFC) + 2 * SD (logFC)]. According to the gene types in the reference genome, differentially expressed LncRNAs and mRNAs were filtered for further analysis.

The heat map, volcano plot, and principal component analysis (PCA) picture were plotted using pheatmap (version 1.0.12), ggplot2 (version 3.3.5), and FactoMineR (version 2.4) in R, respectively.

### Weighted Gene Co-expression Network Analysis

Based on the TPM matrix data of GSE55492 and GSE148219, a step-by-step network construction function was applied to establish the mRNA co-expression network and to filter out key modules using the “WGCNA” package (version 1.70-3) in R. First, a proper soft thresholding power was selected to form an adjacency matrix and turn it into a topological overlap. Thereafter, the hierarchical clustering function was executed to plot the clustering trees. The genes in the TPM matrix were clustered into various modules by using a method called average linkage hierarchical clustering. The least gene number in every module was 50. The threshold to merge similar modules was set at 0.3. Positive or negative correlation modules with *P*-value < 0.05 were candidates for further analyses.

### Identification of Differentially Expressed Genes

An intersection between positive correlation modules of WGCNA in GSE55492 and GSE148219 and the differentially expressed mRNAs of GSE55492 and GSE14821 were illustrated using Venn diagrams. The same was done using the negative modules. The union set of these two Venn diagrams was considered the DEGs. The intersection results of differentially expressed LncRNAs in GSE55492 and GSE148219 were selected to construct the LncRNA-miRNA-mRNA regulatory network.

### Functional Enrichment Analysis

The clusterProfiler package (version 4.0.5) (9) was used to perform the Gene Ontology (GO) analysis, including biological processes (BPs), cellular components (CCs), molecular functions (MFs), and the Kyoto Encyclopedia of Genes and Genomes (KEGG) pathway enrichment analysis. *P-*values < 0.05 indicated statistical significance.

Metascape^4^ (10) was used to conduct and visualize pathway and process enrichment. DisGeNET (11) was used to exhibit the relationship between input genes and human diseases. Transcriptional regulatory relationships unraveled by sentence-based text-mining (TRRUST) database (12) integrated into Metascape were also applied to show the transcription factors of input genes.

### Protein-Protein Interaction and Hub Genes

Differentially expressed genes were uploaded into STRING^5^ to build a PPI network with a confidence score > 0.4, which was visualized by Cytoscape (version 3.8.2). A sub-network of the PPI network was then created using the plug-in, MCODE. The genes in subnetworks were picked up and further validated by plotting ROCs in the GSE12644 dataset. Genes with an area under the ROC curve (AUC) greater than 0.7 were considered hub genes associated with CAVD.

### Construction of the LncRNA-miRNA-mRNA Regulatory Network

The miRNA targets of differentially expressed LncRNAs were predicted using mircode^6^ and LncBase^7^. The miRNAs binding to the hub genes were predicted by four online databases: miRTarBase,^8^ miRDB,^9^ TargetScan,^10^ and mirDIP.^11^ After that, LncRNA-miRNA and miRNA-mRNAs pairs were uploaded and visualized by Cytoscape.

### Sample Collection and Cell Culture

Aortic valve tissues were collected from six severe patients with aortic stenosis and six patients having aortic valve regurgitation without obvious calcification during aortic valve replacement surgery. Patients with coronary heart disease, rheumatic aortic valve disease, congenital bicuspid aortic valve, infective endocarditis, hyperthyroidism, chronic renal dysfunction, smoking, and diabetes mellitus were excluded from this study. Supplementary Table 2 shows the clinical information of these 12 cases. One set of tricuspid aortic valves was then preserved at −80°C for qRT-PCR. Two sets of tricuspid aortic valves without obvious calcification were used to isolate aortic valve interstitial cells. Briefly, the aortic valves were first washed with PBS solution and subjected to 2 mg/ml collagenase II (Sigma, C6885) for 15 min. Epithelium cells in the valves were removed using a sterile cotton swab. After being cut using scissors, the valve tissues were digested again using collagenase II for another 2 h. The cell suspension was filtered, centrifuged, and resuspended with cell medium, including Dulbecco’s modified eagle medium (DMEM) (HyClone, SH30285.FS), 10% of fetal bovine serum (HyClone, SH30396.02), and 1% of penicillin streptomycin combination (HyClone, SV30010). All the experiments were conducted with 3–6 generations of cells. Supplementary Figure 1 shows the purity of valve interstitial cells by immunofluorescence. To induce osteogenic differentiation of aortic valve interstitial cells, the normal cell medium was replaced with an osteogenic medium, including 2 mmol/L of NaH_2_PO_4_ (Sangon Biotech, A100571-0100), 50 μg/ml of ascorbic acid (Sigma, A4403), and 10^–7^ mol/L of insulin (Novo Nordisk) for 7 days. Supplementary Figure 2 shows alizarin red staining for normal valve interstitial cells (VICs) and osteoblast-induced VICs on Day 7.

### Validation of Hub Genes and Long Non-coding RNAs in Competing Endogenous RNAnetwork of Calcified Aortic Valve Disease

The total RNA was extracted from aortic valve tissues and aortic valve interstitial cells with Trizol (Takara, Japan), quantified by Epoch Microporous plate spectrophotometer (BioTek, United States), and then reversely transcribed into cDNA by the PrimeScript™ RT Reagent Kit (Takara, Japan). A qRT-PCR was carried out with TB Green™ Premix Ex Taq™ (Takara, Japan) on an LC480 fluorescence quantitative PCR instrument (Roche, Switzerland). The relative expression level of genes was analyzed by the 2^–ΔΔ*CT*^ method. Table 1 shows all the primers bought from Sangon Biotech (Shanghai, China).

**TABLE 1:** List of primer sequences used for qRT-PCR.

Gene	Sequence
GAPDH	Forward: GCTCTCTGCTCCTCCTGTTC
	Reverse: ACGACCAAATCCGTTGACTC
LINC00702	Forward: TCAGCGTGTGTTCACATGGA
	Reverse: AACCCAAATCCCTGCCCATT
LINC01094	Forward: TGTAAAACGACGGCCAGT
	Reverse: CAGGAAACAGCTATGACC
LINC00092	Forward: TGCTGCTCTGGGGTTTTAAC
	Reverse: AAGGCCACTTCCAAACTGTG
TRHDE-AS1	Forward: TTCAACAGACTACAACCG
	Reverse: AGGCGAACTGGTGTAATA
SPP1	Forward: TGCCAGCAACCGAAGTTTTC
	Reverse: TGTCAGGTCTGCGAAACTTC
TREM1	Forward: TGCTGTGGATGCTCTTTGTC
	Reverse: TGCTGGCAAACTTCTCTAGC
GPM6A	Forward: ATGCATTGAGCCGCTCTTTG
	Reverse: ATGCATTGAGCCGCTCTTTG
CCL19	Forward: TGGGTACATCGTGAGGAACTTC
	Reverse: TCTGCAGTCTCTGGATGATGC
CR1	Forward: TCTTCCTGGGATGACAATCAGC
	Reverse: TCCAATTGGCTCCAGATTCCC
NCAM1	Forward: TGTTCAAGAATGCGCCAACC
	Reverse: ATGACATCTCGGCCTTTGTG
CNTN1	Forward: TTGAAGATCTTGGCGTTGGC
	Reverse: TCGGTGCAGCTTTAGGTTTG
TLR8	Forward: TGCAGAGCATCAACCAAAGC
	Reverse: TTAGCCTCTGCAAAGCCAAG
SDC1	Forward: TGGGGATGACTCTGACAACTTC
	Reverse: TTCTGGAGACGTGGGAATAGC
COL6A6	Forward: AAGCAGGATTTGGGAAAGGC
	Reverse: TTTCCTCGCTGCTTCTTTGC

### Immune Infiltration Analysis

The TPM matrix data of GSE55492 was uploaded to the CIBERSORT.^12^ The distribution of the 22 immune cells distribution in each sample and the expression level of immune cells between CAVD and control groups were exhibited by ggplot2 package in R. The Cor function in the Limma package was applied to analyze the correlation coefficients between hub genes and immune cells. The threshold for the correlation coefficient was 0.4.

## Results

### Results From Transcriptome Data Analysis

**FIGURE 2:**
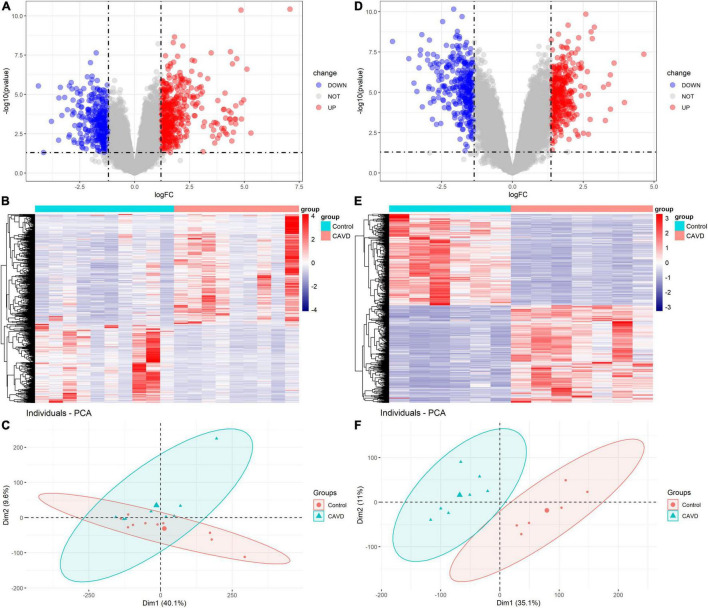
Volcano plots, heatmaps, and PCA plots of differentially expressed mRNAs. **(A)** Volcano plot of differentially expressed mRNAs in GSE55492; **(B)** Heatmap of differentially expressed mRNAs in GSE55492; **(C)** PCA plot in GSE55492; **(D)** Volcano plot of differentially expressed mRNAs in GSE148219; **(E)** Heatmap of differentially expressed mRNAs in GSE148219; **(F)** PCA plot in GSE149219.

In GSE55492, we filtered 370 upregulated mRNAs, 257 downregulated mRNAs, 19 upregulated LncRNAs, and 17 downregulated LncRNAs based on *P*-value < 0.05 and |log_2_FC| ≥ 1.20. In GSE148219, 365 upregulated mRNAs, 322 downregulated mRNAs, 7 upregulated LncRNAs, and 19 downregulated LncRNAs were identified according to *P*-value < 0.05 and |log_2_FC| ≥ 1.37. Supplementary Tables 3, 4 show the differentially expressed mRNAs and LncRNAs in GSE55492 and GSE148219, respectively. The volcano plot, heatmap, and PCA plot of differentially expressed mRNAs in GSE55492 and GSE148219 are shown in Figures 2A–F, respectively.

### Co-expression Network and Key Modules

The sample dendrogram and trait heatmap in GSE55492 are presented in Supplementary Figure 3A. The soft-thresholding power of 11 was selected (scale-free *R*^2^ = 0.92; Supplementary Figures 3B,C). The cluster dendrogram of the different samples is presented in Supplementary Figure 3D, whereas the gene dendrogram and module colors are provided in Supplementary Figure 3E. In GSE148219, the sample dendrogram and trait heatmap are shown in Supplementary Figure 4A. The soft-thresholding power of 12 was selected (scale-free *R*^2^ = 0.75; Supplementary Figures 4B,C). The cluster dendrogram of the different samples is presented in Supplementary Figure 4D, whereas the gene dendrogram and module colors are provided in Supplementary Figure 4E.

**FIGURE 3:**
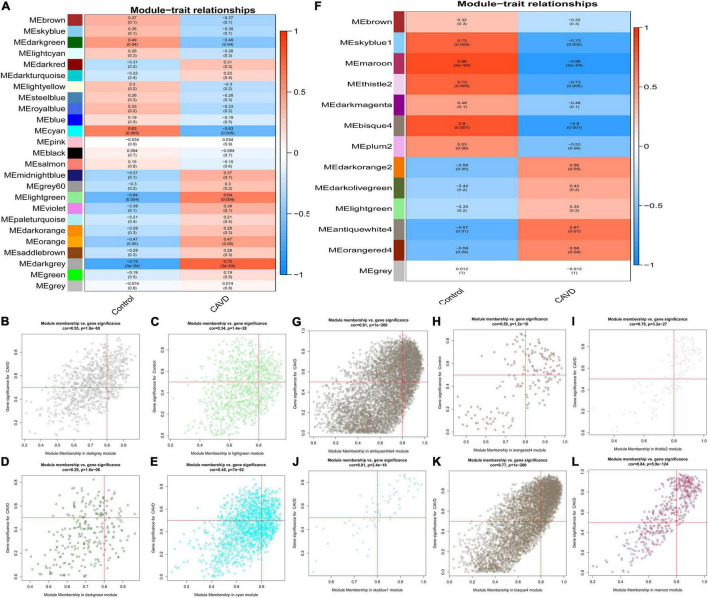
The module-trait correlation heatmap and specific modules. Blue represents negative correlation, and red represents positive correlation. **(A)** The correlation between modules and CAVD in GSE55492; **(B)** dark gray module in GSE55492, **(C)** light green module in GSE55492; **(D)** dark green module in GSE55492; **(E)** cyan module in GSE55492; **(F)** The correlation between modules and CAVD in GSE148219; **(G)** antique white 4 module in GSE148219; **(H)** orange red 4 module in GSE148219; **(I)** thistle 2 module in GSE148219; **(J)** sky blue 1 module in GSE148219; **(K)** bisque 4 module in GSE148219; **(L)** maroon module in GSE148219.

The association between gene modules and CAVD status were also analyzed. There were 25 modules in GSE55492 (Figure 3A). The dark-gray module (*r* = 0.75, *P* = 3e−04), light-green module (*r* = 0.64, *P* = 0.004), dark-green module (*r* = −0.49, *P* = 0.04), and cyan module (*r* = −0.63, *P* = 0.005) were considered the four associated modules with *P* < 0.05. The GSE148219 had 13 modules (Figure 3F). The antique white 4 module (*r* = 0.67, *P* = 0.01), orange red 4 module (*r* = 0.58, *P* = 0.04), thistle 2 module (*r* = −0.73, *P* = 0.005), sky blue 1 module (*r* = −0.73, *P* = 0.005), bisque 4 (*r* = −0.8, *P* = 0.001), and maroon module (*r* = −0.86, *P* = 0.0002) were identified as the six associated modules. The correlation between modules and gene significance were also shown as dark-gray (correlation coefficient = 0.55, *P* = 1.8e-69; Figure 3B), light-green (correlation coefficient = 0.34, *P* = 1.4e-28; Figure 3C), dark-green (correlation coefficient = 0.29, *P* = 1.6e-06; Figure 3D), cyan (correlation coefficient = 0.45, *P* = 7e-82; Figure 3E), antique white 4 (correlation coefficient = 0.61, *P* < 1e-200; Figure 3G), orange red 4 (correlation coefficient = 0.59, *P* < 1.2e-16; Figure 3H), thistle 2 (correlation coefficient = 0.78, *P* = 3.2e-27; Figure 3I), sky blue 1 (correlation coefficient = 0.81, *P* = 2.4e-18; Figure 3J), bisque 4 (correlation coefficient = 0.77, *P* < 1e-200; Figure 3K), and maroon (correlation coefficient = 0.84, *P* < 5.9e-124; Figure 3L).

### Functional Enrichment Analysis of Differentially Expressed Genes in Calcified Aortic Valve Disease

**FIGURE 4:**
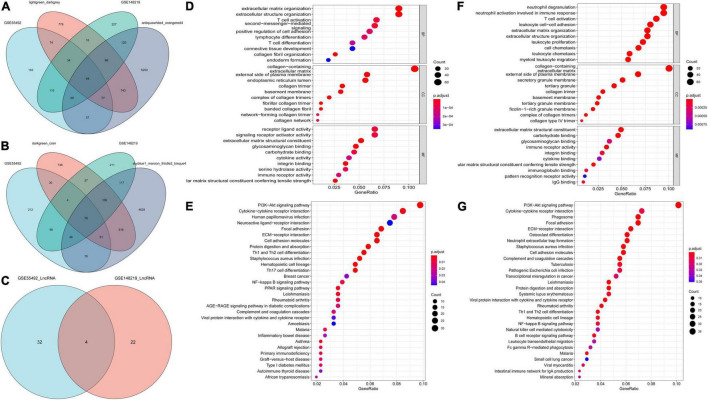
Differentially expressed mRNAs and LncRNAs for CAVD, and the functional enrichment analysis. **(A)** Venn diagram for differentially expressed mRNAs and positive modules in GSE55492 and GSE148219; **(B)** Venn diagram for differentially expressed mRNAs and negative modules in GSE55492 and GSE148219; **(C)** Venn diagram for differentially expressed LncRNAs in GSE55492 and GSE148219; **(D)** GO analyses of differentially expressed mRNAs in GSE55492; **(E)** KEGG pathway analyses of differentially expressed mRNAs in GSE55492; **(F)** GO analyses of differentially expressed mRNAs in GSE148219; **(G)** KEGG pathway analyses of differentially expressed mRNAs in GSE148219.

After considering the intersection among differentially expressed mRNAs in GSE55492, GSE148219, positive, or negative modules resulting from WGCNA, we screened out 126 differentially expressed mRNAs (Figures 4A,B), which were considered DEGs in CAVD. Four differentially expressed LncRNAs, namely TRHDE-AS1, LINC00092, LINC01094, and LINC00702, overlapped in these two datasets (Figure 4C).

Based on the clusterProfiler package, GO and KEGG were used to elucidate the biological function of the genes that were picked up. As shown in Figure 4D, the BP for differentially expressed mRNAs in GSE55492 were most enriched during extracellular matrix organization, extracellular structure organization, T-cell activation, second-messenger-mediated signaling, and positive regulation of cell adhesion. The CC terms were mostly focused on the regulation of dendritic cell dendrite assembly, collagen-containing extracellular matrix, external side of the plasma membrane, endoplasmic reticulum lumen, and membrane raft. The five enriched MF annotations included receptor ligand activity, signaling receptor activator activity, extracellular matrix structural constituent, glycosaminoglycan binding, and endopeptidase activity. The KEGG pathway analysis revealed that differentially expressed mRNAs in GSE55492 were significantly enriched in the PI3K-AKT signaling pathway, cytokine-cytokine receptor interaction, human papillomavirus infection, neuroactive ligand-receptor interaction, and focal adhesion (Figure 4E).

In GSE148219, the five most enriched BP terms were neutrophil degranulation, neutrophil activation involved in immune response, T-cell activation, leukocyte cell-cell adhesion, and positive regulation of cytokine production. Collagen-containing extracellular matrix, external side of the plasma membrane, secretory granule membrane, secretory granule lumen, and cytoplasmic vesicle lumen were the CC terms with the most enriched genes. The enriched MF were mainly involved in extracellular matrix structural constituent, carbohydrate-binding, G protein-coupled receptor binding, phosphoric ester hydrolase activity, and glycosaminoglycan binding (Figure 4F). The KEGG pathway analysis shows that the PI3K-Akt signaling pathway and cytokine-cytokine receptor interaction were the most enriched pathways, followed by phagosome, focal adhesion, and human papillomavirus infection (Figure 4G).

**FIGURE 5:**
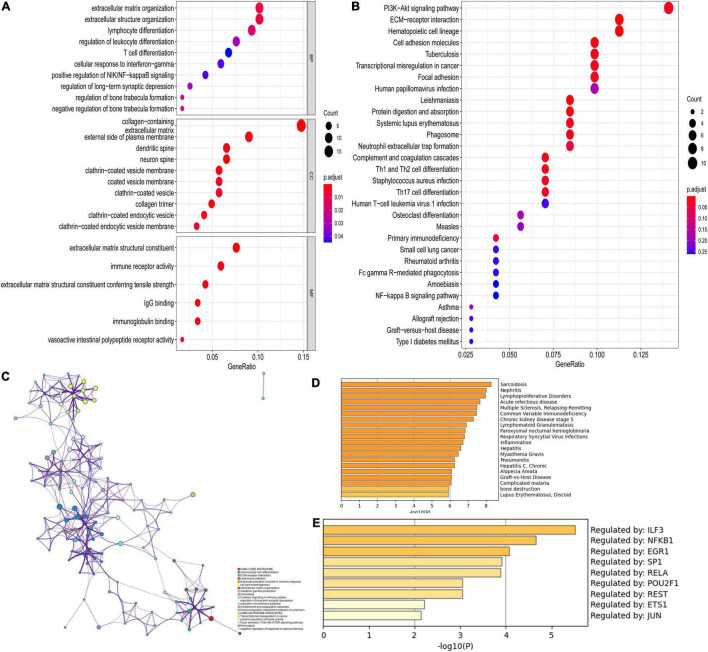
Functional enrichment analysis of DEGs. **(A)** GO analysis for DEGs; **(B)** KEGG pathway enrichment analysis for DEGs; **(C)** The network for enriched terms by Metascape; **(D)** The results of DisGeNET database; **(E)** The results of transcriptional regulatory relationships unraveled by sentence-based text-mining (TRRUST) database.

Functional enrichment analyses were also performed in DEGs for CAVD. As shown in Figure 5A, the top five BP terms were enriched in extracellular matrix organization, extracellular structure organization, lymphocyte differentiation, regulation of leukocyte differentiation, and T-cell differentiation. The top five CC terms were enriched in the collagen-containing extracellular matrix, external side of the plasma membrane, dendritic spine, neuron spine, and coated vesicle. The top five enriched MF terms included extracellular matrix structural constituent, immune receptor activity, extracellular matrix structural constituent conferring tensile strength, IgG binding, and immunoglobulin binding. The KEGG pathways were mostly enriched in PI3K-Akt signaling pathway, ECM-receptor interaction, hematopoietic cell lineage, cell adhesion molecules, and focal adhesion (Figure 5B). More details of enrichment results for DEGs are presented in Table 2.

**TABLE 2:** The GO and KEGG functional enrichment analyses of DEGs.

ID	Description	Count	Gene ID	*P*-value
**BP**				
GO:0030198	Extracellular matrix organization	12	FAP, SPP1, GREM1, ITGAX, ANGPTL7, COL4A3, COL9A1, CHADL, COL28A1, LAMC3, COL4A4, VIT	6.96E-06
GO:0043062	Extracellular structure organization	12	FAP, SPP1, GREM1, ITGAX, ANGPTL7, COL4A3, COL9A1, CHADL, COL28A1, LAMC3, COL4A4, VIT	7.14E-06
GO:0030098	Lymphocyte differentiation	11	CCL19, CR1, CD2, SLAMF8, DOCK10, CD3D, BCL11B, IL7R, CD3E, POU2AF1, ZBTB16	1.99E-05
GO:1902105	Regulation of leukocyte differentiation	9	CCL19, CR1, GPR68, CD2, SLAMF8, HLA-DRB1, TREM2, IL7R, ZBTB16	8.76E-05
GO:0030217	T cell differentiation	8	CCL19, CR1, CD2, CD3D, BCL11B, IL7R, CD3E, ZBTB16	1.92E-04
**CC**				
GO:0062023	Collagen-containing extracellular matrix	18	CTHRC1, SERPINA1, GREM1, COL6A6, EMILIN3, ANGPTL7, CBLN1, COL4A3, MYOC, COL9A1, CHADL, NCAM1, COL28A1, LAMC3, COL4A4, COCH, VIT, CLEC3B	1.66E-10
GO:0009897	External side of plasma membrane	11	FCGR3A, TLR8, CD2, IL2RB, HLA-DRB1, CD3D, IL7R, CD3E, SDC1, NCAM1, CNTFR	6.11E-05
GO:0043197	Dendritic spine	8	DOCK10, CD3E, FBXO2, MAPT, PPP1R9A, PPP1R1B, ATP1A2, GPM6A	1.42E-05
GO:0044309	Neuron spine	8	DOCK10, CD3E, FBXO2, MAPT, PPP1R9A, PPP1R1B, ATP1A2, GPM6A	1.54E-05
GO:0030135	Coated vesicle	8	FCGR1A, CEMIP, SERPINA1, FCGR1B, HLA-DRA, HLA-DRB1, CD3D, IL7R	5.68E-04
**MF**				
GO:0005201	Extracellular matrix structural constituent	9	CTHRC1, CHI3L1, COL6A6, EMILIN3, COL4A3, COL9A1, CHADL, COL28A1, COL4A4	1.46E-06
GO:0140375	Immune receptor activity	7	CR1, FCGR1B, HLA-DRA, IL2RB, HLA-DRB1, IL7R, CNTFR	2.67E-05
GO:0030020	Extracellular matrix structural constituent conferring tensile strength	5	COL6A6, COL4A3, COL9A1, COL28A1, COL4A4	6.29E-06
GO:0019864	IgG binding	4	FCGR3A, FCGR1A, FCGR1B, FCGR3B	5.18E-07
GO:0019865	Immunoglobulin Binding	4	FCGR3A, FCGR1A, FCGR1B, FCGR3B	1.56E-05
**KEGG**				
hsa04151	PI3K-Akt signaling pathway	10	SPP1, IL2RB, IL7R, COL6A6, ERBB3, COL4A3, COL9A1, LAMC3, CHAD, COL4A4	9.60E-04
hsa04640	Hematopoietic cell lineage	8	FCGR1A, CR1, CD2, HLA-DRA, HLA-DRB1, CD3D, IL7R, CD3E	2.12E-06
hsa04512	ECM-receptor interaction	8	SPP1, SDC1, COL6A6, COL4A3, COL9A1, LAMC3, CHAD, COL4A4	8.59E-07
hsa04514	Cell adhesion molecules	7	CD2, HLA-DRA, HLA-DRB1, SDC1, CNTN1, IGSF11, NCAM1	3.08E-04
hsa04510	Focal adhesion	7	SPP1, COL6A6, COL4A3, COL9A1, LAMC3, CHAD, COL4A4	1.82E-03

Moreover, Figure 5C shows the top 20 pathway enrichment using Metascape, such as ECM-receptor interaction, extracellular matrix organization, and focal adhesion: PI3K-Akt-mTOR-signaling pathway and cytokine signaling in the immune system. Enrichment analysis by DisGeNET showed that DEGs were significantly associated with sarcoidosis, nephritis, acute infectious disease, lymphoproliferative disorders, and others (Figure 5D). The TRRUST database provides various transcription factors regulating DEGs, including LIF3, NFKB1, EGR1, SP1, RELA, POU2F1, REST, ETS1, and JUN (Figure 5E).

### Protein-Protein Interaction Network Analysis and Hub Genes Associated With Calcified Aortic Valve Disease

**FIGURE 6:**
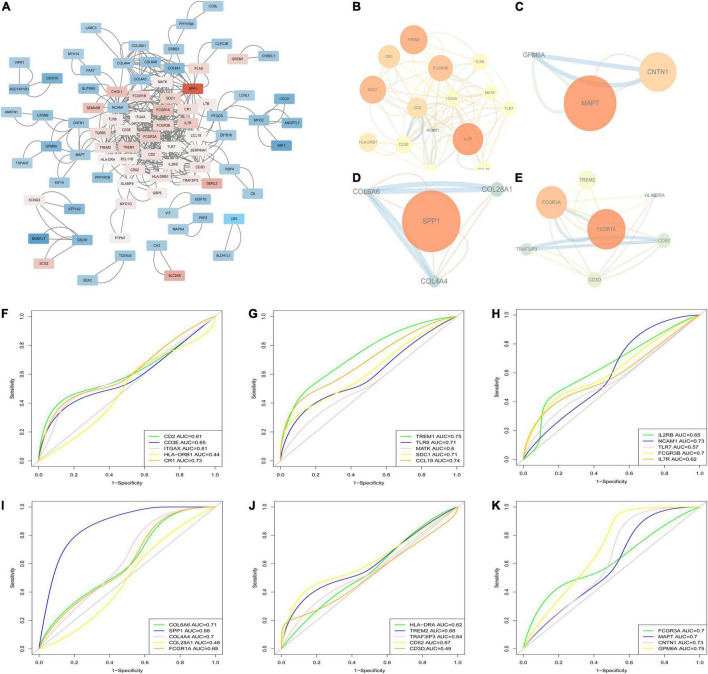
PPI network and hub genes identification. **(A)** The PPI network of DEGs; **(B–E)** The four sub-networks of the PPI network; **(F–K)** Receiver operating characteristic curve (ROC) for the genes in sub-networks in GSE12644.

The PPI network, which consists of 386 edges and 126 nodes, was visualized via the Cytoscape software (Figure 6A). A total of four sub-networks of the PPI network were filtered out via MCODE plug-in, and are shown in Figures 6B–E. A total of 29 genes in the four sub-networks were further validated by applying ROC analyses in GSE12644 (Figures 6F–K), including CD2 (AUC = 0.61), CD3E (AUC = 0.65), ITGAX (AUC = 0.61), HLA-DRB1 (AUC = 0.44), CR1 (AUC = 0.73), TREM1 (AUC = 0.75), TLR8 (AUC = 0.71), MATK (AUC = 0.6), SDC1 (AUC = 0.71), CCL19 (AUC = 0.74), IL2RB (AUC = 0.65), NCAM1 (AUC = 0.73), TLR7 (AUC = 0.57), FCGR3B (AUC = 0.7), IL7R (AUC = 0.62), COL6A6 (AUC = 0.71), SPP1 (AUC = 0.88), COL4A4 (AUC = 0.7), COL28A1 (AUC = 0.48), FCGR1A (AUC = 0.68), HLA-DRA (AUC = 0.62), TREM2 (AUC = 0.68), TRAF3IP3 (AUC = 0.64), CD52 (AUC = 0.67), CD3D (AUC = 0.49), FCGR3A (AUC = 0.7), MAPT (AUC = 0.7), CNTN1 (AUC = 0.73), and GPM6A (AUC = 0.75). Based on the results of ROC, 10 genes (CR1, TREM1, TLR8, SDC1, CCL19, NCAM1, COL6A6, SPP1, CNTN1, and GPM6A) with AUC > 0.7 were considered the hub genes associated with CAVD.

### Construction of the Potential LINC00702-miR-181b-5p-SPP1 Axis in Calcified Aortic Valve Disease

**FIGURE 7:**
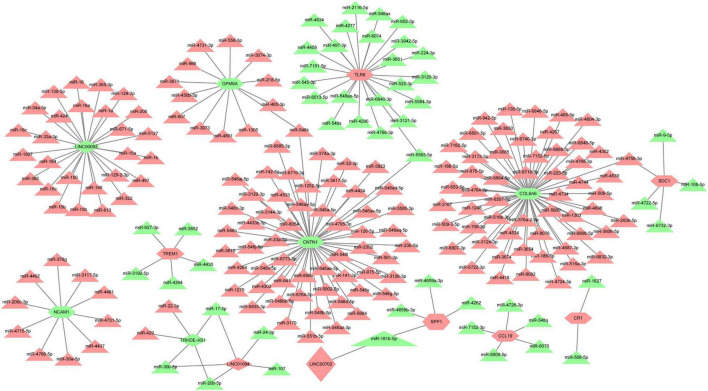
The LncRNA-miRNA-mRNA regulatory network for CAVD. Red color means upregulated genes, and green color represents downregulated genes. Rhombus represents LncRNAs, triangles represent miRNAs, and shuttle shapes represent mRNAs.

Based on the prediction of online databases, a total of 38 LncRNA-miRNA pairs and 181 miRNA-mRNAs pairs were confirmed. The LncRNA-mediated regulatory network was constructed via Cytoscape software and are exhibited in Figure 7. Interestingly, miR-181b-5p was the downstream target of LINC00702, whereas SPP1 was the target gene of miR-181b-5p. According to the competing endogenous (ceRNA) theory, we speculated that LINC00702-miR-181b-5p-SPP1 axis might participate in the process of CAVD development, which deserves further experimental validation.

### Validation Results

**FIGURE 8:**
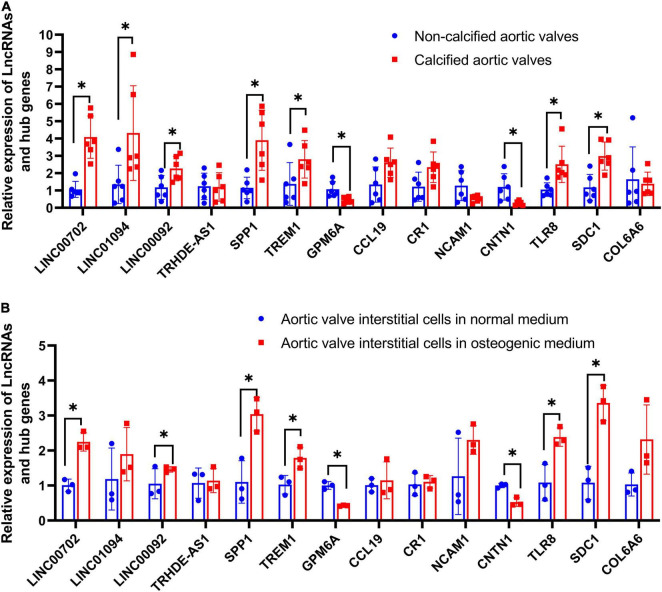
Validation of four LncRNAs and hub genes. **(A)** The relative expression of LncRNAs and hub genes in clinical samples; **(B)** The relative expression of LncRNAs and hub genes in aortic valve interstitial cells. *P < 0.05.

The relative expression levels of four LncRNAs and 10 hub genes were detected in aortic valve samples and valve interstitial cells by qRT-PCR. Figure 8A reveals that calcified aortic valve tissues had higher expression levels of LINC00702, LINC01094, LINC00092, SPP1, TREM1, TLR8, and SDC1 than noncalcified aortic valves and that calcified aortic valve had lower expression levels of GPM6A and CNTN1 than the noncalcified aortic valve. Meanwhile, no statistically significant differences between the two groups were observed in the expression levels of TRHDE-AS1, CCL19, CR1, NCAM1, and COL6A6.

Osteogenic-induced aortic valve interstitial cells had higher expression levels of LINC00702, LINC00092, SPP1, TREM1, TLR8, and SDC1 than normal aortic valve interstitial cells. Meanwhile, osteogenic-induced aortic valve interstitial cells had lower expression levels of GPM6A and CNTN1 than normal aortic valve interstitial cells. Moreover, no statistically significant differences were observed in the expression levels of LINC01094, TRHDE-AS1, CCL19, CR1, NCAM1, and COL6A6 (Figure 8B).

In conclusion, statistically significant differences in LINC00702, LINC00092, SPP1, TREM1, TLR8, SDC1, GPM6A, and CNTN1 were observed in both clinical samples and valve interstitial cells.

### Immune Cell Infiltration of Calcified Aortic Valve Disease

**FIGURE 9:**
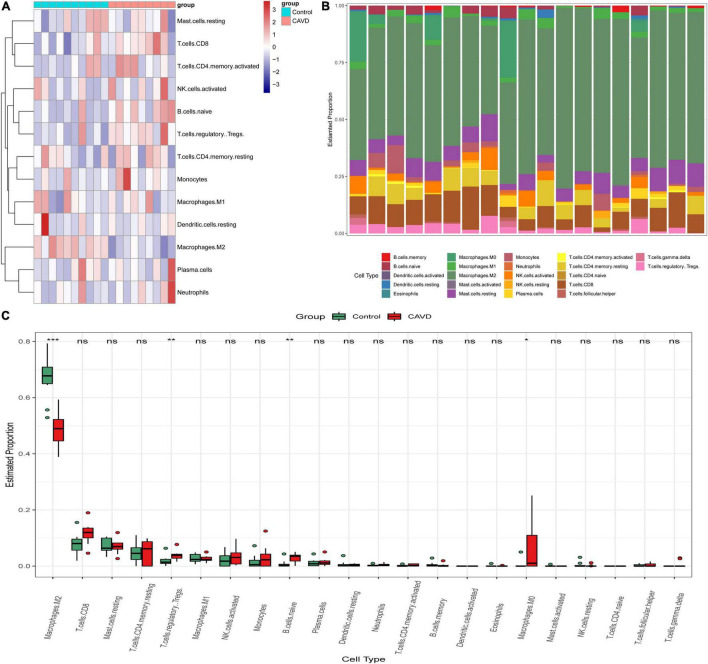
Immune infiltration analysis in GSE55492 by CIBERSORT. **(A)** Heatmap of various immune cells in each sample. **(B)** The bar plot of various immune cell distributions in each sample. **(C)** Differences in immune infiltration between calcified aortic valve samples and normal samples by the Wilcox test. Red represents calcified aortic valve samples, and green represents normal samples. The *x*-axis shows the different types of immune cells, and the *y*-axis shows the proportion of immune cells. *P < 0.05; **P < 0.01; ***P < 0.001.

**FIGURE 10:**
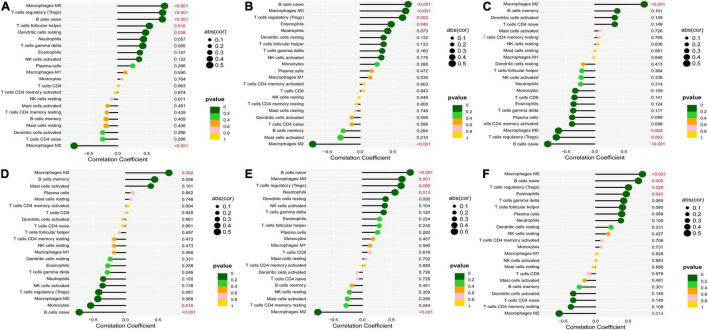
Correlation between validated hub genes and immune cells. **(A)** SPP1, **(B)** TREM1, **(C)** GPM6A, **(D)** CNTN1, **(E)** TLR8, and **(F)** SDC1. The *x-*axis represents the correlation coefficient, the left *y*-axis represents immune cells, the right *y*-axis represents *P*-value. The solid dots represent the relative quantity of the correlation coefficient. The color gradation represents the relative quantity of the *P*-valve. Pearson’s test was used for correlation analysis.

According to the results of CIBERSORT, the distribution of 22 immune cells between 9 calcified and 10 normal aortic valve samples were distinguished. Supplementary Table 5 shows the results of the distribution of immune cells in different samples. Figure 9A exhibits the proportion of 13 immune cells (cells with zero expression in half of the samples were deleted). The percentage of various immune cells in each sample was illustrated by the bar plot (Figure 9B). Notably, M2 macrophages accounted for the largest proportion in each valve tissue. The boxplot demonstrated that calcified aortic valves had higher levels of Tregs, naïve B cells, and M0 macrophages and lower levels of M2 macrophages compared with the normal valve tissues (Figure 9C). The correlation between validated hub genes and immune cells was also revealed, including SPP1 (Figure 10A), TREM1 (Figure 10B), GPM6A (Figure 10C), CNTN1 (Figure 10D), TLR8 (Figure 10E), and SDC1 (Figure 10F).

## Discussion

Evidence has suggested that CAVD is the major cause of aortic valve stenosis and has gained popularity owing to the aging of the population (13). Estimates have predicted that the disease burden in developed countries will double from 2000 to 2030 (14). For years, the progression of valve calcification had been considered a kind of irreversible degeneration during which calcium nodules are deposited on leaflets (15). With the improved understanding of disease mechanisms, valve calcification has been widely accepted to involve mixed pathophysiological progression, including endothelial dysfunction, lipid deposition, inflammation response, myofibroblastic and osteoblastic differentiation, calcific pathways activation, immune infiltration, and so on (15, 16).

To date, medical treatment for aortic valve stenosis is still lacking. According to the 2020 ACC/AHA Guideline for Valvular Heart Disease, surgical aortic valve replacement or transcatheter aortic valve replacement still remains the standard clinical practice (17). Despite the various studies involving CAVD, the extensive molecular mechanisms underlying CAVD remain poorly elucidated.

In this study, we filtered DEGs in CAVD based on two transcriptome datasets and WGCNA. The GO and KEGG were also utilized to identify specific biological processes. Our findings showed that DEGs associated with CAVD were mostly enriched in the extracellular matrix organization, immune cell activity, PI3K-AKT signaling pathway, ECM-receptor interaction, and cytokine-cytokine receptor interaction. To further explain the regulatory network and molecular mechanisms of CAVD, we filtered 10 hub genes (SPP1, TREM1, GPM6A, CCL19, CR1, NCAM1, CNTN1, TLR8, SDC1, and COL6A6), constructed the LncRNA-miRNA-mRNA network, and calculated the correlation between hub genes and immune cell infiltration, which has not been reported previously. We speculated that these hub genes and the LINC00702-miR-181b-5p-SPP1 axis might theoretically have an important function in CAVD.

Valve endothelial dysfunction induced by fluid shear stress would contribute to lipoprotein deposition, and immune cell infiltration, which had been considered to indicate the initiation of CAVD. SDC1 is a surface protein of the endothelial glycocalyx that could be a biomarker for endothelial damage (18). Notably, the level of plasma soluble SDC1 in patients with acute coronary syndrome has been associated with short-term mortality (18) and atherosclerotic plaque vulnerability (19). In a hyperlipemia-induced coronary atherosclerosis mice model, SDC1(−/−) mice showed hyperinflammation activity and greater atherosclerotic plaque disability (20). Moreover, previous research has reported that SDC1 was an important fluid shear stress sensor for vascular endothelial cells and participated in the regulation of endothelial phenotype. One study showed that loss of SDC1 facilitated a shift in endothelial cell phenotypic to an inflammatory phenotype (21).

Among the 10 hub genes, two cell adhesion molecules (NCAM1 and CNTN1) were conspicuous. Indeed, research has focused on adhesion-related signaling in coronary artery disease (CAD) (22). Yu P. et al., who compared the plasma level of NCAM1 in 429 coronary heart disease with 93 health controls, proved that the expression level of NCAM1 was significantly associated with cTnT and NT-proBNP, suggesting that NCAM1 may be a therapeutic target of CAD (23). Zhang and colleagues also confirmed that patients with myocardial infarction had dysregulated expression of NCAM1 (24). Considering the similar pathologic mechanisms between atherosclerosis and CAVD, we conjectured that NCAM1 might also be a new biomarker for aortic valve stenosis. The protein encoded by CNTN1 belongs to the immunoglobulin superfamily. To date, quite a few studies focused on the cancer regulation and neuropathy of CNTN1 (25–29). The role of CNTN1 in aortic stenosis has not been clarified, which needs further exploration.

Disease evolution has largely been attributed to injury and inflammation. Triantafilou K. et al. reported that coxsackie B viruses could cause human cardiac inflammatory responses in an TLR8-dependent way (30). Kapelouzou et al. who determined the relationship between atherosclerotic lesions and TLRs mRNA expression in the hypercholesterolemic diet rabbit model, proved that TLR8 mRNA expression was strongly upregulated and correlated with aorta atherosclerosis (31). Qiao E and his colleagues also filtered out TLR8 as one of the hub genes associated with CAVD based on four microarray datasets via bioinformatics analysis (32), which was consistent with the findings obtained in the current study.

The present study showed that most of the DEGs were enriched in the PI3K-AKT pathway. As widely known, this pathway participates in the development of various diseases and has been associated with apoptosis, cell proliferation, and metabolism (33–36). Multiple agents or drugs could activate this signaling pathway, including SPP1 (also known as osteopontin) (37–41). In fact, SPP1 has been identified as one of the ossification biological makers (6, 41–43). COL6A6, one of the three collagen VI genes, is widely expressed in the extracellular matrix of the cardiac muscle, lung, and territorial matrix of articular cartilages (44, 45). Through whole-exome sequencing, Gari et al. reported that a genetic variant of COL6A6 could influence osteoarthritis disease (46). In another sequencing analysis, deleterious mutations of COL6A6 contribute to the ossification of the posterior longitudinal ligament (47). After establishing an *in vitro* aortic valve interstitial cell calcification model, Bouchareb et al. found that COL6A6 was one of the extracellular matrix proteins expressed at the early stage (48). Bosse et al. performed the microarray sequence on five normal aortic valves with five stenosis valves and reported that GPM6A was one of the 10 most downregulated DEG (49), which was confirmed in the hypercholesterolemic induced aortic valve calcification mice model (50).

Adaptive and innate immune cell signaling in CAVD could be the potential therapeutic targets (51). TREM1 is a potent amplifier of pro-inflammatory innate immune responses (52). Loss of TREM1 may constitute a new treatment for atherosclerosis by regulating foam cell formation and monocyte/macrophage proinflammatory responses (53). The plasma expression level of soluble TREM1 has been considered one of the predictors of mortality in patients with acute myocardial infarction (54). Furthermore, TREM1 signaling was one of the top five enriched pathways based on peripheral blood gene analysis from patients with aortic valve stenosis (55). Deng et al. reported that Complement C3 could upregulate RUNX2 to induce ossification of aortic valve interstitial cells (56). In our study, the expression level of complement C3b/C4b receptor 1 (CR1) was also elevated in the CAVD group.

Additionally, the activation of cytokine signaling was also one of the mechanisms of aortic valve calcification (16). CCL19, as one of the cytokines, could regulate the inflammation and matrix remodeling in atherosclerosis and rheumatoid arthritis (57, 58). Yiran Zhang et al. revealed that patients with CAVD had increased CCL19 expression (59). Based on previous studies and the results of the current study, we believed that the role of CCL19 in the CAVD warrants further exploration.

Finally, we constructed the LINC00702-miR-181b-5p-SPP1 axis and showed that this signal axis could regulate the development of CAVD. As one of the miRNA sponges, LINC00702 could interact with different miRNAs in various diseases (60–63). By constructing a cardiac hypertrophy-related LncRNA-mRNA network, Song et al. reported that LINC00702 was one of the ten hub nodes (64). Wang et al. aimed to investigate the underlying mechanisms of unstable atherosclerotic plaque rupture and identified that LINC00702 was also dysregulated in ruptured atherosclerotic samples (65). In the aging-related aortic artery stiffness (66), atherosclerosis plaque stability (67), endothelial dysfunction (68), and abdominal aortic aneurysm (69), miR-181b exhibited important regulatory function. Another research showed that circulating miR-181b level was positively associated with myocardium function in diabetic cardiomyopathy (70). miRNA-181b could also act as the fluid shear responsiveness molecular, regulating the microenvironment of aortic valve endothelial cells by targeting TIMP3 (71). Although the independent roles of LINC00702, miR-181b-5p, and SPP1 in CAVDs have been clarified, this study has been the first to speculate on the role of the LINC00702-miR-181b-5p-SPP1 axis in CAVD, which warrants further verification.

### Limitations

The current study has several limitations worth noting. First, this study utilized bioinformatic analysis of transcriptome data, and mRNA expression is not equal to protein expression. Further proteomics of samples obtained from humans or animals is therefore recommended. Second, bioinformatics analysis cannot replace experimental validation. Hence, a well-designed *in vitro* and *in vivo* exploration should be conducted to ascertain the hub genes and immune cell regulation in CAVD.

## Conclusion

The current study suggested the LINC00702–miR-181b-5p–SPP1 axis could participate in the development and progression of CAVD and that LINC00702, LINC00092, SPP1, TREM1, TLR8, SDC1, GPM6A, and CNTN1 might be the hub molecules associated with CAVD. Additionally, we speculated that Tregs, naïve B cells, M0 macrophages, and M2 macrophages could possibly be involved in the initiation of CAVD.

## Data Availability Statement

The datasets presented in this study can be found in online repositories. The names of the repository/repositories and accession number(s) can be found in the article/Supplementary Material.

## Ethics Statement

The studies involving human participants were reviewed and approved by Medical Ethics Committee of Changhai Hospital, Second Military Medical University, Shanghai, China. The patients/participants provided their written informed consent to participate in this study.

## Author Contributions

LH and XL conceived the ideas and supervised the study. KH, LW, and YG analyzed the data. QL and HW provided software support. KH wrote the manuscript. LH was responsible for the overall content as the guarantor. All authors have read and approved the final version for publication.

## Conflict of Interest

The authors declare that the research was conducted in the absence of any commercial or financial relationships that could be construed as a potential conflict of interest.

## Publisher’s Note

All claims expressed in this article are solely those of the authors and do not necessarily represent those of their affiliated organizations, or those of the publisher, the editors and the reviewers. Any product that may be evaluated in this article, or claim that may be made by its manufacturer, is not guaranteed or endorsed by the publisher.

## Footnotes

1
www.ebi.ac.uk/ena

2
www.ncbi.nlm.nih.gov/gds

3
www.ensembl.org/

4
metascape.org

5
string-db.org/

6
www.mircode.org/

7
https://diana.e-ce.uth.gr/lncbasev3/home

8
mirtarbase.cuhk.edu.cn/

9
mirdb.org/

10
www.targetscan.org/

11
https://ophid.utoronto.ca/mirDIP/

12
cibersort.stanford.edu
