# Supplementary material for: Transcriptome Sequencing Data Reveal LncRNA-miRNA-mRNA Regulatory Network in Calcified Aortic Valve Disease
Source: Front Cardiovasc Med. 2022 May 26;9:886995. doi: 10.3389/fcvm.2022.886995 (PMC9204424; doi:10.3389/fcvm.2022.886995)
Supplement: Supplementary file 1 [file Data_Sheet_1.PDF]

Supplementary Table 1 Demographic data of cases in three GEO datasets

GSE55492			
geo accession	Group	Age	Sex
GSM1338133	Control	56	Male
GSM1338134	Control	67	Male
GSM1338135	Control	66	Male
GSM1338136	Control	65	Male
GSM1338137	Control	68	Male
GSM1338138	Control	68	Male
GSM1338139	Control	53	Male
GSM1338140	Control	63	Male
GSM1338141	Control	59	Male
GSM1338142	Control	58	Male
GSM1338124	CAVD	68	Male
GSM1338125	CAVD	71	Male
GSM1338126	CAVD	64	Male
GSM1338127	CAVD	60	Male
GSM1338128	CAVD	59	Male
GSM1338129	CAVD	70	Male
GSM1338130	CAVD	58	Male
GSM1338131	CAVD	60	Male
GSM1338132	CAVD	60	Male
**GSE148219**			
geo accession	Group	Age	Sex
GSM4456260	Control	30	male
GSM4456261	Control	17	male
GSM4456262	Control	29	male
GSM4456272	Control	38	female
GSM4456273	Control	31	male
GSM4456274	Control	47	male
GSM4456263	CAVD	76	male
GSM4456264	CAVD	79	male
GSM4456265	CAVD	69	male
GSM4456267	CAVD	79	male
GSM4456268	CAVD	68	male
GSM4456269	CAVD	57	male
GSM4456270	CAVD	72	male
**GSE12644**			
geo accession	Group	Age	Sex
GSM317342	Control	65	male
GSM317343	Control	73	male
GSM317344	Control	62	male
GSM317345	Control	59	male
GSM317346	Control	58	male

GSM377368	Control	32	male
GSM377369	Control	51	male
GSM377370	Control	58	male
GSM377371	Control	67	male
GSM377372	Control	61	male
GSM317347	CAVD	70	male
GSM317348	CAVD	74	male
GSM317349	CAVD	69	male
GSM317350	CAVD	64	male
GSM317351	CAVD	60	male
GSM377373	CAVD	48	male
GSM377374	CAVD	51	male
GSM377375	CAVD	68	male
GSM377376	CAVD	56	male
GSM377377	CAVD	65	male

Supplementary Table 2 Basic information of 12 patients collected for experiment

Parameters	Control group (n=6)	CAVD group (n=6)	*P* valve
Age (years)	57.83±7.49	60.33±5.16	0.516
Male, n (%)	4 (66.7)	4 (66.7)	1.000
BMI (kg/m^2)	22.99±1.05	25.34±1.69	0.016*
Complications			
Hyperlipidemia, n(%)	1 (16.7)	4 (66.7)	0.092
Hypertension, n(%)	3 (50)	4 (66.7)	0.599
Degree of aortic valve calcification	1.33±0.52	4.50±0.55	< 0.001***

Aortic valve calcification is defined as the bright echo spots in valves by echocardiography. A single total score is assigned to the whole aortic valves. Normal and non-thickened valves are classified as grade 1. The thickened but non-calcified valves are grade 2. Mild calcified valves (< 1/3 of the valve leaflets) are grade 3. Moderate calcified valves (<2/3 of the valve leaflets) are grade 4. Severe calcified valves (> 2/3 area) are grade 5. * $P < 0.05$, ** $P < 0.01$, *** $P < 0.001$.

Supplementary Table 3 The differentially expressed mRNAs and LncRNAs in GSE55492

mRNA	Log FC	AveExpr	t	P.Value	adj.P.Val	B	change
ABCB1	1.472552	0.143307	3.933214	0.00071	0.01572	-0.3622	UP
ABCC8	-1.89153	-0.25062	-2.66637	0.014102	0.099423	-2.98555	DOWN

ACAN	3.096897	4.918136	5.850436	6.92E-06	0.001054	3.806197	UP
ACE	1.76213	2.019962	4.372783	0.000243	0.008123	0.528102	UP
ACTA2	1.37818	10.02945	4.14166	0.000427	0.011291	-0.24176	UP
ACTG2	1.973509	4.831199	2.501705	0.020292	0.125053	-3.93649	UP
ADAM12	1.507109	2.536906	3.262552	0.003565	0.042676	-2.07603	UP
ADAM28	1.318883	2.769344	5.50925	1.55E-05	0.001683	3.11495	UP
ADAM8	1.322679	3.307577	3.404875	0.002542	0.034845	-1.85096	UP
ADAMDEC1	3.022195	-0.17643	5.720556	9.39E-06	0.001268	3.496503	UP
ADAMTS12	1.427977	2.447079	5.900123	6.16E-06	0.001019	4.019104	UP
ADAMTS14	1.444546	2.829135	3.953819	0.000675	0.015194	-0.52928	UP
ADAMTS16	1.857432	-0.25008	5.0871	4.27E-05	0.002892	2.157096	UP
ADARB2	-1.5622	-0.54248	-2.1477	0.043011	0.194598	-3.91598	DOWN
ADCYAP1	2.157076	-1.17141	4.371071	0.000244	0.008129	0.568351	UP
ADCYAP1R1	-1.9295	2.436199	-6.85948	6.87E-07	0.000424	6.111114	DOWN
ADD2	1.378893	-0.24892	4.066726	0.000513	0.01276	-0.06229	UP
ADGRB3	-1.33576	1.336576	-3.95689	0.00067	0.015139	-0.36491	DOWN
ADGRD2	-2.83325	-0.40551	-4.46488	0.000194	0.0071	0.790102	DOWN
ADGRE1	1.441092	-1.12048	3.248049	0.003689	0.043663	-1.78984	UP
ADGRE2	1.317488	2.609182	3.727155	0.001171	0.021235	-1.03123	UP
ADGRL3	1.440839	0.211459	3.606508	0.001567	0.025523	-1.08285	UP
ADGRL4	1.503753	2.216337	3.383617	0.002674	0.035483	-1.76453	UP
ADM2	1.3121	-0.62071	3.500522	0.002022	0.029983	-1.27697	UP
ADRA2A	2.672207	3.08353	5.590737	1.28E-05	0.001527	3.29565	UP
AGTR1	-2.97524	-0.70039	-6.22272	2.91E-06	0.00078	4.372945	DOWN
ALDH1L1	-1.5513	3.586054	-3.64634	0.001423	0.023921	-1.30291	DOWN
ALK	-1.50168	-0.39909	-2.89929	0.008319	0.072471	-2.52001	DOWN
ALPK2	1.852243	-0.46445	2.635481	0.015107	0.104332	-3.04633	UP
AMER2	-2.72219	-0.51284	-3.80381	0.000972	0.01889	-0.63011	DOWN
AMH	-1.2565	0.571514	-4.52837	0.000166	0.006398	0.957016	DOWN
AMHR2	-1.4791	-0.42002	-2.86032	0.009096	0.076428	-2.59691	DOWN
AMPD3	1.244216	3.128024	4.862783	7.35E-05	0.003942	1.580655	UP
ANGPT2	1.87421	2.524236	5.177794	3.43E-05	0.002622	2.365295	UP
ANGPTL4	-1.45407	4.909203	-4.89689	6.77E-05	0.003713	1.543631	DOWN
ANGPTL7	-2.56956	5.616268	-5.02853	4.92E-05	0.00309	1.842479	DOWN
ANKFN1	-1.24682	2.235602	-6.28382	2.53E-06	0.000729	4.874496	DOWN
ANO5	-2.05988	-0.68741	-5.58882	1.28E-05	0.001527	3.131492	DOWN
APBA2	1.424456	2.304736	6.385313	2.00E-06	0.000646	5.09698	UP
APCDD1L	1.985483	3.416155	5.703871	9.77E-06	0.001283	3.527809	UP
APLN	1.29979	2.522237	2.853059	0.009248	0.077152	-2.9629	UP
APOA1	-2.65315	2.728863	-6.05242	4.31E-06	0.000875	4.364372	DOWN
AQP2	-1.19903	3.354296	-3.4719	0.002165	0.031355	-1.68482	DOWN
AQP7	-2.80694	2.270883	-4.69575	0.00011	0.004918	1.292365	DOWN
ARL4C	1.466303	4.888947	5.067551	4.47E-05	0.002942	1.939463	UP
ARNTL2	1.446759	1.809574	6.021508	4.64E-06	0.000905	4.296622	UP

ASTN1	-2.10245	0.612306	-4.74884	9.70E-05	0.004582	1.447546	DOWN
ATP1A2	-2.47671	5.125693	-5.88975	6.31E-06	0.001026	3.889738	DOWN
ATRNL1	-1.75051	1.854081	-8.46612	2.28E-08	4.83E-05	9.212182	DOWN
AZGP1	-2.76392	0.173885	-2.91656	0.007996	0.070967	-2.51764	DOWN
B3GAT1	-1.59281	-1.08851	-3.05224	0.005841	0.057817	-2.18281	DOWN
BCAS1	-1.41924	-0.73055	-2.39804	0.025403	0.143372	-3.45666	DOWN
BCL11B	1.29926	1.252308	4.990262	5.39E-05	0.003272	1.988632	UP
BCL2A1	1.593335	-0.64852	2.465448	0.021959	0.131418	-3.35165	UP
BEX2	-1.51557	3.201145	-4.40244	0.000226	0.00776	0.505411	DOWN
BGLAP	1.685396	0.197189	6.060749	4.23E-06	0.000875	4.265228	UP
BMX	-1.44196	4.188597	-4.1441	0.000424	0.01124	-0.19782	DOWN
BTG2	-1.2493	8.707315	-3.30897	0.003193	0.039754	-2.23424	DOWN
BUB1	1.219552	0.243861	3.044913	0.005942	0.058575	-2.28712	UP
C10orf82	-1.37078	-1.01421	-3.92966	0.000716	0.015779	-0.36709	DOWN
C15orf48	1.911342	0.147872	2.865958	0.008979	0.076101	-2.64474	UP
C1QTNF3	1.691457	3.351385	4.867675	7.26E-05	0.003923	1.579817	UP
C2orf72	-1.5551	-0.90297	-3.85077	0.000867	0.017671	-0.5322	DOWN
C2orf88	-1.34733	3.409664	-5.61053	1.22E-05	0.001497	3.320991	DOWN
C3orf33	-1.21208	-0.29207	-3.35785	0.002843	0.036932	-1.5846	DOWN
C4A	1.366749	1.743782	2.857782	0.009149	0.076613	-2.84789	UP
C4BPB	2.603341	-0.59855	7.179344	3.39E-07	0.000281	6.263697	UP
C5orf46	2.09293	1.54831	2.879436	0.008707	0.074551	-2.77512	UP
C6	-1.47973	3.852993	-3.70451	0.001237	0.021987	-1.19869	DOWN
CA12	2.018843	3.398005	6.28941	2.49E-06	0.000729	4.85969	UP
CA3	-1.50929	5.35738	-3.56889	0.001715	0.027138	-1.63525	DOWN
CABP7	-1.42479	-1.10468	-2.21581	0.037358	0.179563	-3.75025	DOWN
CACNA1H	2.80559	2.191324	5.075926	4.38E-05	0.00294	2.156583	UP
CADM1	1.449976	2.593501	6.364961	2.10E-06	0.000655	5.050413	UP
CADM2	-1.96213	1.830318	-5.68455	1.02E-05	0.001324	3.548628	DOWN
CADPS	1.336004	1.587108	3.743591	0.001125	0.020717	-0.88451	UP
CAMK4	1.264645	-1.22514	3.103555	0.005182	0.054016	-2.07985	UP
CAPN6	-1.95725	-1.07802	-3.5131	0.001962	0.029375	-1.24182	DOWN
CARMIL2	1.499671	0.870549	4.187276	0.000382	0.01063	0.177452	UP
CASKIN1	-1.33274	-0.75718	-2.86159	0.009069	0.076428	-2.57616	DOWN
CASQ1	-1.29551	-0.8399	-2.83911	0.009547	0.078805	-2.61591	DOWN
CATSPER1	1.307955	0.020764	3.346029	0.002924	0.037754	-1.63458	UP
CBLN1	-2.40558	0.901959	-5.32372	2.41E-05	0.0021	2.725085	DOWN
CBS	-1.68323	0.501416	-2.84524	0.009414	0.078016	-2.70597	DOWN
CCBE1	-1.42178	0.798803	-3.97082	0.000648	0.01485	-0.29863	DOWN
CCDC102B	1.262853	2.862005	3.945712	0.000688	0.015424	-0.55273	UP
CCDC148	-1.26136	0.116593	-4.4189	0.000217	0.007597	0.712142	DOWN
CCDC160	-1.28008	-1.44475	-3.10689	0.005142	0.053892	-2.065	DOWN
CCDC81	1.695718	0.404964	3.787429	0.001012	0.019281	-0.69507	UP
CCIN	1.457373	-0.2737	3.797747	0.000987	0.018976	-0.64798	UP

CCL11	2.758153	-0.29198	4.391675	0.000232	0.007924	0.646024	UP
CCL19	1.232437	5.535602	2.924532	0.00785	0.07002	-3.10713	UP
CCL26	1.723175	1.406011	3.304752	0.003226	0.03999	-1.84525	UP
CCR7	1.645068	1.090918	4.503053	0.000177	0.00672	0.885549	UP
CD2	1.602553	2.609849	5.998085	4.90E-06	0.000905	4.235737	UP
CD22	1.74688	1.04074	5.089918	4.24E-05	0.002892	2.215942	UP
CD247	1.234326	2.427378	4.796315	8.64E-05	0.004386	1.48142	UP
CD300E	1.583658	0.752409	2.76211	0.011371	0.087105	-2.91961	UP
CD3D	1.56107	2.89981	4.916705	6.45E-05	0.003601	1.72928	UP
CD3E	1.422725	3.43054	5.908677	6.03E-06	0.001008	3.995034	UP
CD3G	1.441238	1.035131	4.317115	0.000278	0.00887	0.46365	UP
CD40LG	1.57556	-0.78606	3.722064	0.001185	0.021432	-0.8042	UP
CD5	1.311283	1.836401	5.101075	4.13E-05	0.002868	2.223281	UP
CD52	1.54274	5.655687	4.01861	0.000576	0.013896	-0.59908	UP
CD70	1.650321	-0.91628	4.471611	0.000191	0.007068	0.794106	UP
CD72	1.477828	2.861815	4.589911	0.000143	0.005799	0.961201	UP
CD79A	3.61694	1.005932	3.997801	0.000606	0.01431	-0.24556	UP
CD96	1.599199	-0.20561	4.782317	8.94E-05	0.004431	1.502703	UP
CDCP1	1.512373	2.764339	4.360383	0.00025	0.008279	0.429776	UP
CDH20	-1.79294	-1.10443	-5.08102	4.33E-05	0.002925	2.043293	DOWN
CDH6	2.027285	1.58575	4.6184	0.000133	0.00554	1.127588	UP
CDKL2	-1.94517	-0.49797	-5.46089	1.74E-05	0.001781	2.897282	DOWN
CDKN2A	1.960273	2.626197	8.225126	3.71E-08	6.43E-05	8.89383	UP
CDKN2B	1.821604	2.88528	9.664124	2.24E-09	1.42E-05	11.5589	UP
CEMIP	1.589683	5.388284	5.023758	4.97E-05	0.003096	1.814118	UP
CFD	-1.2565	6.4345	-3.19236	0.004207	0.047542	-2.53465	DOWN
CGREF1	1.335641	3.347674	4.820062	8.15E-05	0.004205	1.461061	UP
CHAD	-1.48269	5.404489	-3.22571	0.003889	0.045352	-2.42342	DOWN
CHADL	-1.24031	2.770543	-3.99274	0.000614	0.014416	-0.4173	DOWN
CHI3L1	2.389942	5.132514	3.433826	0.002372	0.033333	-1.92986	UP
CHIT1	1.639545	2.550309	2.561712	0.017789	0.114674	-3.56346	UP
CHODL	-3.18598	0.606467	-5.50758	1.56E-05	0.001683	3.092454	DOWN
CHRDL1	-1.63768	3.740796	-3.00132	0.006575	0.062134	-2.76755	DOWN
CHRDL2	2.19384	2.717963	4.151389	0.000417	0.011147	-0.0476	UP
CHRM2	-1.21019	-1.01261	-2.13491	0.044155	0.197797	-3.89428	DOWN
CHST7	-1.33472	4.212146	-4.6408	0.000126	0.005351	0.978703	DOWN
CHST9	-1.92241	0.731636	-4.01251	0.000585	0.014032	-0.19871	DOWN
CIDEA	-2.99618	0.212122	-3.11616	0.005031	0.053259	-2.10699	DOWN
CIDEC	-3.45798	1.67186	-2.90316	0.008245	0.072263	-2.66265	DOWN
CKMT2	-1.67408	2.6874	-3.3867	0.002654	0.035421	-1.78379	DOWN
CLDN10	-1.52746	2.273385	-4.76196	9.39E-05	0.00446	1.4261	DOWN
CLDN14	2.276887	-0.94189	5.366436	2.18E-05	0.001964	2.674558	UP
CLEC3B	-1.58281	9.520416	-5.98275	5.07E-06	0.00092	4.09326	DOWN
CLEC4A	1.474515	-0.5149	3.539858	0.001839	0.028258	-1.19641	UP

CMA1	1.71349	0.368521	3.088245	0.005371	0.055434	-2.20621	UP
CNNM1	-1.45055	-0.22532	-5.27365	2.72E-05	0.002293	2.544476	DOWN
CNTFR	-2.88829	3.934887	-6.44142	1.76E-06	0.000625	5.199706	DOWN
CNTN1	-1.72288	2.668278	-3.67008	0.001344	0.023181	-1.13742	DOWN
CNTNAP2	-2.43598	0.090833	-2.93954	0.007584	0.068508	-2.4659	DOWN
COBL	-1.32089	3.057901	-3.61744	0.001526	0.025053	-1.31884	DOWN
COCH	-2.75007	2.516479	-4.57166	0.000149	0.005921	0.989928	DOWN
COL10A1	5.121811	0.988898	7.320052	2.50E-07	0.000216	6.801242	UP
COL11A1	4.661398	2.410303	8.040327	5.43E-08	8.62E-05	8.435028	UP
COL1A1	1.515392	12.29429	3.757396	0.001088	0.020355	-1.06929	UP
COL28A1	-1.28483	0.142939	-3.86778	0.000832	0.017188	-0.5019	DOWN
COL2A1	1.865373	-0.02291	2.476637	0.021432	0.129686	-3.38708	UP
COL3A1	1.425848	11.28658	4.495232	0.00018	0.006813	0.640161	UP
COL4A1	1.345355	9.054813	4.951003	5.93E-05	0.003429	1.65987	UP
COL4A2	1.243002	9.636289	4.7195	0.000104	0.004861	1.12394	UP
COL4A3	-1.91664	4.114094	-4.81702	8.21E-05	0.004216	1.417161	DOWN
COL4A4	-1.45802	4.571496	-4.6994	0.000109	0.004918	1.092329	DOWN
COL5A1	1.219613	9.214988	4.414944	0.000219	0.007623	0.382828	UP
COL5A2	1.224057	7.923034	4.711201	0.000106	0.00488	1.056061	UP
COL6A6	-1.94068	5.662938	-6.62073	1.17E-06	0.000541	5.523439	DOWN
COL8A1	1.291567	7.041224	6.42267	1.84E-06	0.000625	5.057203	UP
COL9A1	-2.43416	1.55415	-4.91537	6.47E-05	0.003602	1.819829	DOWN
COMP	1.208977	10.70697	5.036569	4.82E-05	0.003061	1.910102	UP
CPAMD8	-1.33544	7.134081	-3.68661	0.001291	0.022539	-1.39835	DOWN
CPNE5	-1.22046	3.011314	-3.34442	0.002936	0.037822	-1.93039	DOWN
CPNE7	1.98072	-1.07003	4.767998	9.25E-05	0.004445	1.413411	UP
CPZ	2.659223	-0.95008	5.472193	1.69E-05	0.001762	2.891434	UP
CR1	1.699227	2.937391	4.383375	0.000237	0.007986	0.470612	UP
CRABP2	2.069492	3.928229	7.099829	4.04E-07	0.000307	6.619331	UP
CRHBP	2.591114	3.652544	6.107377	3.80E-06	0.000861	4.445595	UP
CRLF1	1.316752	6.083529	5.540552	1.44E-05	0.001602	3.019905	UP
CRTAC1	1.247579	7.715525	3.760231	0.00108	0.020271	-1.21603	UP
CRYM	-2.15663	-1.10837	-4.28942	0.000298	0.009189	0.38684	DOWN
CSDC2	-1.47524	4.014084	-5.40164	2.00E-05	0.001898	2.796001	DOWN
CST2	1.315651	1.42563	2.596073	0.016489	0.109891	-3.33817	UP
CST6	1.557237	3.511339	3.817192	0.000941	0.018598	-0.91713	UP
CTHRC1	1.937881	4.357162	5.138262	3.77E-05	0.002731	2.144469	UP
CTSW	1.783783	2.430658	5.2792	2.69E-05	0.002274	2.606469	UP
CX3CR1	1.471131	3.931751	3.66729	0.001353	0.023296	-1.31073	UP
CXCL14	1.604054	4.898778	3.817151	0.000941	0.018598	-1.03805	UP
CXCL2	-1.69868	4.361074	-2.44966	0.022725	0.134186	-3.9863	DOWN
CXXC4	-1.43182	-0.05199	-3.74021	0.001134	0.020847	-0.77442	DOWN
CYP4B1	-1.68005	1.252592	-2.22323	0.036785	0.177974	-3.99165	DOWN
CYP8B1	-1.32741	0.017809	-5.14075	3.75E-05	0.002731	2.284229	DOWN

DDIT4L	1.732941	1.05223	4.100767	0.000472	0.012074	-0.02776	UP
DERL3	2.718751	2.054047	4.404509	0.000225	0.007735	0.606907	UP
DGAT2	-1.93571	1.058021	-2.85728	0.009159	0.076642	-2.73287	DOWN
DIO2	1.812181	2.826182	5.376505	2.13E-05	0.001954	2.807992	UP
DIPK2B	2.236337	0.687752	3.779795	0.00103	0.019544	-0.7249	UP
DIRAS1	1.77574	2.308721	7.803882	8.89E-08	0.000113	8.048224	UP
DKK2	-1.2738	4.522897	-3.61371	0.00154	0.025191	-1.47498	DOWN
DLL4	1.584012	2.217238	2.831962	0.009704	0.07948	-2.96478	UP
DLX3	1.626723	1.0986	4.784965	8.88E-05	0.004431	1.526579	UP
DLX5	2.405859	-0.37639	4.138232	0.00043	0.011355	0.092912	UP
DNAJC22	1.428987	2.108216	5.813157	7.55E-06	0.001096	3.832092	UP
DNER	-2.22616	-1.00562	-4.13138	0.000438	0.01147	0.057675	DOWN
DOCK10	1.412039	3.331743	8.2517	3.52E-08	6.43E-05	8.986827	UP
DRP2	-1.41333	0.828791	-4.21464	0.000357	0.010168	0.247171	DOWN
DUSP1	-1.41455	8.348884	-2.86119	0.009078	0.076428	-3.23575	DOWN
EBF2	1.44381	0.065767	2.403335	0.025116	0.142758	-3.5389	UP
EDNRA	2.259624	1.665291	5.182726	3.39E-05	0.002622	2.418284	UP
EFHD1	1.368491	2.947995	2.651451	0.014579	0.101698	-3.43873	UP
EFNB3	1.584366	0.462146	5.094505	4.19E-05	0.002892	2.216349	UP
EIF5AL1	-1.56446	3.605086	-4.06675	0.000513	0.01276	-0.31881	DOWN
ELAPOR2	1.296409	3.02817	6.519104	1.48E-06	0.000562	5.379479	UP
EMILIN3	-1.37667	4.090666	-3.90913	0.000753	0.016242	-0.74685	DOWN
ENHO	-1.63161	-0.58108	-4.26896	0.000313	0.009396	0.36727	DOWN
ENPEP	1.892748	0.19098	3.721095	0.001188	0.021442	-0.83005	UP
ENPP1	1.584732	2.564605	5.11323	4.01E-05	0.002819	2.21283	UP
ENPP2	1.285906	4.765171	6.56346	1.33E-06	0.000541	5.409859	UP
ENTPD2	-2.31403	-0.0316	-3.83885	0.000893	0.017946	-0.55874	DOWN
EPB41L4B	-2.3118	1.762318	-6.05375	4.30E-06	0.000875	4.35761	DOWN
EPS8L1	1.773399	1.273782	5.060032	4.56E-05	0.002949	2.146845	UP
ERBB3	-1.60093	3.752351	-3.97763	0.000637	0.014667	-0.54814	DOWN
ESM1	2.713504	0.426468	5.491484	1.62E-05	0.001711	3.076795	UP
EVA1A	1.32008	0.342326	3.248797	0.003683	0.043641	-1.8651	UP
F2RL3	-1.26997	0.239187	-3.00522	0.006515	0.061839	-2.35395	DOWN
FABP4	-1.68031	3.586951	-2.4436	0.023025	0.135213	-3.90566	DOWN
FAM163A	-1.6912	-0.91425	-2.9396	0.007583	0.068508	-2.41367	DOWN
FAM180B	-1.25696	-0.94902	-2.42921	0.023753	0.137359	-3.38521	DOWN
FAM20A	1.220963	5.305487	4.080556	0.000496	0.012483	-0.4421	UP
FAM241B	-1.24005	1.573782	-4.59014	0.000143	0.005799	1.069698	DOWN
FAM83D	2.436073	0.580479	4.293682	0.000294	0.009148	0.430098	UP
FAP	2.189373	2.911622	5.887435	6.34E-06	0.001026	3.976551	UP
FBXO2	-1.60998	3.94641	-4.47136	0.000191	0.007068	0.603626	DOWN
FCGBP	1.88782	6.106457	5.466149	1.72E-05	0.001768	2.849376	UP
FCGR1A	2.344859	3.506133	6.446371	1.74E-06	0.000625	5.208786	UP
FCGR1B	1.811066	1.796125	6.770947	8.37E-07	0.000483	5.906966	UP

FCGR3A	2.10005	6.145713	6.888256	6.44E-07	0.000423	6.104581	UP
FCGR3B	1.846117	-0.06369	3.073369	0.00556	0.056509	-2.20207	UP
FCMR	1.718518	1.902723	5.272052	2.73E-05	0.002293	2.613529	UP
FCN3	2.273858	0.869394	5.40794	1.97E-05	0.001889	2.92041	UP
FGF1	1.335291	3.469233	6.002591	4.84E-06	0.000905	4.205684	UP
FGF7	1.242685	4.179306	5.955397	5.41E-06	0.000954	4.058431	UP
FGFR4	-1.42777	1.893269	-5.04574	4.72E-05	0.003024	2.101039	DOWN
FHAD1	1.397784	-0.09318	2.482645	0.021153	0.128532	-3.37374	UP
FIBCD1	1.503219	-1.14292	3.315017	0.003148	0.039374	-1.65212	UP
FKBP5	-1.51561	7.3302	-2.78279	0.010851	0.085143	-3.4253	DOWN
FLRT1	-2.04279	-0.63672	-3.48617	0.002092	0.030677	-1.30223	DOWN
FLT4	1.842114	1.837323	3.558251	0.00176	0.02756	-1.32519	UP
FMO3	1.280792	2.773484	4.704907	0.000108	0.004904	1.238828	UP
FN1	1.463902	12.6736	5.886226	6.36E-06	0.001026	3.939653	UP
FNDC1	1.760818	6.538418	9.231267	5.07E-09	2.33E-05	10.8836	UP
FOLR1	-1.58506	0.044574	-3.85308	0.000862	0.017671	-0.53104	DOWN
FOS	-1.89694	8.169097	-2.51842	0.019563	0.121906	-3.95283	DOWN
FOXS1	1.237305	4.431156	4.109084	0.000462	0.011928	-0.31997	UP
FPR1	1.291428	4.373247	3.426123	0.002416	0.033704	-1.91208	UP
FRRS1L	-1.48398	0.148786	-2.5997	0.016356	0.109559	-3.15758	DOWN
FXYD3	-1.44996	-1.53447	-3.73877	0.001138	0.02089	-0.77852	DOWN
GADD45G	-1.41264	3.601256	-2.53315	0.018942	0.119478	-3.73776	DOWN
GALNT3	2.066903	0.1674	6.235862	2.82E-06	0.000779	4.618751	UP
GAP43	2.107836	4.012001	6.428698	1.81E-06	0.000625	5.147099	UP
GBP5	1.334498	2.238692	4.088947	0.000486	0.012312	-0.15348	UP
GFRA3	-1.61054	0.324382	-2.18039	0.040208	0.187324	-3.95573	DOWN
GIPC3	2.166042	1.300724	6.569776	1.32E-06	0.000541	5.447331	UP
GIPR	1.629316	-0.55917	5.106338	4.07E-05	0.002852	2.17266	UP
GPBAR1	-1.42813	3.667044	-5.80342	7.72E-06	0.001106	3.749782	DOWN
GPC5	-2.01608	-1.1714	-4.18677	0.000382	0.01063	0.16857	DOWN
GPD1	-2.74959	3.107862	-2.76127	0.011393	0.087197	-3.15823	DOWN
GPM6A	-2.51569	0.719105	-5.22554	3.06E-05	0.002446	2.498435	DOWN
GPR12	-1.68144	-0.6238	-3.8042	0.000971	0.01889	-0.62923	DOWN
GPR37L1	-1.36443	1.844974	-4.37993	0.000239	0.008025	0.56918	DOWN
GPR68	2.087907	3.116832	6.610276	1.20E-06	0.000541	5.57952	UP
GPR84	1.816982	0.944266	4.933045	6.20E-05	0.003502	1.863263	UP
GPR85	1.432391	0.563698	5.112716	4.01E-05	0.002819	2.260257	UP
GPR88	1.69668	1.547091	4.774067	9.12E-05	0.004431	1.486848	UP
GREB1L	-2.40695	-0.86169	-4.9233	6.35E-05	0.003564	1.735746	DOWN
GREM1	1.982789	0.229747	3.105916	0.005153	0.053921	-2.15412	UP
GRIK3	-2.40609	2.233044	-4.28747	0.000299	0.009198	0.343061	DOWN
GRM7	1.66525	-0.63025	3.736278	0.001145	0.020946	-0.77493	UP
GUCY1A2	2.069184	1.066667	4.602098	0.000139	0.005712	1.112135	UP
GZMA	1.384642	2.084548	4.807043	8.42E-05	0.004296	1.532032	UP

GZMB	1.75423	1.005949	3.82971	0.000913	0.018279	-0.63781	UP
GZMK	1.205814	0.992896	4.256589	0.000322	0.009608	0.327903	UP
HAMP	2.039036	-0.14588	3.324825	0.003075	0.038811	-1.66843	UP
HAP1	-1.41352	-0.73246	-3.17492	0.004383	0.048759	-1.94697	DOWN
HAPLN3	1.270206	5.656863	4.063801	0.000516	0.012804	-0.49488	UP
HCN1	-1.23604	-1.42694	-3.07128	0.005588	0.056564	-2.13615	DOWN
HEY1	1.218991	1.449437	4.052564	0.000531	0.01304	-0.16756	UP
HHIP	1.430989	-0.02314	4.166302	0.000402	0.010904	0.15519	UP
HIF3A	-2.07339	4.002568	-5.31804	2.45E-05	0.002109	2.614371	DOWN
HIGD1B	1.553613	-0.85163	2.798171	0.010479	0.083521	-2.70266	UP
HLA-DOB	1.495536	-0.02691	3.338386	0.002978	0.038162	-1.64757	UP
HLA-DQA1	1.254738	6.368967	2.374661	0.02671	0.147821	-4.26382	UP
HLA-DQB1	1.476352	6.398054	2.715412	0.012634	0.092854	-3.5719	UP
HLA-DRA	1.234234	9.672594	6.536527	1.42E-06	0.000562	5.35616	UP
HLA-DRB1	1.514586	8.234112	4.42379	0.000214	0.007542	0.376353	UP
HMGCLL1	-1.39087	1.489063	-4.79323	8.70E-05	0.004396	1.538383	DOWN
HMOX1	1.254176	6.468555	2.39067	0.025809	0.144678	-4.23336	UP
HMSD	-1.37817	0.282161	-4.23347	0.000341	0.009855	0.302613	DOWN
HOPX	1.32093	3.672871	5.873881	6.55E-06	0.001028	3.899367	UP
HOXD8	2.060491	-1.31661	4.830472	7.95E-05	0.004193	1.524407	UP
HOXD9	2.278636	-0.8306	3.114986	0.005045	0.053317	-2.07167	UP
HPR	-1.58887	1.671573	-3.25167	0.003658	0.043507	-1.96529	DOWN
HSD17B13	-1.70655	0.326151	-3.79781	0.000986	0.018976	-0.66012	DOWN
HSD17B6	1.386231	0.169323	3.679765	0.001313	0.022817	-0.92088	UP
HSH2D	1.584732	0.35588	3.547663	0.001805	0.028017	-1.21962	UP
HTRA4	1.840881	-0.66853	3.56885	0.001716	0.027138	-1.13109	UP
IBSP	7.077498	1.928002	12.03032	3.79E-11	4.16E-07	14.12895	UP
ICAM3	1.346215	0.995024	3.662811	0.001368	0.023401	-1.01296	UP
ICAM5	1.286498	1.468423	4.109059	0.000462	0.011928	-0.03922	UP
IDO1	1.529904	-0.71078	3.003419	0.006543	0.061955	-2.30297	UP
IFITM10	1.582104	5.731433	3.927914	0.000719	0.015791	-0.81668	UP
IGFBP1	2.885927	-0.64186	5.173852	3.46E-05	0.002622	2.305483	UP
IGFBP2	1.813453	6.530625	4.343487	0.000261	0.008476	0.16335	UP
IGFN1	-1.87828	2.226748	-5.71523	9.51E-06	0.001271	3.615021	DOWN
IGSF1	-2.22795	-0.38377	-3.59873	0.001596	0.025813	-1.06869	DOWN
IGSF10	-1.82619	4.624767	-4.17257	0.000396	0.010831	-0.15801	DOWN
IGSF11	-1.37636	0.635418	-2.95487	0.00732	0.066891	-2.49531	DOWN
IL1B	1.264646	0.790948	2.283733	0.032403	0.165272	-3.84988	UP
IL1RN	1.340993	0.863749	2.350222	0.028142	0.152376	-3.73766	UP
IL21R	2.98901	0.877835	6.952363	5.59E-07	0.000394	6.160142	UP
IL2RB	1.317217	2.671703	4.44226	0.000205	0.007304	0.631637	UP
IL4I1	1.234583	1.928953	2.752802	0.011613	0.088279	-3.09151	UP
IL7R	2.141693	1.713115	6.729759	9.18E-07	0.000489	5.812842	UP
INHBA	1.408657	5.537496	5.366224	2.18E-05	0.001964	2.621541	UP

IP6K3	-2.08802	0.507574	-2.99449	0.006679	0.062845	-2.387	DOWN
IPCEF1	1.256616	0.159127	3.904349	0.000761	0.016288	-0.42674	UP
IRAG1	1.324542	5.475883	3.665538	0.001359	0.02331	-1.42688	UP
ISLR2	1.746824	0.438634	4.580673	0.000146	0.005869	1.073938	UP
ISM1	1.499878	2.283625	3.771459	0.001051	0.019825	-0.8871	UP
ITGAX	1.282168	4.419066	3.07658	0.005519	0.056292	-2.70201	UP
ITIH3	2.162408	2.722397	4.183558	0.000385	0.010683	0.030636	UP
ITK	1.326562	0.549087	5.321562	2.43E-05	0.002101	2.719804	UP
ITLN1	-3.31027	3.372147	-4.41654	0.000218	0.007621	0.578596	DOWN
JAG2	1.662625	2.093627	5.96755	5.26E-06	0.000936	4.17741	UP
JCHAIN	4.398686	2.546075	3.978349	0.000636	0.014664	-0.3798	UP
JPH1	-1.28449	-0.53501	-3.01374	0.006388	0.060945	-2.28553	DOWN
KCNH8	-1.20789	-2.08152	-3.33374	0.003011	0.03846	-1.60935	DOWN
KCNIP2	-1.21446	2.853181	-3.16323	0.004506	0.049652	-2.31842	DOWN
KCNJ15	1.996126	0.812977	5.104806	4.09E-05	0.002853	2.247393	UP
KCNN1	-1.63625	-0.51663	-3.12972	0.004874	0.052284	-2.04797	DOWN
KCNN4	1.460748	3.975737	5.569807	1.34E-05	0.001558	3.179213	UP
KCNQ3	1.370755	0.369681	4.315276	0.000279	0.008883	0.481303	UP
KCTD4	-1.22229	0.027636	-2.72024	0.012497	0.092349	-2.91459	DOWN
KIF1A	-1.60817	2.995565	-4.46246	0.000195	0.0071	0.67122	DOWN
KISS1	-1.74937	2.601234	-3.28448	0.003384	0.041235	-2.00568	DOWN
KLF15	-1.58491	5.102476	-3.20012	0.004131	0.046937	-2.45872	DOWN
KLHDC7B	1.506872	0.402278	3.171871	0.004415	0.048909	-2.03313	UP
KRT14	4.370126	0.940925	8.548913	1.93E-08	4.83E-05	9.057221	UP
KRT16	2.959788	-0.02684	6.121491	3.67E-06	0.000855	4.342152	UP
KRT17	2.289937	3.4918	6.65895	1.08E-06	0.000525	5.677158	UP
KRT18	2.246338	3.351397	6.151788	3.43E-06	0.000847	4.554845	UP
KRT19	-1.37053	1.23808	-3.36689	0.002783	0.036394	-1.67549	DOWN
KRT7	2.122083	0.958725	3.933272	0.00071	0.01572	-0.39969	UP
LAIR2	1.333762	-1.0929	3.351144	0.002889	0.037373	-1.57852	UP
LAMC3	-1.26011	6.60298	-4.04838	0.000536	0.013121	-0.54572	DOWN
LAMP3	1.625286	-1.47853	3.341666	0.002955	0.037942	-1.59338	UP
LAMP5	1.743416	3.398928	5.261711	2.80E-05	0.00231	2.503221	UP
LCK	1.230432	3.140308	3.972043	0.000646	0.01485	-0.52056	UP
LCN12	-2.80429	0.380498	-5.34232	2.31E-05	0.002036	2.725662	DOWN
LCNL1	-1.33229	2.989377	-3.94443	0.000691	0.015454	-0.55119	DOWN
LEFTY2	-1.47347	0.712361	-3.6007	0.001589	0.025772	-1.11556	DOWN
LGALS12	-1.91263	1.206215	-3.04408	0.005953	0.058609	-2.3597	DOWN
LHX6	3.336676	0.028368	6.693819	9.95E-07	0.000512	5.482651	UP
LIPE	-1.47646	3.319691	-2.89824	0.008339	0.072548	-2.94756	DOWN
LONRF2	-1.27226	1.916062	-5.26438	2.78E-05	0.002307	2.59895	DOWN
LOXL2	1.687346	5.317958	4.71488	0.000105	0.004868	1.07898	UP
LPL	-1.40489	4.37324	-2.63048	0.015276	0.104974	-3.63147	DOWN
LRP1B	-2.05239	-0.47886	-4.70931	0.000107	0.00488	1.315938	DOWN

LRRC15	2.732178	3.428115	7.888563	7.45E-08	0.000101	8.261852	UP
LRRIQ1	-1.48475	-1.02821	-3.14476	0.004705	0.051172	-1.99857	DOWN
LSP1	1.218469	7.256851	4.461614	0.000195	0.0071	0.444796	UP
LTB	1.417643	3.94378	3.581258	0.001665	0.0266	-1.51307	UP
LTF	1.301182	0.621673	2.664229	0.014169	0.099678	-3.10139	UP
LUM	1.582894	7.370324	6.737217	9.02E-07	0.000489	5.767143	UP
LY6H	1.684465	-0.29053	2.380598	0.026372	0.14663	-3.54107	UP
LY9	1.634412	1.026402	5.06506	4.50E-05	0.002942	2.16008	UP
LYPD6	-2.85038	-0.31669	-6.03439	4.50E-06	0.000892	4.074019	DOWN
LYZ	1.204313	6.995456	2.924982	0.007842	0.06998	-3.12689	UP
MACC1	1.234347	-1.22613	2.703652	0.012972	0.094321	-2.86265	UP
MAP2K6	-1.22314	2.70557	-4.13914	0.00043	0.011345	-0.06798	DOWN
MAP3K7CL	1.206169	3.865354	6.006787	4.80E-06	0.000905	4.192404	UP
MAPK4	-1.44144	2.500078	-3.60194	0.001584	0.025722	-1.28422	DOWN
MAPT	-1.47357	5.137857	-5.44301	1.82E-05	0.001816	2.826484	DOWN
MARCHF4	1.579534	-0.84927	3.209802	0.004038	0.046265	-1.8762	UP
MATK	1.262311	1.923188	3.34992	0.002897	0.037457	-1.80606	UP
MATN3	1.377462	0.213416	3.469866	0.002176	0.031461	-1.37985	UP
MFAP5	2.477566	2.748261	6.563231	1.34E-06	0.000541	5.483721	UP
MGST1	-1.27861	4.833797	-4.34926	0.000257	0.00843	0.235834	DOWN
MKI67	1.25446	2.705913	3.147343	0.004677	0.050893	-2.35336	UP
MLXIPL	-2.38861	0.142874	-3.07961	0.00548	0.056047	-2.18293	DOWN
MMP1	3.138406	-0.17601	3.751732	0.001103	0.020431	-0.74732	UP
MMP11	2.20495	2.6754	8.967111	8.44E-09	2.68E-05	10.28616	UP
MMP7	3.706627	0.636116	3.988216	0.000621	0.014522	-0.2499	UP
MMP9	4.123774	4.241805	5.774223	8.27E-06	0.00115	3.683543	UP
MNDA	1.251967	3.364636	5.337941	2.33E-05	0.002048	2.677842	UP
MOXD1	1.57605	1.765701	6.933092	5.83E-07	0.000397	6.243895	UP
MRAP	-2.24449	0.488882	-3.56829	0.001718	0.027148	-1.16424	DOWN
MT1A	-1.3751	2.868522	-2.90174	0.008273	0.072263	-2.88129	DOWN
MT1G	1.632998	0.190776	2.07884	0.0495	0.209875	-4.13288	UP
MTUS2	-1.37896	1.423795	-3.09623	0.005271	0.054676	-2.279	DOWN
MXRA5	1.632203	7.973202	7.528615	1.59E-07	0.000152	7.490132	UP
MYH11	1.476394	8.21854	2.211566	0.03769	0.180559	-4.54507	UP
MYH14	-1.30456	4.801181	-4.45993	0.000196	0.007116	0.502845	DOWN
MYH7	-4.14705	0.718796	-2.07622	0.049763	0.210354	-4.10632	DOWN
MYLK3	-3.06011	0.112855	-4.76316	9.36E-05	0.004458	1.454783	DOWN
MYO10	1.333833	4.18482	6.058456	4.25E-06	0.000875	4.294311	UP
MYO1G	1.450228	3.649535	3.717964	0.001197	0.021523	-1.16693	UP
MYOC	-2.12608	5.413762	-4.79052	8.76E-05	0.004413	1.274567	DOWN
MYOZ1	1.332054	0.757271	4.252949	0.000325	0.00963	0.330348	UP
MZB1	3.497103	-0.00596	3.807234	0.000964	0.018829	-0.63079	UP
N4BP3	2.064923	0.255679	4.246017	0.000331	0.009693	0.329193	UP
NAALAD2	-1.44262	1.127184	-4.2834	0.000302	0.009215	0.390211	DOWN

NAT8L	-1.91273	1.462897	-3.76281	0.001074	0.020185	-0.80777	DOWN
NCAM1	-1.54003	3.487075	-4.57106	0.00015	0.005921	0.877982	DOWN
NDUFA4L2	1.854546	5.946024	4.081646	0.000494	0.012467	-0.45384	UP
NELL2	3.492474	-0.06084	8.465034	2.28E-08	4.83E-05	8.631235	UP
NKAIN4	-1.47704	-1.62508	-3.7356	0.001147	0.02096	-0.78718	DOWN
NKD1	1.477141	2.858146	4.934868	6.17E-05	0.003497	1.773164	UP
NNAT	-1.51085	1.304429	-3.21074	0.004029	0.046191	-2.02002	DOWN
NOTCH3	1.265148	7.26418	4.936721	6.14E-05	0.003492	1.584323	UP
NPY	-4.37925	-0.3247	-6.22575	2.89E-06	0.00078	4.408025	DOWN
NR4A1	-2.14403	6.596482	-3.40877	0.002518	0.034648	-2.03359	DOWN
NR5A2	1.471401	-1.38439	2.372536	0.026832	0.148189	-3.46549	UP
NRARP	1.511757	0.923686	3.418362	0.002461	0.034128	-1.54764	UP
NSG1	-1.69059	6.312623	-6.07299	4.11E-06	0.000875	4.264564	DOWN
NT5C1A	-1.73997	0.329519	-4.12055	0.000449	0.011666	0.052813	DOWN
NTM	1.496215	5.581334	5.178108	3.43E-05	0.002622	2.174648	UP
NUDT10	-1.61092	1.678039	-3.87856	0.000811	0.017002	-0.56599	DOWN
NXPH4	2.840042	-0.25809	6.039336	4.45E-06	0.000891	4.142755	UP
OLR1	1.952116	0.618403	3.457733	0.00224	0.032051	-1.43428	UP
OXTR	2.353489	0.2656	3.545051	0.001817	0.028056	-1.2175	UP
P2RY10	1.490867	-1.692	4.382031	0.000237	0.007998	0.558349	UP
P4HA3	1.314739	2.527653	3.766518	0.001064	0.020044	-0.93109	UP
PACRG	-1.45091	0.391277	-3.12707	0.004904	0.052528	-2.11259	DOWN
PAX7	-1.6649	3.986122	-4.39818	0.000228	0.007827	0.427709	DOWN
PCDH1	1.60897	4.742755	5.881362	6.43E-06	0.001028	3.860758	UP
PCSK9	2.647466	-1.01681	5.133624	3.81E-05	0.002731	2.186561	UP
PDK4	-2.52455	5.462299	-3.83858	0.000893	0.017946	-0.9823	DOWN
PHEX	-1.49631	0.662515	-4.31305	0.000281	0.008883	0.474275	DOWN
PIM2	1.421195	4.251887	3.850643	0.000868	0.017671	-0.91429	UP
PKHD1L1	-1.45984	4.873964	-4.27822	0.000306	0.009301	0.068105	DOWN
PKP2	-1.82574	3.886283	-4.4134	0.00022	0.007638	0.478292	DOWN
PLA2G2D	1.88223	-0.3359	2.746079	0.011791	0.089027	-2.84085	UP
PLAU	1.89032	5.226725	5.497939	1.59E-05	0.001697	2.948063	UP
PLCH1	-1.79563	3.101223	-5.50633	1.56E-05	0.001683	3.105133	DOWN
PLEK2	1.397379	1.473966	4.556361	0.000155	0.006086	0.988353	UP
PLEKHN1	1.654688	0.756391	5.416489	1.93E-05	0.001871	2.936805	UP
PLEKHS1	4.463769	0.528553	7.689319	1.13E-07	0.00012	7.414817	UP
PLIN1	-2.40507	2.781287	-2.6702	0.013981	0.09897	-3.32135	DOWN
PLIN4	-2.20495	4.788838	-2.33449	0.029101	0.155206	-4.23042	DOWN
PLIN5	-2.47685	-0.50385	-3.3274	0.003057	0.038707	-1.63672	DOWN
PLLP	-1.54941	3.887105	-4.46243	0.000195	0.0071	0.585879	DOWN
PLPP4	1.930348	-0.49408	4.641183	0.000126	0.005351	1.181461	UP
PLPPR4	1.359255	2.324819	6.009456	4.77E-06	0.000905	4.267151	UP
PLXNA4	-1.2929	5.71186	-5.25588	2.84E-05	0.002333	2.358728	DOWN
PNMA6A	-1.36667	-0.67613	-4.25233	0.000326	0.00963	0.32966	DOWN

PNMT	-3.3846	0.950198	-6.13893	3.53E-06	0.000851	4.448339	DOWN
POU2AF1	1.880355	0.698138	3.018329	0.00632	0.060725	-2.38513	UP
POU5F1B	-1.6887	-1.32321	-4.17918	0.00039	0.010693	0.146314	DOWN
PPBP	2.107881	-0.17672	3.125732	0.00492	0.052602	-2.0843	UP
PPP1R1A	-2.96637	0.984184	-3.23433	0.003811	0.044657	-1.91326	DOWN
PPP1R1B	-1.98124	5.680959	-5.29411	2.59E-05	0.002214	2.461862	DOWN
PPP1R9A	-1.5989	0.24837	-4.65554	0.000122	0.005255	1.235601	DOWN
PRCD	-1.22431	3.199178	-5.83467	7.18E-06	0.001059	3.845112	DOWN
PRG4	2.45019	7.128514	3.936178	0.000705	0.015676	-0.79872	UP
PRLR	-1.59824	1.693266	-5.94298	5.57E-06	0.000973	4.117284	DOWN
PRR33	1.390465	3.643336	5.140825	3.75E-05	0.002731	2.195066	UP
PRSS3	2.135058	-0.16611	3.866857	0.000834	0.017208	-0.49948	UP
PRSS35	2.543169	2.277812	5.555757	1.39E-05	0.001579	3.25076	UP
PTCRA	1.426846	-1.40945	3.355042	0.002862	0.037078	-1.56654	UP
PTGDS	-1.79139	7.939124	-4.97477	5.60E-05	0.003347	1.687731	DOWN
PTPN7	1.265928	2.500304	5.412874	1.95E-05	0.001876	2.907556	UP
PWWP3B	-3.78268	1.124141	-4.06402	0.000516	0.012804	-0.07919	DOWN
PYHIN1	1.90698	-0.53367	6.164263	3.33E-06	0.00084	4.350065	UP
RAB27B	1.229791	-2.04728	2.742949	0.011874	0.089404	-2.75913	UP
RAPSN	-1.41365	0.838769	-4.64847	0.000124	0.005299	1.224891	DOWN
RASL10B	-1.38897	0.47953	-3.23459	0.003808	0.044657	-1.89336	DOWN
RBM11	-1.36209	-1.2191	-3.97916	0.000635	0.014664	-0.26838	DOWN
RBP4	-1.36715	3.579693	-3.55196	0.001787	0.027861	-1.52583	DOWN
RELN	-2.45106	0.863621	-4.55885	0.000154	0.006062	1.024773	DOWN
RET	1.297302	-0.44958	3.615423	0.001533	0.025109	-1.03698	UP
RFX8	1.542518	0.526669	3.872592	0.000823	0.017137	-0.51201	UP
RGR	-1.69002	-0.26993	-3.82171	0.000931	0.018502	-0.59334	DOWN
RGS16	1.304932	3.024011	4.07533	0.000502	0.012594	-0.26608	UP
RGS4	1.998392	3.813828	5.381927	2.10E-05	0.001942	2.75596	UP
RGS5	1.618226	4.820513	2.649466	0.014644	0.102037	-3.64185	UP
RIMS4	-1.51204	3.899122	-5.76734	8.41E-06	0.00116	3.654734	DOWN
RNASE2	1.932276	0.829117	4.269214	0.000313	0.009396	0.365542	UP
RPRML	1.698676	0.711047	3.446877	0.002299	0.0325	-1.46541	UP
RRM2	1.385306	1.478874	2.950266	0.007398	0.067282	-2.61936	UP
RSPO4	1.304074	0.003336	2.924264	0.007855	0.07003	-2.51461	UP
RUBCNL	1.45871	3.032664	4.321802	0.000275	0.008799	0.313213	UP
RUNX2	1.306862	3.08125	6.320314	2.32E-06	0.000702	4.938472	UP
S100A1	-1.32492	2.321853	-2.97703	0.006955	0.064796	-2.65299	DOWN
S100A12	1.782768	-0.37558	2.83238	0.009695	0.079446	-2.66833	UP
S100A8	1.694088	3.409812	2.858895	0.009125	0.076519	-3.06426	UP
S100A9	1.755579	6.366621	3.015533	0.006361	0.060905	-2.92419	UP
S1PR5	2.246406	-0.8859	4.774081	9.12E-05	0.004431	1.44073	UP
SAMD12	-1.27581	0.463451	-4.67941	0.000115	0.005038	1.293469	DOWN
SAMD3	1.787418	-0.38188	4.717048	0.000105	0.004866	1.350998	UP

SAMSN1	1.284857	1.130047	4.249894	0.000328	0.009631	0.305259	UP
SCG2	2.185433	5.724472	6.668245	1.05E-06	0.000525	5.623754	UP
SCGN	-2.16691	0.250675	-2.79804	0.010482	0.083521	-2.76984	DOWN
SCIN	2.112712	-0.26724	5.629654	1.16E-05	0.001441	3.303128	UP
SCML4	1.501817	0.148851	4.845206	7.67E-05	0.004068	1.655748	UP
SDC1	1.932699	2.622332	4.3426	0.000261	0.008476	0.405844	UP
SDS	1.855066	0.808125	2.902211	0.008264	0.072263	-2.63785	UP
SELE	2.365576	1.523241	3.170786	0.004426	0.049005	-2.14375	UP
SEMA3G	-1.42675	4.341841	-3.85271	0.000863	0.017671	-0.90307	DOWN
SEMA5B	2.359216	1.056777	4.437487	0.000207	0.007376	0.738531	UP
SEMA6B	1.642886	3.303759	3.62191	0.00151	0.024912	-1.35085	UP
SERPINA1	1.301782	4.591317	3.952558	0.000677	0.015223	-0.70209	UP
SEZ6L2	1.912466	2.498199	5.565674	1.36E-05	0.001564	3.260747	UP
SFRP2	1.339329	7.408884	5.655377	1.10E-05	0.0014	3.291336	UP
SH2D1A	1.674522	-0.0734	3.866113	0.000836	0.017221	-0.50333	UP
SH3RF2	-1.72965	3.958425	-5.13447	3.81E-05	0.002731	2.176598	DOWN
SHISA6	-1.52001	-0.63874	-3.30054	0.003258	0.040182	-1.69077	DOWN
SIGLEC15	1.326315	0.73399	4.144695	0.000424	0.011239	0.086874	UP
SKAP1	1.224999	1.078767	4.768277	9.25E-05	0.004445	1.488439	UP
SLAMF1	1.651121	0.596908	6.292982	2.47E-06	0.000729	4.799369	UP
SLAMF7	2.427563	1.398021	5.865101	6.68E-06	0.001028	3.941928	UP
SLAMF8	1.229375	3.522265	3.401509	0.002562	0.034934	-1.88382	UP
SLC16A12	-1.76504	2.088079	-4.62797	0.00013	0.005468	1.130147	DOWN
SLC16A3	1.45389	5.118218	3.998128	0.000606	0.01431	-0.62635	UP
SLC16A9	-2.00974	2.703814	-7.77167	9.52E-08	0.000113	7.992076	DOWN
SLC19A2	-1.47857	2.42026	-3.62989	0.001481	0.0246	-1.20701	DOWN
SLC19A3	-1.42744	0.886621	-2.56026	0.017846	0.114965	-3.31683	DOWN
SLC1A7	-1.60909	2.408537	-3.87688	0.000814	0.017053	-0.64451	DOWN
SLC25A18	-1.53702	-0.47439	-3.12204	0.004963	0.052902	-2.06545	DOWN
SLC2A5	2.771646	2.242348	4.976034	5.58E-05	0.003347	1.922274	UP
SLC6A4	-1.68082	4.771183	-4.13764	0.000431	0.011355	-0.25559	DOWN
SLC6A6	1.645541	6.040736	7.696122	1.12E-07	0.00012	7.837719	UP
SLC7A4	-2.1135	0.542624	-4.56698	0.000151	0.005955	1.042908	DOWN
SLITRK3	-1.23311	-1.18484	-2.66537	0.014133	0.099521	-2.93305	DOWN
SLITRK5	-2.15383	0.817545	-6.81821	7.53E-07	0.000448	5.860436	DOWN
SLITRK6	1.463568	-1.53078	2.882614	0.008643	0.07433	-2.50782	UP
SLPI	2.465639	3.4315	6.426735	1.82E-06	0.000625	5.168304	UP
SMKR1	1.200583	-0.70488	3.172609	0.004407	0.048853	-1.95857	UP
SMOC2	1.608168	5.138248	4.035584	0.000553	0.013468	-0.53424	UP
SMPDL3B	2.102519	-0.16919	5.469144	1.71E-05	0.001765	2.978258	UP
SMTNL2	-1.83898	2.560537	-4.03127	0.000559	0.013575	-0.29146	DOWN
SORCS1	-2.60595	1.501623	-5.78375	8.09E-06	0.001141	3.754789	DOWN
SOST	-1.37622	4.046191	-2.36136	0.027481	0.150508	-4.12897	DOWN
SOX10	-2.09583	2.674313	-3.51536	0.001951	0.029322	-1.48729	DOWN

SOX2	-2.46385	-1.37316	-5.71332	9.55E-06	0.001271	3.275916	DOWN
SP140	1.392317	1.464463	5.921461	5.86E-06	0.000996	4.07065	UP
SP5	-1.57645	-0.85662	-3.8254	0.000922	0.018367	-0.58545	DOWN
SPOCD1	2.149255	1.154312	4.467417	0.000193	0.0071	0.802321	UP
SPP1	4.609677	8.180169	4.998317	5.29E-05	0.003225	1.775766	UP
SSTR1	-1.63461	1.140111	-4.32929	0.00027	0.008654	0.496045	DOWN
STAR	-1.56019	-0.84771	-4.51495	0.000172	0.006571	0.881003	DOWN
STAT4	1.319317	0.635627	5.025814	4.95E-05	0.00309	2.068937	UP
STMN2	4.849015	1.831045	11.94178	4.37E-11	4.16E-07	14.415	UP
STXBP6	-1.82301	-0.31929	-5.38856	2.07E-05	0.001939	2.769278	DOWN
SUGCT	1.940901	2.765095	6.857362	6.90E-07	0.000424	6.117343	UP
SULF1	1.551223	8.464386	6.138683	3.53E-06	0.000851	4.432008	UP
SULT1A2	-1.69961	2.020182	-6.57006	1.31E-06	0.000541	5.482584	DOWN
SYN2	-1.40232	0.445893	-3.27103	0.003494	0.042059	-1.81369	DOWN
SYT12	1.841953	4.081292	6.528184	1.45E-06	0.000562	5.36411	UP
SYT6	1.282701	-1.39755	3.297275	0.003283	0.040364	-1.6843	UP
SYT8	1.508455	-0.28226	4.866001	7.29E-05	0.003923	1.680415	UP
TAGLN3	-2.47594	0.24496	-3.84607	0.000877	0.01781	-0.54674	DOWN
TBL1Y	-1.83097	-1.6311	-4.68907	0.000112	0.004944	1.184638	DOWN
TBX22	-1.7461	-0.9268	-4.18095	0.000388	0.010693	0.167188	DOWN
TCAP	-2.35774	1.682812	-2.39737	0.02544	0.143386	-3.69471	DOWN
TCEAL6	-1.6127	2.208539	-5.57994	1.31E-05	0.001541	3.309587	DOWN
TCHH	1.479135	2.023653	6.36808	2.08E-06	0.000655	5.057149	UP
TENM4	1.216118	2.717778	5.325842	2.40E-05	0.002099	2.693688	UP
THBS2	1.66569	9.169996	4.728598	0.000102	0.004777	1.133042	UP
THRSP	-2.58412	-0.35292	-2.70769	0.012855	0.093899	-2.8959	DOWN
THSD7B	1.226496	0.738512	3.551674	0.001788	0.027861	-1.23861	UP
THY1	1.962106	6.582673	6.006829	4.80E-06	0.000905	4.109444	UP
TIFAB	1.265672	-1.33068	2.485921	0.021003	0.127862	-3.26338	UP
TIGIT	1.479278	-0.15272	3.884138	0.0008	0.016847	-0.46232	UP
TIMD4	1.623109	2.94022	3.058761	0.005753	0.057453	-2.57201	UP
TLCD4	-1.69956	2.617454	-6.00208	4.85E-06	0.000905	4.250014	DOWN
TLR10	1.860092	-1.44941	4.225972	0.000347	0.009995	0.248389	UP
TLR7	1.22177	2.250843	5.16239	3.56E-05	0.002668	2.343606	UP
TLR8	1.397493	0.801878	4.91347	6.50E-05	0.003608	1.819523	UP
TM4SF18	1.6403	0.436861	3.287545	0.00336	0.041015	-1.7896	UP
TM7SF2	-1.48697	4.412142	-5.38575	2.08E-05	0.001942	2.732645	DOWN
TMEM119	1.299217	4.814852	5.431081	1.87E-05	0.001843	2.803494	UP
TMEM130	1.469811	2.964759	3.910613	0.00075	0.016209	-0.64429	UP
TMEM132C	-1.62759	5.602075	-4.94096	6.08E-05	0.003482	1.618389	DOWN
TMEM132D	-1.44703	-1.00809	-2.86273	0.009046	0.076389	-2.56146	DOWN
TMEM158	1.234882	1.247508	3.841099	0.000888	0.017912	-0.63315	UP
TMEM163	1.374489	0.225147	3.253192	0.003645	0.043378	-1.84641	UP
TMEM200A	2.623598	1.086435	7.945387	6.61E-08	9.69E-05	8.115845	UP

TMEM225	-1.29888	-1.18145	-3.61101	0.00155	0.02529	-1.03833	DOWN
TMEM26	1.698261	-0.81713	3.754912	0.001094	0.020374	-0.73424	UP
TMEM38A	-1.35895	2.451361	-5.9264	5.79E-06	0.000993	4.08236	DOWN
TNC	1.610294	9.243053	3.930635	0.000714	0.01576	-0.76741	UP
TNFAIP6	1.509104	3.115648	7.532122	1.58E-07	0.000152	7.53577	UP
TNFRSF11B	1.372451	6.870284	3.407928	0.002523	0.034667	-2.04687	UP
TNFRSF18	1.601169	-0.8422	3.590442	0.001629	0.02615	-1.0827	UP
TNFRSF4	1.58318	1.623731	3.288404	0.003353	0.040958	-1.90362	UP
TNFSF11	3.041495	-0.47591	6.178924	3.22E-06	0.000828	4.384072	UP
TNN	1.73602	-0.00346	4.09689	0.000476	0.01214	0.002345	UP
TNNC1	-2.39676	2.11364	-2.45566	0.022431	0.133237	-3.64537	DOWN
TPPP	-1.29074	4.88958	-4.96694	5.71E-05	0.003376	1.708626	DOWN
TPSB2	-1.38167	4.184866	-2.09114	0.048279	0.207117	-4.65836	DOWN
TRAF3IP3	1.475596	2.496548	5.605452	1.23E-05	0.001505	3.349714	UP
TRARG1	-2.18324	1.943337	-3.18304	0.0043	0.048173	-2.13679	DOWN
TRDN	-1.89451	1.944142	-3.23649	0.003791	0.04454	-2.0231	DOWN
TREM1	1.989716	3.582647	3.913206	0.000745	0.016187	-0.69682	UP
TREM2	1.657454	4.330031	3.819329	0.000936	0.018588	-0.99252	UP
TRIM63	-2.54184	-0.04064	-3.62684	0.001492	0.024746	-1.01399	DOWN
TRPC4	1.552586	1.002362	4.46674	0.000193	0.0071	0.805753	UP
TSPAN11	1.532911	3.900151	7.320457	2.49E-07	0.000216	7.08795	UP
TSPAN7	-1.24736	4.691671	-5.53089	1.47E-05	0.00163	3.052296	DOWN
TTC34	-1.19986	0.229241	-3.95678	0.00067	0.015139	-0.30921	DOWN
TTYH1	-1.53044	0.758603	-4.21163	0.00036	0.010228	0.243305	DOWN
TUBB3	1.723256	-0.22899	4.145189	0.000423	0.011239	0.109332	UP
U2AF1	-1.76275	0.233521	-2.88099	0.008675	0.074486	-2.59916	DOWN
UBASH3A	1.359027	1.071056	4.250636	0.000327	0.00963	0.310996	UP
ULBP1	1.48714	-0.93709	3.696959	0.001259	0.022267	-0.85708	UP
ULBP2	1.413868	1.178388	3.125511	0.004922	0.052602	-2.2094	UP
VCAM1	1.510465	5.48384	5.062355	4.53E-05	0.002945	1.902478	UP
VDR	1.305667	3.431796	5.012101	5.12E-05	0.003143	1.907215	UP
VIPR1	-1.49916	4.545112	-3.52249	0.001918	0.029031	-1.68689	DOWN
VIPR2	-1.637	0.739348	-3.56547	0.00173	0.027258	-1.19499	DOWN
VIT	-1.29236	2.688508	-2.93437	0.007675	0.069067	-2.79007	DOWN
VMO1	1.419531	2.752822	3.802143	0.000976	0.018915	-0.87338	UP
VNN2	1.283655	0.352877	3.130022	0.004871	0.052284	-2.11834	UP
VSNL1	2.146383	-0.36437	4.530417	0.000165	0.006379	0.946969	UP
VSTM2A	-1.24445	0.048794	-3.37223	0.002747	0.035996	-1.57147	DOWN
VTN	-1.42789	4.533344	-5.9774	5.14E-06	0.000923	4.101577	DOWN
VWA5B2	-2.52343	0.661592	-4.38371	0.000236	0.007986	0.634514	DOWN
WFDC2	1.205203	0.420367	2.767939	0.011222	0.086591	-2.87159	UP
WIF1	-3.44828	2.277821	-5.47428	1.68E-05	0.001762	3.078438	DOWN
WNK3	-1.39498	-0.11811	-2.81395	0.01011	0.081545	-2.71071	DOWN
WNT10B	1.699806	-1.01959	4.396554	0.000229	0.007844	0.629374	UP

WNT4	-1.76657	0.29808	-3.12636	0.004913	0.052556	-2.10446	DOWN
WNT5A	-1.26193	1.588545	-3.44878	0.002288	0.0324	-1.52995	DOWN
WSCD2	1.207914	1.623466	3.457062	0.002244	0.032051	-1.53201	UP
XCL2	1.725459	0.762726	3.812482	0.000952	0.018706	-0.65952	UP
XKR4	-1.79922	-0.90968	-3.46101	0.002222	0.031941	-1.35135	DOWN
ZAP70	1.202992	2.595625	4.361074	0.00025	0.008279	0.447159	UP
ZBTB16	-1.46205	7.137697	-2.70337	0.01298	0.094345	-3.59505	DOWN
ZDHHC11B	-1.25368	4.333381	-3.36499	0.002795	0.036534	-2.03103	DOWN
ZNF683	1.808279	-0.31866	3.521012	0.001925	0.029087	-1.24261	UP

LncRNA	logFC	AveExpr	t	P.Value	adj.P.Val	B	change
SFTA1P	3.062744	-0.28197	4.857078	7.45E-05	0.003975	1.656316	UP
LINC01614	2.851569	-0.14181	5.570768	1.34E-05	0.001558	3.191265	UP
APCDD1L-DT	2.362794	0.072065	4.973437	5.62E-05	0.003347	1.931414	UP
LINC02360	-2.35292	-1.16974	-4.35148	0.000256	0.008409	0.5142	DOWN
LINC02452	2.045881	0.426455	4.289175	0.000298	0.009189	0.422492	UP
LINC02814	-1.96621	-0.77501	-4.13269	0.000436	0.011462	0.069397	DOWN
CT62	-1.9244	-1.04997	-4.50167	0.000177	0.00672	0.83845	DOWN
LINC00702	1.847828	1.949535	4.2335	0.000341	0.009855	0.212364	UP
VIPR1-AS1	-1.82701	0.515653	-3.8126	0.000952	0.018706	-0.63472	DOWN
ACTA2-AS1	1.705017	2.777768	4.120386	0.00045	0.011666	-0.12841	UP
LINC00861	1.643225	-0.91448	4.000831	0.000602	0.014275	-0.21082	UP
LINC01013	1.603847	-1.37156	2.762292	0.011367	0.087104	-2.74695	UP
TRHDE-AS1	-1.59248	1.539463	-5.40448	1.99E-05	0.001895	2.922261	DOWN
LINC00092	-1.54364	2.778554	-5.07761	4.37E-05	0.002939	2.127116	DOWN
FAM225B	1.525263	-0.13001	5.21826	3.11E-05	0.002479	2.450762	UP
PICART1	1.461259	-0.97291	4.223435	0.00035	0.010042	0.262178	UP
LINC02584	1.418912	-1.36428	3.205236	0.004082	0.046569	-1.87184	UP
ALDH1L1-AS2	-1.35994	-0.69899	-2.76111	0.011397	0.087197	-2.77615	DOWN
CARMN	1.342945	4.905044	2.856564	0.009174	0.076689	-3.21673	UP
C10orf55	1.336816	-0.12297	3.884758	0.000799	0.01684	-0.46185	UP
LINC01252	1.334013	-1.03002	3.271021	0.003494	0.042059	-1.74489	UP
LINC01354	-1.33238	0.596166	-4.04836	0.000536	0.013121	-0.11667	DOWN
FAM242C	1.322773	-1.7283	3.295408	0.003298	0.040478	-1.68553	UP
LINC02585	-1.31866	-0.69453	-3.07922	0.005485	0.056047	-2.14433	DOWN
LINC00968	-1.31746	-1.10254	-2.8064	0.010285	0.08256	-2.66682	DOWN
CDKN2B-AS1	1.308002	-1.32378	3.180255	0.004329	0.048348	-1.92297	UP
LINC01605	1.306287	-0.67371	3.115009	0.005045	0.053317	-2.07823	UP
LINC01197	1.290004	0.836642	3.840678	0.000889	0.017912	-0.60279	UP
LINC01094	1.275185	1.700553	4.780116	8.99E-05	0.004431	1.490837	UP
RAMP2-AS1	-1.23553	1.934866	-3.98394	0.000627	0.014638	-0.35498	DOWN
LINC01220	-1.22799	-1.21394	-3.20774	0.004057	0.046434	-1.86699	DOWN
SOCS2-AS1	-1.21313	-1.14647	-2.74348	0.01186	0.089374	-2.78643	DOWN
ZFP28-DT	-1.20896	0.133803	-4.86071	7.39E-05	0.003951	1.683126	DOWN
LINC00484	-1.2018	-1.13442	-3.29207	0.003324	0.040707	-1.69718	DOWN

LINC01152	-1.19847	-0.40734	-3.76501	0.001068	0.020097	-0.71432	DOWN
STK32A-AS1	-1.19763	-1.08172	-3.64937	0.001413	0.023852	-0.95774	DOWN

Supplementary Table 4 The differentially expressed mRNAs and LncRNAs in GSE148219

mRNAs	Log FC	AveExpr	t	P.Value	adj.P.Val	B	change
A2M	1.464373	9.115592	6.276293	9.79E-06	0.00021	3.514204	UP
ABCA8	-1.57616	7.938232	-9.55399	4.09E-08	1.18E-05	9.003039	DOWN
ACADL	-1.64319	3.733926	-6.89252	3.14E-06	0.00011	4.783172	DOWN
ACP5	2.277369	4.853095	4.656583	0.000246	0.00189	0.414673	UP
ACTG2	1.408382	4.659446	2.261246	0.037639	0.081904	-4.46478	UP
ADAM12	1.898319	3.49077	4.53452	0.000318	0.002284	0.292126	UP
ADAM33	-1.68749	5.560385	-8.66881	1.57E-07	2.12E-05	7.67763	DOWN
ADAM8	1.620223	2.729043	6.331003	8.83E-06	0.000199	3.808735	UP
ADAMTS12	1.881569	3.270198	5.197767	8.10E-05	0.000858	1.64463	UP
ADAMTS19	-2.49813	3.297046	-7.27902	1.58E-06	7.30E-05	5.46154	DOWN
ADAMTS4	-1.94576	4.976044	-3.99454	0.000997	0.005238	-1.02892	DOWN
ADAP2	1.671073	4.521137	7.353668	1.39E-06	6.84E-05	5.564057	UP
ADCY1	1.568127	2.935628	5.067095	0.000106	0.001029	1.404964	UP
ADCYAP1R1	-1.81868	2.775762	-7.00349	2.57E-06	9.60E-05	4.990834	DOWN
ADGRD1	-1.46821	5.769362	-7.7896	6.58E-07	4.49E-05	6.240426	DOWN
ADGRE2	2.090577	2.864577	6.271166	9.89E-06	0.000212	3.699829	UP
ADGRG2	-1.85422	3.748986	-9.58001	3.94E-08	1.16E-05	9.015867	DOWN
ADGRL3	1.640301	2.011348	4.378248	0.000442	0.002908	0.085024	UP
ADGRL4	1.83008	3.454267	5.432417	5.05E-05	0.000617	2.093473	UP
ADM	-1.64854	3.298889	-5.59345	3.67E-05	0.000508	2.3971	DOWN
ADRA2A	2.217867	2.673663	9.63068	3.66E-08	1.10E-05	8.91974	UP
ADRB1	-1.43852	2.762528	-5.40801	5.30E-05	0.000637	2.070197	DOWN
AIF1	1.49294	5.8376	7.420297	1.24E-06	6.40E-05	5.613188	UP
AKR1C1	-1.83343	7.684068	-6.62878	5.08E-06	0.000148	4.163122	DOWN
ALDH1L1	-1.99404	3.553395	-5.38813	5.52E-05	0.000654	1.979826	DOWN
ALDH3A1	-1.86189	2.551671	-5.46618	4.72E-05	0.00059	2.192618	DOWN
ALOX5	1.563151	4.707283	8.068098	4.14E-07	3.48E-05	6.749321	UP
ALPK2	1.515204	1.942063	3.503351	0.002845	0.011344	-1.68249	UP
AMPD3	1.377051	3.40431	5.506404	4.36E-05	0.000565	2.236145	UP
ANGPT2	1.928128	3.402468	7.499321	1.08E-06	6.05E-05	5.828791	UP
ANGPTL4	-1.6978	4.537212	-4.51654	0.00033	0.002355	0.105484	DOWN
ANGPTL5	-1.9268	3.062854	-8.83462	1.21E-07	1.95E-05	7.903976	DOWN
ANGPTL7	-3.29783	4.72899	-8.25081	3.07E-07	2.89E-05	7.048721	DOWN
ANKFN1	-1.71812	2.134543	-6.734	4.19E-06	0.00013	4.515667	DOWN
ANKRD45	-1.44512	1.915421	-6.08879	1.40E-05	0.000267	3.371195	DOWN
ANLN	1.824374	2.595149	4.189239	0.000659	0.003895	-0.33768	UP
APCDD1	-1.55965	8.512391	-7.52772	1.03E-06	5.91E-05	5.780933	DOWN

APCDD1L	1.663048	3.155994	4.805911	0.000181	0.001509	0.867915	UP
APLN	1.623728	2.825077	4.393719	0.000428	0.002835	0.058389	UP
APOBR	1.524564	3.223405	4.60394	0.000275	0.002052	0.451696	UP
APOC1	1.529753	3.594591	3.186462	0.00559	0.019028	-2.50475	UP
AQP2	-1.88011	2.923164	-3.93989	0.00112	0.005681	-0.90772	DOWN
AQP7	-1.74683	2.647118	-3.66713	0.002005	0.008759	-1.43975	DOWN
ARHGAP18	1.455619	5.311416	8.261727	3.01E-07	2.85E-05	7.041736	UP
ARHGAP30	1.936177	5.139608	10.10094	1.86E-08	7.52E-06	9.785639	UP
ARID5A	-1.40107	6.377319	-5.92622	1.91E-05	0.000329	2.832448	DOWN
ARL11	1.968358	2.090115	7.649097	8.35E-07	5.20E-05	6.002359	UP
ATF3	-2.34087	5.114664	-6.48712	6.60E-06	0.00017	3.975297	DOWN
ATOH8	-1.38501	5.448864	-3.80913	0.00148	0.006978	-1.47693	DOWN
ATP1A2	-2.62413	5.219592	-6.24352	1.04E-05	0.000218	3.518838	DOWN
ATP1B2	-1.56152	3.954532	-7.19069	1.85E-06	7.94E-05	5.295559	DOWN
ATRNL1	-1.60005	3.779691	-6.40264	7.72E-06	0.000186	3.896863	DOWN
AVPI1	-1.39157	4.068742	-5.49369	4.47E-05	0.000573	2.13595	DOWN
B4GALNT4	-1.65842	2.256249	-5.61342	3.52E-05	0.000497	2.485731	DOWN
BAMBI	-1.9276	4.931474	-10.1507	1.73E-08	7.21E-06	9.85526	DOWN
BCAT1	1.910649	5.659862	10.19781	1.62E-08	7.21E-06	9.924396	UP
BCL11B	1.474961	1.648143	4.73104	0.000211	0.001682	0.802692	UP
BEX2	-1.41859	2.961834	-6.57637	5.59E-06	0.000153	4.24291	DOWN
BEX5	-1.54658	2.385456	-5.66394	3.19E-05	0.000469	2.577764	DOWN
BIN2	1.646262	3.807619	6.109043	1.35E-05	0.000263	3.365778	UP
BLNK	1.399084	2.862318	5.805422	2.42E-05	0.000383	2.836474	UP
BRSK2	-2.41767	3.431266	-6.75762	4.01E-06	0.000127	4.556086	DOWN
BTC	-1.44097	2.477749	-4.98054	0.000126	0.001164	1.247761	DOWN
BTG2	-2.19456	8.420339	-9.2478	6.45E-08	1.44E-05	8.55332	DOWN
BUB1	1.785831	1.902323	5.015907	0.000117	0.001105	1.354449	UP
C11orf87	-1.38918	1.536425	-5.36085	5.83E-05	0.000679	2.020456	DOWN
C11orf96	-2.1079	6.201055	-8.79645	1.29E-07	1.97E-05	7.86803	DOWN
C1orf162	1.426689	4.034655	6.550388	5.87E-06	0.000159	4.164185	UP
C1QB	1.782526	7.516347	5.549037	4.00E-05	0.000534	2.078428	UP
C1QC	1.591132	7.516313	5.25583	7.20E-05	0.000789	1.486117	UP
C2	1.38631	5.843403	7.212504	1.78E-06	7.86E-05	5.247114	UP
C3AR1	1.616661	4.934162	7.14893	1.99E-06	8.26E-05	5.185337	UP
C5orf46	1.9142	2.073391	6.978043	2.69E-06	9.90E-05	4.916305	UP
C6	-2.48684	4.559366	-8.80887	1.26E-07	1.97E-05	7.915068	DOWN
C8orf34	-1.53156	2.357078	-6.39131	7.89E-06	0.000188	3.917265	DOWN
CA12	1.997639	3.957043	4.756094	0.0002	0.00162	0.705335	UP
CA3	-2.03122	4.557852	-4.62697	0.000262	0.001976	0.342048	DOWN
CAB39L	-1.58818	5.756477	-8.74609	1.39E-07	2.06E-05	7.793338	DOWN
CACNA1H	1.707124	2.282122	4.991231	0.000123	0.001147	1.292021	UP
CACNA2D2	-1.62298	3.924583	-6.705	4.42E-06	0.000135	4.439397	DOWN
CADM2	-2.31529	3.183961	-8.26611	2.99E-07	2.85E-05	7.056097	DOWN

CADM3	-1.6812	7.177607	-6.44517	7.13E-06	0.000178	3.818288	DOWN
CADM4	-1.44571	3.149703	-5.20465	7.98E-05	0.000852	1.645446	DOWN
CBLN1	-2.38695	2.122257	-6.7436	4.11E-06	0.000129	4.532832	DOWN
CBS	-1.84749	4.000473	-3.00723	0.008166	0.025527	-2.95338	DOWN
CBX2	1.503759	1.620798	6.524676	6.15E-06	0.000165	4.129625	UP
CCDC141	-1.52809	3.514115	-7.12818	2.06E-06	8.33E-05	5.200939	DOWN
CCL13	1.496516	3.23306	2.861637	0.011084	0.03225	-3.10868	UP
CCL14	1.49544	3.209419	3.682101	0.001942	0.008586	-1.44387	UP
CCL18	2.510994	5.276783	3.405495	0.003507	0.013316	-2.23605	UP
CCL19	2.449703	4.143602	8.303058	2.82E-07	2.84E-05	7.128695	UP
CCL8	1.595589	3.04934	3.846364	0.001367	0.006569	-1.08664	UP
CCN1	-1.51367	8.84725	-7.38073	1.32E-06	6.67E-05	5.5294	DOWN
CCN5	-1.5476	6.702244	-3.17151	0.00577	0.019512	-2.86242	DOWN
CCNB2	1.519912	2.159017	5.018889	0.000117	0.001101	1.350704	UP
CCR1	2.37511	4.450369	8.230062	3.17E-07	2.93E-05	7.017571	UP
CCR2	2.310459	2.077405	10.0206	2.08E-08	7.99E-06	9.297178	UP
CCR5	2.430624	2.584065	8.675037	1.56E-07	2.12E-05	7.587885	UP
CCRL2	1.409396	1.858442	4.367912	0.000451	0.002956	0.070707	UP
CD14	1.481141	7.160493	7.662704	8.15E-07	5.15E-05	6.005061	UP
CD163	2.099041	7.491726	5.134273	9.21E-05	0.00094	1.241822	UP
CD180	1.606324	2.489755	4.974696	0.000128	0.001173	1.248844	UP
CD2	1.880236	2.045002	6.309651	9.20E-06	0.000204	3.7652	UP
CD209	1.986304	5.097357	5.602453	3.60E-05	0.000504	2.29291	UP
CD28	1.563137	4.213278	6.411241	7.60E-06	0.000186	3.900099	UP
CD300A	1.56085	3.178803	5.830015	2.30E-05	0.000372	2.870865	UP
CD300C	1.442301	1.856374	4.542444	0.000313	0.002256	0.420426	UP
CD300E	1.764115	2.052323	4.199656	0.000644	0.003833	-0.27688	UP
CD3D	1.483285	1.655427	5.22182	7.71E-05	0.00083	1.756669	UP
CD3E	1.433842	2.470534	4.256659	0.000571	0.003512	-0.19319	UP
CD48	1.511443	2.567217	4.871537	0.000158	0.001374	1.039437	UP
CD52	2.137413	3.621	5.349824	5.96E-05	0.00069	1.925154	UP
CD53	1.839284	5.312757	7.224519	1.74E-06	7.74E-05	5.302024	UP
CD68	2.18998	6.96459	6.389633	7.91E-06	0.000188	3.723233	UP
CD84	2.016864	5.085667	5.470305	4.68E-05	0.00059	2.033115	UP
CD86	1.410283	4.049816	5.021135	0.000116	0.001098	1.216123	UP
CD93	2.089358	6.956223	9.636078	3.63E-08	1.10E-05	9.124547	UP
CDC20	1.469953	1.873869	3.409449	0.003477	0.013221	-1.86686	UP
CDCP1	1.44044	2.947026	4.43125	0.000395	0.00267	0.122903	UP
CDH19	-1.77307	6.900941	-6.00732	1.63E-05	0.000296	2.984563	DOWN
CDH6	1.868786	2.604216	6.985955	2.66E-06	9.83E-05	4.951981	UP
CDK1	1.849681	2.674718	4.876649	0.000156	0.001364	1.045121	UP
CDKN2B	1.623484	2.911405	7.718834	7.41E-07	4.86E-05	6.172058	UP
CEBPD	-2.00663	6.888448	-11.278	3.78E-09	4.35E-06	11.35739	DOWN
CECR2	-1.37384	3.292415	-6.01898	1.60E-05	0.000294	3.209699	DOWN

CEMIP	1.965745	5.808845	10.78566	7.24E-09	5.41E-06	10.7144	UP
CENPE	1.611647	2.084977	5.125768	9.37E-05	0.000948	1.562091	UP
CENPF	2.082067	3.101432	4.445557	0.000383	0.002616	0.146363	UP
CERKL	1.826455	1.687554	9.231506	6.61E-08	1.46E-05	8.239064	UP
CERS1	-1.81779	3.186999	-6.0008	1.66E-05	0.000298	3.180773	DOWN
CFAP91	-1.43948	3.240993	-8.71311	1.47E-07	2.07E-05	7.737301	DOWN
CFD	-1.46752	5.079918	-4.17039	0.000686	0.004013	-0.68212	DOWN
CHAD	-2.60845	4.883863	-5.86379	2.16E-05	0.000358	2.815595	DOWN
CHADL	-1.80193	2.417326	-6.41005	7.62E-06	0.000186	3.950789	DOWN
CHI3L1	2.744754	4.506943	4.336023	0.000483	0.0031	-0.18823	UP
CHIT1	1.998276	2.379059	2.995896	0.008363	0.025986	-2.73436	UP
CHRDL1	-1.73367	4.251828	-4.83125	0.000171	0.001453	0.788589	DOWN
CHRDL2	2.086267	2.610853	5.588163	3.70E-05	0.000511	2.435984	UP
CHRM2	-1.55254	1.639491	-4.60063	0.000277	0.002059	0.535926	DOWN
CHST9	-2.99747	2.611154	-9.52439	4.27E-08	1.18E-05	8.818678	DOWN
CILP	-2.50132	5.437929	-7.56128	9.70E-07	5.71E-05	5.878553	DOWN
CKMT2	-1.4668	3.076916	-3.24213	0.004966	0.017352	-2.35848	DOWN
CLDN11	-1.49372	6.248392	-5.53225	4.14E-05	0.000546	2.058548	DOWN
CLEC3B	-1.37977	7.144717	-4.20598	0.000636	0.003797	-0.6972	DOWN
CLEC4G	2.039231	2.074621	5.160702	8.73E-05	0.000904	1.631438	UP
CLEC5A	2.15557	3.297397	7.694974	7.72E-07	4.96E-05	6.145361	UP
CLEC7A	1.403018	3.5537	6.382303	8.02E-06	0.000189	3.88183	UP
CLP1	1.380299	2.97362	7.704869	7.59E-07	4.89E-05	6.153727	UP
CLU	-2.11079	12.64712	-9.29879	5.98E-08	1.38E-05	8.64644	DOWN
CMYA5	-2.00855	3.982993	-6.65942	4.80E-06	0.000142	4.357577	DOWN
CNTFR	-2.78588	3.336041	-5.78643	2.51E-05	0.000393	2.771539	DOWN
CNTN1	-2.68964	4.212504	-8.7183	1.46E-07	2.07E-05	7.774076	DOWN
COBL	-1.4307	3.260642	-5.67468	3.12E-05	0.000461	2.556387	DOWN
COCH	-2.77118	3.596592	-5.60268	3.60E-05	0.000504	2.40353	DOWN
COL10A1	3.592226	2.696412	8.674766	1.56E-07	2.12E-05	7.579528	UP
COL11A1	3.460269	4.621108	7.644373	8.41E-07	5.22E-05	6.070833	UP
COL11A2	-1.62336	3.310341	-5.8189	2.35E-05	0.000376	2.829882	DOWN
COL28A1	-1.60736	1.763415	-6.2323	1.06E-05	0.000222	3.627601	DOWN
COL4A1	1.499392	9.405847	7.04653	2.38E-06	9.19E-05	4.942353	UP
COL4A3	-2.47469	4.345148	-6.7466	4.09E-06	0.000129	4.501916	DOWN
COL4A4	-1.84189	4.660662	-5.8493	2.22E-05	0.000364	2.785719	DOWN
COL4A5	-1.6063	4.524838	-6.04949	1.51E-05	0.000283	3.178002	DOWN
COL4A6	-1.6931	2.3623	-5.13287	9.24E-05	0.000943	1.553974	DOWN
COL6A6	-2.65557	6.170019	-9.26523	6.29E-08	1.42E-05	8.585967	DOWN
COL9A1	-2.82705	2.560793	-6.50217	6.42E-06	0.000168	4.114751	DOWN
COL9A2	-1.8481	5.242983	-7.30125	1.52E-06	7.07E-05	5.426679	DOWN
COL9A3	-2.58877	4.02333	-6.41871	7.49E-06	0.000184	3.923397	DOWN
COLGALT2	-1.68681	6.200787	-8.8411	1.20E-07	1.95E-05	7.934506	DOWN
CORO1A	1.541893	4.951657	7.30441	1.51E-06	7.07E-05	5.45482	UP

CPAMD8	-2.40144	6.428232	-5.14093	9.09E-05	0.000931	1.275699	DOWN
CPVL	1.785546	5.853324	10.1686	1.69E-08	7.21E-06	9.88442	UP
CPZ	1.874509	2.156882	5.339986	6.08E-05	0.000698	1.974211	UP
CR1	2.821929	3.490448	11.10115	4.76E-09	4.45E-06	10.86865	UP
CRISPLD2	-1.50691	6.129113	-6.30119	9.34E-06	0.000206	3.561461	DOWN
CRYBG1	1.392918	3.819853	7.18708	1.86E-06	7.94E-05	5.300234	UP
CSDC2	-1.4073	3.486455	-3.36502	0.003823	0.01423	-2.15989	DOWN
CSF1R	1.520311	7.216801	7.93215	5.18E-07	3.92E-05	6.460824	UP
CSMD2	-1.49453	3.818751	-9.64735	3.57E-08	1.10E-05	9.117939	DOWN
CSRNP1	-1.90802	5.614556	-9.99986	2.15E-08	8.03E-06	9.649749	DOWN
CTH	-1.55006	2.446673	-4.41128	0.000412	0.002753	0.105329	DOWN
CTHRC1	2.592884	4.97035	14.04777	1.41E-10	1.01E-06	14.32747	UP
CTSB	1.493657	10.37725	6.017287	1.60E-05	0.000294	3.044509	UP
CTSC	1.529761	7.322089	7.405635	1.27E-06	6.45E-05	5.560032	UP
CTSS	1.725501	6.235041	5.565088	3.88E-05	0.000525	2.135799	UP
CX3CR1	1.716697	3.597822	6.161922	1.22E-05	0.000245	3.476183	UP
CXCL12	1.649394	8.437258	8.189425	3.39E-07	3.02E-05	6.891381	UP
CYB561D1	1.410795	3.805583	9.944072	2.32E-08	8.32E-06	9.503641	UP
CYBB	2.021764	6.576389	7.271019	1.60E-06	7.30E-05	5.338412	UP
CYTH4	1.583981	5.244479	8.097568	3.94E-07	3.37E-05	6.779251	UP
CYTL1	-1.96479	5.192026	-6.67931	4.63E-06	0.000138	4.317411	DOWN
DAPP1	1.470698	2.038416	5.87117	2.13E-05	0.000357	2.973526	UP
DDIT4	-2.11508	6.801391	-10.3015	1.40E-08	7.01E-06	10.06508	DOWN
DDIT4L	1.738558	2.279559	7.178261	1.89E-06	8.00E-05	5.260345	UP
DENND1C	1.375152	2.395033	6.496959	6.48E-06	0.000169	4.103261	UP
DEPP1	-2.12692	8.005132	-6.56395	5.72E-06	0.000156	4.047612	DOWN
DERL3	1.762162	2.280242	5.306266	6.50E-05	0.00073	1.905567	UP
DIPK2B	2.046552	2.204635	5.340152	6.07E-05	0.000698	1.97372	UP
DLL4	1.616033	2.442165	5.152705	8.87E-05	0.000913	1.600903	UP
DOCK10	1.429839	4.259483	6.263154	1.00E-05	0.000215	3.618738	UP
DOCK2	1.529751	5.081513	6.833933	3.49E-06	0.000118	4.612423	UP
DOCK3	-1.45472	2.451368	-6.36193	8.34E-06	0.000193	3.864576	DOWN
DOCK8	1.411558	4.851773	5.674662	3.12E-05	0.000461	2.437834	UP
DOK3	1.73823	2.990288	6.625792	5.11E-06	0.000148	4.333365	UP
DPT	-1.4823	8.786185	-5.15297	8.87E-05	0.000913	1.294154	DOWN
DUSP1	-2.1913	8.009631	-8.29808	2.84E-07	2.84E-05	7.069021	DOWN
DUSP2	-1.7438	1.770862	-6.75583	4.02E-06	0.000128	4.540736	DOWN
DUSP26	-1.61586	2.37463	-4.06971	0.000849	0.004679	-0.58575	DOWN
DUSP5	-1.50346	4.114173	-6.61425	5.22E-06	0.000149	4.262351	DOWN
DUSP6	1.626142	5.546205	11.60175	2.49E-09	3.93E-06	11.74666	UP
EDNRA	1.795122	3.417047	5.312525	6.42E-05	0.000723	1.860328	UP
EMILIN3	-2.6037	4.388615	-8.58701	1.79E-07	2.20E-05	7.575317	DOWN
ENPEP	1.993252	1.900265	4.687153	0.000231	0.001811	0.709213	UP
ENPP2	1.433329	5.40452	6.349631	8.53E-06	0.000195	3.695167	UP

EPB41L4B	-2.3262	2.850844	-8.2924	2.87E-07	2.84E-05	7.079305	DOWN
EPHA7	-1.56272	3.306924	-4.69086	0.000229	0.0018	0.598871	DOWN
ERBB3	-2.76578	4.420714	-8.64457	1.63E-07	2.12E-05	7.663687	DOWN
EVI2B	1.838403	3.510582	7.010005	2.54E-06	9.54E-05	5.001586	UP
EYA1	-1.59698	2.500929	-5.46525	4.73E-05	0.000591	2.193712	DOWN
F10	-1.73033	5.16874	-7.46996	1.13E-06	6.13E-05	5.72133	DOWN
F13A1	1.975311	8.680228	5.602835	3.60E-05	0.000504	2.198472	UP
FAIM2	-1.38757	3.995704	-4.31502	0.000505	0.003206	-0.25457	DOWN
FAM107A	-1.45201	4.930214	-4.18569	0.000664	0.003916	-0.63545	DOWN
FAM135B	-1.52214	2.005826	-6.29145	9.52E-06	0.000207	3.736476	DOWN
FAM43B	-1.49965	2.886358	-7.71621	7.45E-07	4.86E-05	6.177468	DOWN
FAM78A	1.53328	3.761186	7.485494	1.10E-06	6.07E-05	5.807688	UP
FAP	1.565993	5.376569	7.323627	1.46E-06	6.98E-05	5.466472	UP
FBP1	1.748573	2.474655	5.732569	2.79E-05	0.000426	2.711493	UP
FBXO2	-2.89842	3.633625	-8.53273	1.95E-07	2.27E-05	7.47788	DOWN
FCER1G	1.50259	5.768889	5.438912	4.98E-05	0.000614	1.901166	UP
FCGBP	2.192251	5.96005	8.175483	3.47E-07	3.03E-05	6.892018	UP
FCGR1A	2.777846	4.140019	11.85785	1.81E-09	3.38E-06	11.85098	UP
FCGR1B	2.646547	3.056492	7.924278	5.25E-07	3.95E-05	6.496263	UP
FCGR2A	1.62309	6.788481	5.14238	9.06E-05	0.00093	1.263559	UP
FCGR3A	2.896053	6.498274	12.41438	9.13E-10	2.28E-06	12.72225	UP
FCGR3B	2.678818	2.263573	6.117078	1.32E-05	0.00026	3.422397	UP
FCN1	2.413027	2.377062	6.729814	4.22E-06	0.00013	4.505964	UP
FGFBP2	-2.42132	4.895916	-6.65946	4.80E-06	0.000142	4.309071	DOWN
FLT4	2.07831	2.279199	7.121145	2.09E-06	8.35E-05	5.164197	UP
FMO3	1.782017	3.128442	6.862383	3.32E-06	0.000114	4.747653	UP
FMOD	-1.44054	10.91602	-6.82447	3.55E-06	0.000119	4.574032	DOWN
FN3K	-1.63546	3.621539	-7.63856	8.50E-07	5.23E-05	6.06158	DOWN
FOS	-3.24543	8.33935	-9.92383	2.39E-08	8.32E-06	9.539909	DOWN
FOSB	-3.25908	4.799345	-9.43771	4.86E-08	1.20E-05	8.843039	DOWN
FPR1	1.864618	4.018075	6.065813	1.46E-05	0.000277	3.275037	UP
FPR3	1.904238	4.244379	5.627216	3.43E-05	0.000491	2.416576	UP
FREM1	-1.97621	5.14833	-8.59781	1.76E-07	2.19E-05	7.578702	DOWN
FRRS1L	-2.59948	2.490493	-7.18687	1.86E-06	7.94E-05	5.295847	DOWN
FRZB	-1.45549	7.311329	-5.01488	0.000118	0.001105	0.992699	DOWN
FXYD1	-1.51277	6.502403	-5.55516	3.95E-05	0.00053	2.098508	DOWN
FYB1	1.878927	5.083464	8.340381	2.65E-07	2.75E-05	7.179984	UP
GABARAPL1	-1.47626	7.336527	-10.0543	1.99E-08	7.82E-06	9.71971	DOWN
GADD45B	-2.33036	6.927954	-10.3944	1.23E-08	7.01E-06	10.19248	DOWN
GADD45G	-2.20943	3.107565	-6.08951	1.40E-05	0.000267	3.350716	DOWN
GALNT16	-1.69292	5.736639	-8.29281	2.87E-07	2.84E-05	7.075021	DOWN
GALNT6	1.398303	3.016336	5.099133	9.89E-05	0.000986	1.461948	UP
GBP5	1.775541	2.82808	5.546251	4.03E-05	0.000535	2.346627	UP
GCNT2	1.371317	1.637858	4.280049	0.000544	0.003401	-0.09504	UP

GDF7	1.498775	2.22692	4.364544	0.000455	0.002966	0.043556	UP
GDNF	1.388555	1.810193	6.599135	5.36E-06	0.00015	4.265503	UP
GFAP	-2.90015	6.135022	-5.4901	4.50E-05	0.000575	2.004984	DOWN
GIMAP1	1.837861	3.366401	7.185417	1.86E-06	7.94E-05	5.302805	UP
GIMAP2	1.447065	3.80601	8.001825	4.62E-07	3.67E-05	6.652351	UP
GIMAP4	1.844686	5.079872	7.414048	1.25E-06	6.40E-05	5.643877	UP
GIMAP6	1.991809	4.623684	9.440584	4.84E-08	1.20E-05	8.845942	UP
GIMAP7	1.970757	3.735048	7.59269	9.19E-07	5.48E-05	5.985899	UP
GIMAP8	1.53866	4.589995	7.00545	2.56E-06	9.59E-05	4.950435	UP
GJA5	1.522486	5.327999	5.50675	4.35E-05	0.000565	2.068138	UP
GLIS1	-1.70999	3.311204	-6.66041	4.79E-06	0.000142	4.384821	DOWN
GMFG	1.474309	4.32472	7.273611	1.60E-06	7.30E-05	5.432211	UP
GPM6A	-2.01437	2.096607	-5.43753	5.00E-05	0.000614	2.154751	DOWN
GPR34	1.83037	4.601378	7.061778	2.32E-06	9.01E-05	5.055133	UP
GPR65	1.846844	1.940822	8.011201	4.55E-07	3.64E-05	6.544703	UP
GPR68	1.816028	2.918868	6.47239	6.78E-06	0.000172	4.061205	UP
GPR83	-1.46965	3.295093	-5.2139	7.84E-05	0.00084	1.652721	DOWN
GPRC5A	-2.239	4.313146	-8.45983	2.19E-07	2.46E-05	7.378166	DOWN
GPRIN3	1.492124	4.318426	6.280676	9.71E-06	0.00021	3.648369	UP
GPSM1	-1.43443	5.879526	-5.72128	2.85E-05	0.000433	2.445647	DOWN
GRAP	1.435936	2.478653	5.858879	2.18E-05	0.00036	2.946961	UP
GREM1	2.117926	2.294236	4.793323	0.000185	0.001534	0.902369	UP
GRIA3	-1.61747	4.34352	-10.695	8.18E-09	5.56E-06	10.55759	DOWN
GSN	-1.67273	11.20271	-5.83517	2.28E-05	0.000369	2.715881	DOWN
GSTM5	-1.94352	4.94661	-4.73631	0.000209	0.00167	0.52516	DOWN
GUCY1A2	1.431743	3.602043	5.318344	6.35E-05	0.000717	1.85396	UP
H2AC19	-3.18429	3.034039	-5.20559	7.97E-05	0.000851	1.661966	DOWN
H2BC21	-1.74025	3.772039	-6.64558	4.92E-06	0.000145	4.341056	DOWN
H2BU1	-2.257	2.030546	-5.40427	5.34E-05	0.000639	2.092627	DOWN
HAND2	-1.58582	6.751369	-6.88152	3.20E-06	0.000112	4.629256	DOWN
HAVCR2	1.561939	4.210573	5.510423	4.32E-05	0.000564	2.18078	UP
HBA2	2.295424	6.591392	3.339371	0.004038	0.014817	-2.49067	UP
HBB	2.322435	6.720577	3.216227	0.005247	0.018102	-2.75072	UP
HBEGF	-1.76804	4.782233	-5.96435	1.78E-05	0.000315	2.996538	DOWN
HCK	1.659882	4.518523	7.828239	6.17E-07	4.35E-05	6.363252	UP
HCLS1	1.487677	5.887533	8.226817	3.19E-07	2.93E-05	6.967739	UP
HES4	-1.47103	3.708033	-5.66916	3.16E-05	0.000465	2.513493	DOWN
HIF3A	-1.79533	3.877495	-4.20312	0.00064	0.003811	-0.46512	DOWN
HILPDA	-1.91023	4.14715	-6.21796	1.09E-05	0.000226	3.532394	DOWN
HK3	1.974412	3.009917	5.349479	5.96E-05	0.00069	1.958754	UP
HLA-DPA1	1.39439	7.900148	5.227773	7.62E-05	0.000822	1.430134	UP
HLA-DRA	1.727103	8.889351	6.37385	8.15E-06	0.00019	3.696544	UP
HLA-DRB1	1.463314	7.527891	5.805309	2.42E-05	0.000383	2.585161	UP
HMGCLL1	-1.5765	2.419894	-5.86021	2.17E-05	0.00036	2.945926	DOWN

HMOX1	1.717807	5.928519	3.261128	0.00477	0.016846	-2.63335	UP
HOPX	1.690958	3.527266	7.072695	2.27E-06	8.86E-05	5.109359	UP
HPD	-1.38984	3.383897	-6.00707	1.64E-05	0.000296	3.182351	DOWN
HPR	-1.44523	1.782909	-3.48449	0.002962	0.011682	-1.72644	DOWN
HR	-1.49772	3.088606	-3.86867	0.001303	0.006336	-1.07215	DOWN
HRH2	1.407635	2.123133	3.674597	0.001973	0.008671	-1.35091	UP
HS3ST2	1.993968	2.242435	4.681342	0.000234	0.001823	0.681579	UP
HSF4	-1.63202	4.286586	-6.61608	5.20E-06	0.000149	4.255673	DOWN
HSPA1A	-1.63201	7.975856	-4.82675	0.000173	0.001463	0.609906	DOWN
HSPA1B	-2.43484	7.936957	-5.65588	3.24E-05	0.000472	2.300791	DOWN
HTR4	-1.49953	4.175696	-7.04729	2.38E-06	9.19E-05	5.034253	DOWN
HTRA3	-1.89496	6.794571	-8.02474	4.44E-07	3.63E-05	6.619037	DOWN
IER3	-1.45546	6.689762	-5.83475	2.28E-05	0.000369	2.64837	DOWN
IFI30	2.008244	5.917953	5.554782	3.96E-05	0.00053	2.138274	UP
IGFBP2	1.576574	6.051189	3.875869	0.001284	0.006268	-1.35743	UP
IGLL5	2.683442	2.181228	3.34524	0.003987	0.014694	-2.01501	UP
IGSF10	-2.79019	5.262471	-9.48896	4.50E-08	1.20E-05	8.92368	DOWN
IGSF11	-1.59849	1.855995	-6.49059	6.56E-06	0.00017	4.085632	DOWN
IGSF3	-1.48022	6.335477	-7.47595	1.12E-06	6.13E-05	5.691016	DOWN
IGSF6	1.90872	4.064036	7.432789	1.21E-06	6.39E-05	5.716588	UP
IKZF1	1.53481	3.704656	6.170187	1.20E-05	0.000243	3.484423	UP
IKZF3	1.681682	1.925785	4.554436	0.000305	0.002218	0.442625	UP
IL12RB2	1.513047	1.858202	5.178914	8.41E-05	0.000877	1.670575	UP
IL18	1.459424	2.774903	6.129831	1.29E-05	0.000256	3.442267	UP
IL2RA	1.658618	2.494011	4.031531	0.000921	0.004959	-0.65264	UP
IL2RB	1.460519	2.245429	6.103349	1.36E-05	0.000265	3.398042	UP
IL7R	1.891722	3.0298	4.6263	0.000263	0.001977	0.517347	UP
INHBA	1.731101	6.558973	5.958848	1.79E-05	0.000316	2.901088	UP
IPCEF1	1.474322	1.491995	5.86291	2.16E-05	0.000358	2.955236	UP
IRF6	-1.39093	4.843349	-7.03275	2.44E-06	9.34E-05	4.968184	DOWN
IRF8	1.47006	4.487085	4.888783	0.000152	0.001338	0.899281	UP
ITGA4	1.874406	3.631028	8.407579	2.38E-07	2.58E-05	7.280035	UP
ITGAL	1.683104	3.248244	6.282923	9.67E-06	0.000209	3.71201	UP
ITGAX	1.418533	4.280942	3.793516	0.00153	0.007162	-1.35209	UP
ITGB2	2.020124	6.276025	9.063248	8.54E-08	1.66E-05	8.28005	UP
ITGB7	1.407186	1.649837	5.691365	3.02E-05	0.000449	2.641049	UP
ITIH5	-1.58858	5.086938	-6.51811	6.23E-06	0.000166	4.019596	DOWN
ITLN1	-2.52838	3.123563	-4.05874	0.000869	0.004756	-0.6792	DOWN
ITM2A	-1.51854	6.344588	-6.25281	1.02E-05	0.000217	3.464228	DOWN
JAG2	1.418851	2.161103	4.882252	0.000154	0.001352	1.081563	UP
JAML	1.58373	3.626441	5.973677	1.74E-05	0.00031	3.120771	UP
JCHAIN	3.960134	3.318721	5.508991	4.33E-05	0.000564	2.275705	UP
JUN	-1.51678	8.031695	-7.30294	1.52E-06	7.07E-05	5.383596	DOWN
JUNB	-2.06979	8.035918	-9.01581	9.18E-08	1.70E-05	8.199463	DOWN

KCNJ15	1.378111	2.24501	3.954528	0.001085	0.005558	-0.79112	UP
KCNJ5	1.715448	4.107753	6.623076	5.13E-06	0.000148	4.296696	UP
KCNK17	-1.6238	4.156245	-4.8229	0.000174	0.001472	0.77992	DOWN
KCNQ1	1.461903	3.408449	6.292659	9.49E-06	0.000207	3.723552	UP
KCNQ3	1.483493	2.017481	6.100851	1.37E-05	0.000265	3.392013	UP
KIAA0040	-1.53518	6.410635	-8.5642	1.86E-07	2.22E-05	7.49688	DOWN
KIF11	1.884301	2.137226	5.850767	2.21E-05	0.000364	2.935839	UP
KIF1A	-2.3233	3.150224	-8.37331	2.52E-07	2.65E-05	7.219206	DOWN
KIF20A	1.393995	2.256992	2.894874	0.01034	0.030632	-2.92525	UP
KIF20B	1.371251	2.892646	5.695433	3.00E-05	0.000448	2.626981	UP
KIF23	1.49469	2.617989	6.473855	6.76E-06	0.000172	4.064365	UP
KIF4A	1.501411	1.997508	5.27694	6.90E-05	0.000764	1.856107	UP
KIF5A	-1.57591	2.815939	-8.11079	3.86E-07	3.32E-05	6.797721	DOWN
KIF5C	-1.79415	3.406397	-7.80298	6.43E-07	4.44E-05	6.329998	DOWN
KIRREL3	-1.89501	2.773057	-6.09833	1.37E-05	0.000265	3.378412	DOWN
KLF15	-2.03804	4.268876	-4.45192	0.000378	0.002596	0.011661	DOWN
KLF4	-1.67061	6.242977	-10.1764	1.67E-08	7.21E-06	9.893077	DOWN
KRT17	1.881515	2.368064	7.758628	6.93E-07	4.65E-05	6.197799	UP
KRT18	2.555607	3.798316	6.622595	5.14E-06	0.000148	4.317396	UP
KYNU	1.462759	2.965067	4.455808	0.000375	0.002578	0.171617	UP
LAIR1	1.745052	5.307018	7.140784	2.02E-06	8.29E-05	5.152728	UP
LAMC3	-1.77975	6.266896	-6.14338	1.26E-05	0.000252	3.259935	DOWN
LAPTM5	1.753039	7.603916	6.101347	1.37E-05	0.000265	3.163249	UP
LCNL1	-1.83685	2.462019	-4.37401	0.000446	0.002928	0.026976	DOWN
LCP1	1.873849	6.492482	7.07441	2.27E-06	8.86E-05	4.989219	UP
LCP2	1.744495	4.636222	9.080913	8.31E-08	1.66E-05	8.323433	UP
LGALS9	1.534175	4.8931	7.690947	7.77E-07	4.97E-05	6.118617	UP
LGI2	1.408286	4.101693	6.339478	8.69E-06	0.000197	3.771976	UP
LGI4	-1.63478	4.64693	-5.50907	4.33E-05	0.000564	2.11455	DOWN
LILRB1	2.020239	3.023145	6.179514	1.18E-05	0.00024	3.529699	UP
LILRB2	1.454319	3.623648	5.693758	3.01E-05	0.000448	2.58524	UP
LILRB4	1.881196	4.308568	5.524597	4.20E-05	0.000553	2.208978	UP
LIMS4	-1.82921	2.576922	-4.00118	0.000983	0.005193	-0.74635	DOWN
LMNB1	1.546899	3.433625	3.867037	0.001308	0.00635	-1.08543	UP
LONRF2	-1.58624	3.436994	-7.49108	1.09E-06	6.05E-05	5.817357	DOWN
LRFN5	-1.56906	2.313849	-7.86722	5.78E-07	4.21E-05	6.383978	DOWN
LRRC15	2.060807	3.875554	5.86418	2.16E-05	0.000358	2.905304	UP
LRRC25	1.763604	3.781959	6.325419	8.93E-06	0.000199	3.770754	UP
LRRC75B	-1.3969	3.619887	-5.90017	2.01E-05	0.000342	2.9644	DOWN
LST1	1.601157	3.25374	7.30263	1.52E-06	7.07E-05	5.500143	UP
LTB	1.562572	2.279089	5.643037	3.32E-05	0.000481	2.546763	UP
LTBP4	-1.62546	9.77651	-6.39255	7.87E-06	0.000188	3.752304	DOWN
LUM	1.385188	9.331643	5.617534	3.50E-05	0.000495	2.236443	UP
LY86	1.711512	2.86901	6.487408	6.59E-06	0.00017	4.088271	UP

LYZ	2.433917	6.332141	5.997612	1.67E-05	0.000299	2.998631	UP
MAFF	-1.87077	5.226166	-11.3228	3.56E-09	4.35E-06	11.39634	DOWN
MAL	-1.68935	1.905925	-5.79462	2.47E-05	0.000388	2.831732	DOWN
MAPK4	-1.69707	3.113751	-5.82633	2.32E-05	0.000373	2.854693	DOWN
MAPT	-2.58996	4.505989	-7.56723	9.60E-07	5.67E-05	5.92814	DOWN
MARCO	2.230889	4.049322	3.570185	0.002467	0.010221	-1.75137	UP
MASP1	-1.61698	6.143339	-6.21597	1.10E-05	0.000227	3.400165	DOWN
MATK	1.42404	1.666551	4.464951	0.000368	0.002538	0.273971	UP
MATN2	-1.64108	8.796729	-8.61605	1.71E-07	2.15E-05	7.582296	DOWN
MCTP1	1.442406	3.264115	5.035237	0.000113	0.001075	1.317593	UP
MFAP5	1.737159	4.01255	5.544141	4.04E-05	0.000537	2.269816	UP
MIA	-1.51241	5.625162	-5.08997	0.000101	0.000997	1.189926	DOWN
MKI67	2.143424	3.591371	4.358077	0.000461	0.002991	-0.07431	UP
MMP11	2.049695	3.201832	6.375901	8.12E-06	0.00019	3.883936	UP
MMP9	3.28296	3.69338	4.260267	0.000567	0.003495	-0.25626	UP
MMRN1	1.417381	3.341045	2.710525	0.015175	0.04112	-3.41866	UP
MNDA	2.030553	3.630491	7.841584	6.03E-07	4.35E-05	6.390384	UP
MPEG1	1.76078	6.348984	6.84647	3.41E-06	0.000116	4.579635	UP
MPPED2	-1.43149	4.167772	-8.61606	1.71E-07	2.15E-05	7.620169	DOWN
MRC1	1.622432	6.669138	4.904479	0.000147	0.00131	0.777388	UP
MS4A4A	1.894732	5.273704	6.238651	1.05E-05	0.000219	3.506818	UP
MS4A6A	1.51026	6.7348	6.394579	7.84E-06	0.000188	3.72919	UP
MS4A7	1.717616	6.254513	6.401701	7.74E-06	0.000186	3.757822	UP
MSR1	2.093482	5.951089	6.245002	1.04E-05	0.000218	3.482845	UP
MT1M	-2.82627	3.961983	-4.34496	0.000474	0.003057	-0.15716	DOWN
MT1X	-3.37319	6.352954	-6.32045	9.01E-06	0.000201	3.622445	DOWN
MT2A	-2.06336	6.916399	-5.58263	3.75E-05	0.000516	2.151174	DOWN
MT-ND5	1.769425	11.08062	9.302185	5.95E-08	1.38E-05	8.647206	UP
MT-ND6	2.338396	9.63774	11.19856	4.19E-09	4.45E-06	11.25273	UP
MYEF2	-1.44869	4.555912	-7.96059	4.94E-07	3.79E-05	6.570323	DOWN
MYH14	-1.93036	4.283072	-7.80258	6.44E-07	4.44E-05	6.322146	DOWN
MYH7B	-1.38361	2.78188	-4.14193	0.000728	0.004195	-0.47515	DOWN
MYO10	1.966804	4.339862	12.63995	6.98E-10	2.09E-06	12.80571	UP
MYO1F	1.58685	4.980288	9.17432	7.21E-08	1.49E-05	8.462706	UP
MYO1G	1.639297	3.17072	5.621116	3.47E-05	0.000493	2.473425	UP
MYO5C	1.674726	1.971819	5.575669	3.80E-05	0.00052	2.424463	UP
MYOC	-2.94631	4.019185	-6.69817	4.47E-06	0.000135	4.435637	DOWN
NAALAD2	-1.46893	2.944598	-7.80161	6.45E-07	4.44E-05	6.31673	DOWN
NABP1	1.387506	5.319192	6.708288	4.39E-06	0.000134	4.365729	UP
NCAM1	-2.05041	5.008279	-6.3931	7.86E-06	0.000188	3.800486	DOWN
NCAPG	1.691119	1.90037	3.663656	0.00202	0.008811	-1.35354	UP
NCF2	1.430519	4.625399	4.925734	0.000141	0.001271	0.957875	UP
NCKAP1L	1.75099	5.565004	8.314735	2.77E-07	2.82E-05	7.122824	UP
NDC80	1.427761	2.565602	5.213644	7.84E-05	0.00084	1.7134	UP

NDNF	-1.49877	3.195443	-5.60816	3.56E-05	0.000501	2.432059	DOWN
NDRG2	-1.54251	7.085411	-7.16487	1.93E-06	8.13E-05	5.135891	DOWN
NDUFA4L2	1.401445	4.780822	4.393828	0.000427	0.002835	-0.16126	UP
NEURL1B	1.754988	4.77093	8.471065	2.15E-07	2.44E-05	7.39256	UP
NFAM1	1.718941	3.663641	5.892255	2.04E-05	0.000346	2.96602	UP
NFIL3	-2.10588	4.679086	-7.98961	4.71E-07	3.73E-05	6.619225	DOWN
NOTCH3	1.494982	6.953732	6.092319	1.39E-05	0.000267	3.148599	UP
NPL	1.799843	4.4523	6.613242	5.23E-06	0.000149	4.259728	UP
NPR3	1.41954	4.052437	3.891267	0.001242	0.006134	-1.11618	UP
NR1D1	-2.68397	5.27282	-3.99993	0.000985	0.005194	-1.02003	DOWN
NR2F2	1.637472	5.769163	6.735321	4.18E-06	0.00013	4.396213	UP
NR4A1	-4.23442	7.255094	-10.7941	7.16E-09	5.41E-06	10.72795	DOWN
NR4A2	-3.66028	4.869282	-9.08738	8.23E-08	1.66E-05	8.330963	DOWN
NR4A3	-2.99752	3.244185	-8.95174	1.01E-07	1.74E-05	8.080374	DOWN
NRG2	-1.53853	2.300063	-6.1871	1.16E-05	0.000237	3.549703	DOWN
NRIP3	1.391639	2.433267	4.923579	0.000142	0.001276	1.149786	UP
NUDT10	-1.39903	2.543533	-6.44124	7.19E-06	0.000179	4.006261	DOWN
NUSAP1	1.373179	2.930119	3.916959	0.001176	0.005884	-0.93137	UP
NWD1	-1.56649	2.354673	-3.91433	0.001182	0.005903	-0.90174	DOWN
OCA2	-1.4349	1.786758	-5.87017	2.13E-05	0.000357	2.971691	DOWN
OPCML	-1.70135	2.149819	-7.24481	1.68E-06	7.56E-05	5.37673	DOWN
OSCAR	1.564515	1.991708	5.999523	1.66E-05	0.000299	3.20813	UP
P2RY13	2.073561	2.646471	8.04615	4.29E-07	3.59E-05	6.664234	UP
P2RY8	1.509036	1.744934	4.799949	0.000183	0.001523	0.935352	UP
PAMR1	-1.63772	7.091975	-7.82218	6.23E-07	4.37E-05	6.276259	DOWN
PAX7	-2.64559	3.491464	-8.28339	2.91E-07	2.85E-05	7.091996	DOWN
PCDH1	1.461299	4.341519	8.404518	2.39E-07	2.58E-05	7.291831	UP
PCDH11Y	-1.4447	2.333727	-3.1486	0.006057	0.020196	-2.4558	DOWN
PCLAF	1.747028	1.702357	5.258342	7.16E-05	0.000788	1.825866	UP
PCOLCE2	-1.4112	8.134148	-5.26915	7.01E-05	0.000774	1.519322	DOWN
PDE3B	-1.3706	5.072659	-8.01384	4.53E-07	3.64E-05	6.637884	DOWN
PDE4B	-1.66354	6.066135	-8.74145	1.40E-07	2.06E-05	7.781187	DOWN
PDE4D	-1.65594	4.638978	-7.33412	1.44E-06	6.93E-05	5.512102	DOWN
PDK4	-3.2007	7.277146	-11.5405	2.69E-09	3.93E-06	11.68582	DOWN
PDZRN4	-1.61424	4.875309	-5.24244	7.40E-05	0.000802	1.559049	DOWN
PEG10	-1.81483	3.352328	-9.1758	7.20E-08	1.49E-05	8.418318	DOWN
PER1	-2.31368	6.544546	-7.49853	1.08E-06	6.05E-05	5.733981	DOWN
PHYHIP	-1.71758	3.118144	-7.83671	6.08E-07	4.35E-05	6.378823	DOWN
PI16	-1.67507	4.307135	-5.82255	2.34E-05	0.000375	2.761232	DOWN
PIANP	-1.46127	2.228143	-4.4939	0.000346	0.002439	0.289808	DOWN
PIK3AP1	1.988558	4.266987	7.4676	1.14E-06	6.13E-05	5.771366	UP
PIK3CG	1.762148	2.379499	6.806587	3.67E-06	0.000121	4.639901	UP
PILRA	1.542616	3.78342	5.56429	3.88E-05	0.000525	2.323886	UP
PIR	-1.47245	4.653386	-7.71981	7.40E-07	4.86E-05	6.167035	DOWN

PIRT	-1.48081	1.930893	-4.98635	0.000125	0.001153	1.286893	DOWN
PKP2	-2.20984	4.551621	-6.5613	5.75E-06	0.000156	4.14727	DOWN
PLA2G7	1.84499	1.885053	5.367025	5.76E-05	0.000674	2.031208	UP
PLAU	1.569321	5.472191	8.4129	2.36E-07	2.58E-05	7.280587	UP
PLCH1	-2.93277	3.883041	-10.9437	5.86E-09	4.87E-06	10.79694	DOWN
PLEK	2.010617	5.285072	6.13484	1.28E-05	0.000254	3.31227	UP
PLEKHB1	-2.04096	4.307691	-7.12299	2.08E-06	8.34E-05	5.166184	DOWN
PLP1	-2.14512	3.510926	-7.04671	2.38E-06	9.19E-05	5.060953	DOWN
PLVAP	1.602029	5.581397	5.177604	8.44E-05	0.000878	1.388551	UP
PLXNB3	-1.37314	4.294553	-5.13247	9.25E-05	0.000943	1.389995	DOWN
PNPLA7	-1.61563	3.817374	-5.23547	7.50E-05	0.000813	1.650954	DOWN
PODXL2	-1.43083	3.395977	-6.84156	3.44E-06	0.000117	4.703865	DOWN
POSTN	1.604031	10.10039	6.229896	1.07E-05	0.000222	3.445842	UP
POU2AF1	1.393668	1.510609	2.931492	0.009576	0.028802	-2.78758	UP
POU2F2	2.139418	3.637633	8.684518	1.53E-07	2.12E-05	7.695791	UP
PPARGC1A	-1.5429	2.363788	-5.72609	2.82E-05	0.00043	2.695839	DOWN
PPP1R15A	-1.52844	6.899258	-8.33352	2.68E-07	2.75E-05	7.122645	DOWN
PPP1R16B	1.432958	3.321921	5.712032	2.90E-05	0.000438	2.638363	UP
PPP1R1B	-3.47895	4.953655	-6.97318	2.72E-06	9.96E-05	4.895532	DOWN
PPP1R9A	-1.85258	2.351068	-7.37849	1.33E-06	6.67E-05	5.606748	DOWN
PPP2R2B	-1.46707	2.260977	-5.65258	3.26E-05	0.000474	2.560081	DOWN
PRCD	-1.7371	3.133101	-5.43667	5.01E-05	0.000614	2.102806	DOWN
PRIMA1	-1.51315	3.220798	-4.74372	0.000206	0.001652	0.714228	DOWN
PRLR	-1.42438	3.407988	-4.98978	0.000124	0.001149	1.195287	DOWN
PRRT2	-1.4957	4.481363	-6.37685	8.11E-06	0.00019	3.79802	DOWN
PRRT4	-1.89732	3.060205	-5.80283	2.43E-05	0.000384	2.812805	DOWN
PRSS35	1.715184	3.167627	3.50268	0.002849	0.011354	-1.80497	UP
PTAFR	2.227742	4.179377	7.523759	1.03E-06	5.93E-05	5.869356	UP
PTGDS	-2.45916	8.653429	-5.69874	2.98E-05	0.000446	2.396909	DOWN
PTGS2	-1.84815	4.496547	-3.97658	0.001035	0.005395	-1.01473	DOWN
PTPN6	1.454384	4.667561	6.774239	3.89E-06	0.000125	4.529841	UP
PTPN7	1.397392	2.481483	5.000527	0.000121	0.001129	1.299309	UP
PTPRC	1.608931	5.734856	6.491088	6.55E-06	0.00017	3.945483	UP
PTPRO	1.420835	3.089409	6.016	1.61E-05	0.000294	3.223115	UP
PWP2	-1.67262	2.999139	-2.19962	0.042472	0.089787	-4.34982	DOWN
PWWP3B	-1.64513	2.538139	-2.66296	0.016739	0.044393	-3.43437	DOWN
PXDNL	-1.83569	3.572102	-7.13726	2.03E-06	8.29E-05	5.215851	DOWN
RAC2	1.659268	4.206445	7.347046	1.40E-06	6.86E-05	5.565177	UP
RASAL3	1.375883	2.574342	6.931844	2.93E-06	0.000104	4.860546	UP
RASD1	-1.58242	3.82554	-4.48977	0.000349	0.002449	0.131946	DOWN
RBKS	-1.86395	3.002689	-5.5808	3.76E-05	0.000517	2.390487	DOWN
RBM47	1.431663	4.056254	5.617587	3.49E-05	0.000495	2.401886	UP
RBP1	-1.37799	5.99066	-8.48432	2.11E-07	2.41E-05	7.374713	DOWN
RBP4	-1.68928	3.03966	-4.66506	0.000242	0.001865	0.570205	DOWN

RELN	-1.67628	2.577467	-4.78421	0.000189	0.001555	0.848302	DOWN
RGL3	-1.66268	4.485963	-5.63661	3.37E-05	0.000484	2.381961	DOWN
RGMA	-1.40454	4.643907	-5.25652	7.19E-05	0.000789	1.604917	DOWN
RGPD5	-1.413	5.009617	-7.33515	1.43E-06	6.93E-05	5.491659	DOWN
RGS1	-2.00332	4.107937	-7.27678	1.59E-06	7.30E-05	5.441357	DOWN
RGS11	-1.56066	4.986267	-7.01223	2.53E-06	9.52E-05	4.925462	DOWN
RGS4	1.508283	4.931313	4.196773	0.000648	0.003844	-0.58675	UP
RHOB	-1.94368	8.550018	-11.9111	1.69E-09	3.38E-06	12.14056	DOWN
RIC3	-1.75953	3.48471	-8.03221	4.39E-07	3.61E-05	6.699225	DOWN
RNASE6	1.44517	3.927985	6.593514	5.42E-06	0.000151	4.24876	UP
RND2	-1.78987	3.588423	-8.86665	1.16E-07	1.92E-05	7.982774	DOWN
RNF122	-1.65672	5.119389	-10.423	1.19E-08	7.01E-06	10.22888	DOWN
RPS6KA1	1.386409	3.784474	7.075327	2.26E-06	8.86E-05	5.108108	UP
RRM2	1.930603	2.728249	4.000862	0.000983	0.005193	-0.73356	UP
RSPO3	1.415305	2.181474	5.099419	9.89E-05	0.000986	1.506835	UP
RTP4	1.498535	1.859553	5.938969	1.87E-05	0.000325	3.096964	UP
RUBCNL	1.637648	2.989197	6.469762	6.81E-06	0.000172	4.055579	UP
S100A1	-1.51428	2.477653	-4.14747	0.00072	0.004162	-0.43542	DOWN
S100A8	3.46147	2.408615	5.041543	0.000111	0.001066	1.388239	UP
S100A9	3.390408	4.731379	6.876506	3.23E-06	0.000112	4.752545	UP
S100B	-1.69396	6.473275	-8.63645	1.66E-07	2.12E-05	7.611433	DOWN
SALL3	-1.96001	5.477652	-11.4851	2.89E-09	3.93E-06	11.60474	DOWN
SAMD9	1.453423	3.691726	7.0236	2.48E-06	9.41E-05	5.021063	UP
SASH3	1.792141	3.677075	8.852738	1.18E-07	1.94E-05	7.951385	UP
SCARA5	-2.12073	6.351324	-8.38514	2.47E-07	2.62E-05	7.215557	DOWN
SCG2	1.693125	5.621166	5.434616	5.03E-05	0.000615	1.907434	UP
SCIMP	1.579955	2.625334	5.467019	4.71E-05	0.00059	2.202001	UP
SCN7A	-1.82956	3.244919	-7.31024	1.50E-06	7.07E-05	5.514278	DOWN
SCX	-1.54397	3.081222	-5.42287	5.15E-05	0.000623	2.079332	DOWN
SDC1	1.88487	3.045194	3.965208	0.001061	0.005472	-0.83742	UP
SEMA3B	-1.67753	6.713982	-6.61072	5.25E-06	0.000149	4.132572	DOWN
SEMA5B	1.369849	1.774711	3.838961	0.001389	0.006637	-0.99281	UP
SEMA6B	1.432094	2.82968	4.371459	0.000448	0.00294	0.010971	UP
SERPINA1	2.415076	3.829855	8.1904	3.39E-07	3.02E-05	6.946067	UP
SERPINA3	-1.59643	6.610529	-2.51184	0.0228	0.055926	-4.17281	DOWN
SEZ6L2	1.895144	2.341926	4.928842	0.00014	0.001269	1.167201	UP
SFRP1	-2.15691	8.036173	-9.19528	6.99E-08	1.49E-05	8.472131	DOWN
SGCA	-1.37178	3.935585	-3.56277	0.002506	0.010344	-1.81559	DOWN
SGSM1	-1.42165	3.891229	-9.02542	9.05E-08	1.70E-05	8.231878	DOWN
SIGLEC1	1.397281	5.438993	5.319139	6.34E-05	0.000716	1.679795	UP
SIGLEC10	1.858042	2.16964	5.905663	1.99E-05	0.000339	3.036881	UP
SIGLEC14	1.797943	2.20094	4.871739	0.000158	0.001374	1.060667	UP
SIGLEC9	1.728662	2.30725	5.838985	2.26E-05	0.000369	2.912789	UP
SIK1B	-2.30758	4.194132	-8.79716	1.29E-07	1.97E-05	7.894817	DOWN

SIRPB2	1.425408	3.134975	4.719065	0.000216	0.00172	0.691834	UP
SLA	1.644967	4.663036	6.192772	1.15E-05	0.000235	3.459846	UP
SLAMF6	1.672404	1.783306	6.053658	1.50E-05	0.000282	3.303587	UP
SLAMF8	1.50789	4.344953	6.326112	8.92E-06	0.000199	3.731503	UP
SLC11A1	1.690688	4.074574	3.644253	0.002105	0.009097	-1.62311	UP
SLC14A1	-1.52053	4.50313	-4.09394	0.000807	0.004508	-0.77821	DOWN
SLC16A12	-1.95014	3.835884	-7.19106	1.84E-06	7.94E-05	5.302509	DOWN
SLC16A3	1.370439	5.216911	5.623904	3.45E-05	0.000493	2.305161	UP
SLC16A9	-1.76222	3.957157	-6.78698	3.80E-06	0.000124	4.586018	DOWN
SLC19A2	-1.96194	4.237444	-10.3582	1.30E-08	7.01E-06	10.10504	DOWN
SLC22A5	-1.41217	4.615925	-8.73252	1.42E-07	2.07E-05	7.795911	DOWN
SLC2A5	2.050115	2.552879	6.386126	7.97E-06	0.000189	3.907714	UP
SLC37A2	1.38229	4.681742	4.546702	0.00031	0.002242	0.167966	UP
SLC38A5	1.546425	1.731432	4.274769	0.00055	0.003424	-0.10919	UP
SLC6A1	-1.56302	4.611422	-5.74774	2.71E-05	0.000416	2.586387	DOWN
SLC7A5	-1.43068	4.198772	-7.03454	2.43E-06	9.34E-05	5.009928	DOWN
SLCO2B1	1.662945	6.957552	8.530323	1.96E-07	2.27E-05	7.440218	UP
SLCO4A1	-1.48003	3.10438	-3.61803	0.002227	0.009466	-1.59177	DOWN
SLITRK5	-1.51728	1.954881	-6.70531	4.41E-06	0.000135	4.460551	DOWN
SMTNL2	-1.85674	2.733163	-6.69328	4.51E-06	0.000136	4.452273	DOWN
SNX10	1.691008	2.332794	6.246874	1.04E-05	0.000218	3.65744	UP
SNX20	1.854955	2.571357	8.200975	3.33E-07	3.02E-05	6.895525	UP
SOCS2	-1.54565	5.030554	-6.45849	6.96E-06	0.000175	3.911537	DOWN
SORCS1	-2.82924	2.872529	-8.26318	3.01E-07	2.85E-05	7.034057	DOWN
SOX8	-1.67546	4.85068	-6.14207	1.26E-05	0.000252	3.330082	DOWN
SPN	1.465042	2.930621	5.35615	5.88E-05	0.000683	1.97257	UP
SPOCD1	1.554441	1.744712	4.141772	0.000729	0.004195	-0.37755	UP
SPOCK3	-2.67493	5.162122	-7.90756	5.40E-07	4.04E-05	6.474559	DOWN
SPP1	4.640863	8.111314	9.514263	4.34E-08	1.18E-05	8.953816	UP
SRCIN1	-1.83157	1.938353	-6.45396	7.02E-06	0.000176	4.023546	DOWN
ST14	2.169697	2.551223	5.094883	9.98E-05	0.000992	1.48537	UP
ST8SIA4	1.729059	3.647225	7.370844	1.35E-06	6.74E-05	5.616174	UP
STEAP1	1.643791	3.749133	4.703989	0.000223	0.001763	0.610052	UP
STK32A	-1.50214	4.198495	-7.54813	9.92E-07	5.78E-05	5.896996	DOWN
STMN2	3.215699	3.46528	6.656896	4.82E-06	0.000142	4.387399	UP
STXBP2	1.41865	3.739204	5.752381	2.68E-05	0.000413	2.689233	UP
STXBP5L	-1.50502	2.196913	-6.24378	1.04E-05	0.000218	3.652326	DOWN
SUGCT	1.406611	3.314925	5.127863	9.33E-05	0.000947	1.497985	UP
SYBU	-1.80483	4.621941	-7.49468	1.09E-06	6.05E-05	5.790817	DOWN
SYK	1.885803	4.974337	8.702909	1.49E-07	2.08E-05	7.750698	UP
SYT12	1.773218	3.79897	4.59458	0.000281	0.00208	0.384248	UP
TAC1	-1.76191	1.87382	-3.67102	0.001988	0.008712	-1.3565	DOWN
TBX20	-1.88644	6.227502	-9.0061	9.32E-08	1.70E-05	8.189242	DOWN
TBXAS1	1.37733	4.178209	4.816526	0.000177	0.001486	0.784405	UP

TC2N	-1.73167	4.798547	-9.18089	7.14E-08	1.49E-05	8.472354	DOWN
TCEAL2	-1.97036	4.98206	-6.86836	3.28E-06	0.000113	4.674113	DOWN
TCEAL5	-1.70707	2.832382	-8.74823	1.39E-07	2.06E-05	7.758532	DOWN
TCEAL6	-2.28033	1.941636	-6.29037	9.54E-06	0.000207	3.734287	DOWN
TENM3	-1.92249	3.66392	-9.33856	5.63E-08	1.36E-05	8.670071	DOWN
TENM4	1.42803	3.329433	4.111028	0.000778	0.004394	-0.57156	UP
TF	-1.62651	4.874439	-5.50038	4.41E-05	0.000569	2.076666	DOWN
TFEC	1.793439	3.037076	6.59996	5.35E-06	0.00015	4.287383	UP
TFPI2	-1.46887	6.167663	-5.53835	4.09E-05	0.000542	2.072689	DOWN
THBS2	1.731529	9.534159	10.27667	1.45E-08	7.01E-06	10.03255	UP
THBS4	-2.38012	5.431978	-6.22341	1.08E-05	0.000225	3.461058	DOWN
THY1	1.450755	6.407085	5.855955	2.19E-05	0.000361	2.700025	UP
TIMD4	2.197061	2.140579	5.161088	8.72E-05	0.000904	1.630652	UP
TIMP3	-1.52645	12.24058	-6.96602	2.75E-06	0.0001	4.853293	DOWN
TIMP4	-2.08954	4.200763	-6.62719	5.09E-06	0.000148	4.287877	DOWN
TIPARP	-2.07476	5.624249	-14.7058	7.03E-11	1.01E-06	15.137	DOWN
TLCD4	-1.85072	4.575426	-10.3275	1.35E-08	7.01E-06	10.08463	DOWN
TLR7	1.913237	3.047237	8.157548	3.57E-07	3.11E-05	6.866084	UP
TLR8	2.046611	1.890234	7.268327	1.61E-06	7.30E-05	5.381844	UP
TM4SF18	2.326561	1.955472	8.558567	1.87E-07	2.22E-05	7.349499	UP
TM7SF2	-1.79211	3.481924	-4.7817	0.00019	0.001559	0.768794	DOWN
TMEM100	-1.67794	4.961153	-7.17635	1.89E-06	8.00E-05	5.219558	DOWN
TMEM119	1.369457	4.984738	4.398805	0.000423	0.002815	-0.1747	UP
TMEM132C	-2.50513	5.183659	-7.88279	5.63E-07	4.19E-05	6.430243	DOWN
TMEM38A	-1.42247	2.891896	-6.40157	7.74E-06	0.000186	3.930248	DOWN
TMEM86A	1.490756	3.519447	6.33672	8.74E-06	0.000197	3.800374	UP
TMIGD3	1.429776	3.365141	6.055788	1.49E-05	0.000281	3.28647	UP
TNC	1.660782	9.069368	8.236091	3.14E-07	2.92E-05	6.972938	UP
TNFAIP8L2	1.69326	2.571649	8.075216	4.09E-07	3.47E-05	6.706006	UP
TNFRSF11A	1.613017	2.741935	6.580006	5.56E-06	0.000153	4.252663	UP
TNFRSF11B	1.461878	6.701553	6.103564	1.36E-05	0.000265	3.174237	UP
TNFSF10	1.622168	5.216096	8.264774	3.00E-07	2.85E-05	7.051867	UP
TNFSF8	1.397019	2.316957	5.341006	6.06E-05	0.000698	1.970247	UP
TOP2A	2.188819	3.681685	4.56836	0.000296	0.002167	0.350611	UP
TPX2	1.711114	2.996473	4.369274	0.00045	0.002948	-0.0053	UP
TRAF3IP3	1.778002	2.599768	5.952506	1.82E-05	0.000319	3.119192	UP
TRDN	-1.39375	2.43518	-3.01372	0.008055	0.025256	-2.73607	DOWN
TREM1	2.003622	3.092945	4.315579	0.000504	0.003205	-0.11989	UP
TREM2	1.938083	3.459823	4.662567	0.000243	0.001872	0.556753	UP
TRHDE	-2.25095	3.502175	-8.55511	1.88E-07	2.22E-05	7.510858	DOWN
TRIM14	1.452355	4.141521	6.736697	4.17E-06	0.00013	4.495497	UP
TSPAN11	1.431326	3.8773	4.181261	0.00067	0.003946	-0.48871	UP
TSPAN7	-1.40975	5.088202	-6.76696	3.94E-06	0.000126	4.474751	DOWN
TSPYL2	-1.53504	6.676941	-7.58735	9.27E-07	5.50E-05	5.879077	DOWN

TTC30B	1.407434	2.323931	5.363143	5.80E-05	0.000677	2.012644	UP
TTYH1	-1.66329	1.799209	-6.99485	2.61E-06	9.70E-05	4.94463	DOWN
TUBA1A	1.375347	8.20071	10.96006	5.73E-09	4.87E-06	10.94766	UP
TUBA1C	1.768471	5.660622	7.412128	1.25E-06	6.40E-05	5.611225	UP
TUBB2B	-1.88558	5.100407	-9.05184	8.69E-08	1.67E-05	8.27661	DOWN
TUBB3	1.452815	3.524604	3.859746	0.001329	0.006429	-1.1123	UP
UBASH3B	1.714026	2.814472	4.619521	0.000266	0.002002	0.518425	UP
ULBP2	1.379661	1.56229	4.570207	0.000295	0.002163	0.487197	UP
USP2	-1.42746	2.113255	-5.97316	1.75E-05	0.00031	3.160149	DOWN
VAMP8	1.562803	5.517176	7.36093	1.37E-06	6.79E-05	5.524695	UP
VASH2	-1.59254	2.275932	-8.49963	2.06E-07	2.37E-05	7.33638	DOWN
VAT1L	-1.87178	5.54873	-11.1263	4.60E-09	4.45E-06	11.15583	DOWN
VAV1	1.528362	3.913502	7.322183	1.47E-06	6.98E-05	5.529961	UP
VCAM1	1.698035	6.544681	7.483502	1.11E-06	6.07E-05	5.706405	UP
VIPR1	-1.47475	3.978738	-3.98452	0.001018	0.005328	-0.94022	DOWN
VIT	-2.25634	2.875451	-5.1554	8.82E-05	0.00091	1.567179	DOWN
VNN1	1.571801	1.949924	4.695094	0.000227	0.001791	0.720879	UP
VSIG4	1.684626	6.606043	7.216161	1.77E-06	7.83E-05	5.23665	UP
VTN	-2.06091	3.773267	-4.7734	0.000193	0.00158	0.727844	DOWN
VWF	1.819329	6.798266	5.60567	3.58E-05	0.000503	2.201075	UP
WIF1	-2.96232	2.933206	-6.53289	6.06E-06	0.000163	4.16645	DOWN
WSCD1	1.460356	1.90044	5.429471	5.08E-05	0.000619	2.148945	UP
ZBTB16	-2.24105	4.839992	-5.08297	0.000102	0.00101	1.255813	DOWN
ZBTB9	1.369827	2.769443	6.635337	5.02E-06	0.000147	4.350305	UP
ZDHHC11	-1.51373	2.735244	-4.927	0.000141	0.001269	1.123371	DOWN
ZDHHC11B	-2.09053	3.909816	-9.62824	3.67E-08	1.10E-05	9.091187	DOWN
ZFP36	-2.46303	7.256545	-13.081	4.17E-10	1.56E-06	13.49737	DOWN
ZNF281	1.385513	3.876544	8.386319	2.47E-07	2.62E-05	7.259278	UP
ZNF331	-1.77744	5.451908	-13.7166	2.03E-10	1.01E-06	14.14004	DOWN
ZNF365	-1.45827	4.019766	-7.01683	2.51E-06	9.49E-05	4.988424	DOWN
ZNF469	1.656005	3.687704	6.700795	4.45E-06	0.000135	4.453985	UP
ZNF483	-1.40105	2.195549	-6.78442	3.82E-06	0.000124	4.603554	DOWN
ZNF92	1.473298	2.844134	7.126243	2.07E-06	8.34E-05	5.196676	UP
ZWINT	1.439946	2.758207	5.059095	0.000107	0.00104	1.399486	UP

LncRNA	Log FC	AveExpr	t	P.Value	adj.P.Val	B	change
CEROX1	-1.54771	4.958301	-6.30371	9.30E-06	0.000205	3.626287	DOWN
DIO3OS	-2.07608	4.266497	-4.86873	0.000159	0.00138	0.872442	DOWN
DNM3OS	1.684592	5.095338	7.547342	9.93E-07	5.78E-05	5.868559	UP
DUXAP8	1.634614	3.485149	4.91078	0.000146	0.001298	1.053006	UP
FAM27E3	-1.40413	1.99169	-6.62774	5.09E-06	0.000148	4.327641	DOWN
FZD10-AS1	-1.42586	3.863405	-7.59661	9.13E-07	5.46E-05	5.988264	DOWN
GATA6-AS1	-1.78382	3.558726	-7.24292	1.68E-06	7.56E-05	5.397369	DOWN
H19	2.156464	5.227455	4.242874	0.000588	0.003583	-0.4944	UP
HAND2-AS1	-1.67143	5.904902	-6.57921	5.56E-06	0.000153	4.092968	DOWN

HAS2-AS1	-1.45303	3.323009	-6.30696	9.24E-06	0.000204	3.743629	DOWN
ITGB2-AS1	1.480516	1.681058	5.542842	4.05E-05	0.000538	2.364522	UP
KLF3-AS1	-1.75663	3.528047	-10.5463	1.00E-08	6.51E-06	10.28059	DOWN
LENG8-AS1	-1.45778	2.425306	-7.41319	1.25E-06	6.40E-05	5.665939	DOWN
LINC00092	-1.81884	2.002229	-6.00602	1.64E-05	0.000296	3.22074	DOWN
LINC00511	-1.51139	3.197133	-6.95484	2.81E-06	0.000101	4.905972	DOWN
LINC00702	1.600155	2.75741	3.500995	0.00286	0.011383	-1.76438	UP
LINC01094	1.547178	2.586279	6.153876	1.24E-05	0.000248	3.488908	UP
LINC01896	-1.8669	3.148829	-8.97754	9.74E-08	1.71E-05	8.118262	DOWN
LINC-PINT	-2.11723	4.551032	-9.74651	3.09E-08	1.03E-05	9.285224	DOWN
MALAT1	-1.52796	11.86995	-7.4442	1.19E-06	6.29E-05	5.686891	DOWN
NEAT1	-1.41456	10.76572	-5.31097	6.44E-05	0.000724	1.663525	DOWN
NR4A1AS	-4.05175	3.872615	-7.19853	1.82E-06	7.94E-05	5.323442	DOWN
SLC9A3-AS1	-1.46638	2.835237	-4.27158	0.000553	0.00344	-0.2145	DOWN
TARID	-1.444	2.593475	-6.33222	8.81E-06	0.000199	3.809984	DOWN
THY1-AS1	1.51984	3.435512	4.547055	0.00031	0.002241	0.316621	UP
TRHDE-AS1	-2.2852	2.90199	-8.27621	2.94E-07	2.85E-05	7.057728	DOWN

Supplementary Table 5 Immune cell infiltration in GSE55492

immune cell	GSM1338 133	GSM1338 134	GSM1338 135	GSM1338 136	GSM1338 137	GSM1338 138	GSM1338 139	GSM1338 140	GSM1338 141	GSM1338 142
B cells naive	0.005313	0.016373	0	0	0.007105	0	0.043332	0	0.004835	0
B cells memory	0.003695	0	0.001182	0.005842	0	0.028688	0	0.003841	0	0.011008
Plasma cells	0.005801	0.002641	0.010891	0.000503	0	0.017657	0.043429	0.015705	0.020233	0
T cells CD8	0.050095	0.092317	0.046326	0.097286	0.019355	0.076257	0.077628	0.103914	0.155333	0.0831
T cells CD4 naive	0	0	0	0	0	0	0	0	0	0
T cells CD4 memory resting	0.049601	0.109929	0.062943	0.066435	0.041205	0.018305	0.004106	0.038508	0	0.081151
T cells CD4 memory activated	0	0	0.001261	0	0	0.00311	0	0.007679	0.007366	0
T cells follicular helper	0	0.006265	0	0	0.001532	0.007877	0.010196	0	0	0
T cells regulatory (Tregs)	0.012263	0.021611	0.015401	0.026705	0.005381	0.010984	0.063452	0.008518	0.02526	0.000819
T cells gamma delta	0	0	0	0	0	0	0	0	0	0
NK cells resting	0	0	0.002889	0	0.031322	0	0	0.010377	0	0
NK cells activated	0.067595	0.041898	0	0.022261	0	0.019862	0.048734	0	0	0.015131
Monocytes	0.001964	0.036637	0	0	0.072457	0	0.016032	0	0.008498	0.022541
Macrophages M0	0	0	0	0	0	0	0.050133	0	0	0
Macrophages M1	0.046019	0.045835	0.008998	0.006672	0.048516	0.029425	0.024803	0.017293	0.022368	0.016888
Macrophages M2	0.67884	0.556174	0.792776	0.714439	0.676106	0.728762	0.528647	0.690588	0.644657	0.664506
Dendritic cells resting	0.005981	0.037296	0	0	0.002607	0	0.012401	0	0.004926	0
Dendritic cells activated	0	0	0	0	0	0	0	0	0	0
Mast cells resting	0.069065	0.033025	0.057332	0.055683	0.091566	0.050446	0.058269	0.103577	0.104872	0.102674
Mast cells activated	0	0	0	0	0	0.006517	0	0	0	0
Eosinophils	0	0	0	0	0	0	0.009273	0	0	0
Neutrophils	0.003769	0	0	0.004174	0.002848	0.00211	0.009565	0	0.001651	0.002182

immune cells	GSM1338124	GSM1338125	GSM1338126	GSM1338127	GSM1338128	GSM1338129	GSM1338130	GSM1338131	GSM1338132
B cells naive	0.018047	0.038419	0.018357	0.039098	0.013233	0.000694	0.034959	0.046214	0.050518
B cells memory	0	0	0	0	0.019016	0	0	0.002632	0.005431
Plasma cells	0.010972	0.018684	0.005993	0.012868	0.00635	0.000203	0.021154	0	0.050391
T cells CD8	0.079033	0.12228	0.100475	0.110157	0.119727	0.138738	0.189858	0.135433	0.045937
T cells CD4 naive	0	0	0	0	0	0	0	0	0
T cells CD4 memory resting	0	0.08683	0.089083	0.053018	0	0.098589	0.082022	0.061796	0
T cells CD4 memory activated	0	0.009067	0.008165	0.008635	0	0.002334	0.001032	0.003376	0
T cells follicular helper	0.016067	0	0	0	0.007723	0.006215	0	0	0.016417
T cells regulatory (Tregs)	0.03836	0.0412	0.027866	0.036988	0.044458	0.042452	0.015868	0.07703	0.027877
T cells gamma delta	0.029427	0	0	0	0	0	0	0	0.026304
NK cells resting	0	0.000893	0	0	0	0	0.011889	0	0
NK cells activated	0.078499	0.007532	0.030804	0.00306	0.04726	0.004283	0.033932	0.096938	0.009099
Monocytes	0	0.063396	0.124727	0.019586	0	0.025469	0.042687	0.022563	0
Macrophages M0	0.221233	0.040023	0	0	0.109662	0	0	0.010258	0.251376
Macrophages M1	0.031895	0.016344	0.029663	0.034591	0.023004	0.050184	0.010518	0.022323	0.020389
Macrophages M2	0.400261	0.489213	0.522152	0.59281	0.512037	0.564162	0.48536	0.388813	0.445751
Dendritic cells resting	0.006629	0	0	0.003481	0.008702	0.000932	0	0.006572	0.009357
Dendritic cells activated	0	0	0	0	0	0	0	0	0
Mast cells resting	0.069578	0.060216	0.042542	0.083212	0.082311	0.063434	0.06963	0.11934	0.026719
Mast cells activated	0	0	0	0	0	0	0	0	0
Eosinophils	0	0.002462	0	0	0	0	0	0	0
Neutrophils	0	0.003441	0.000173	0.002495	0.006516	0.00231	0.001091	0.006711	0.014433

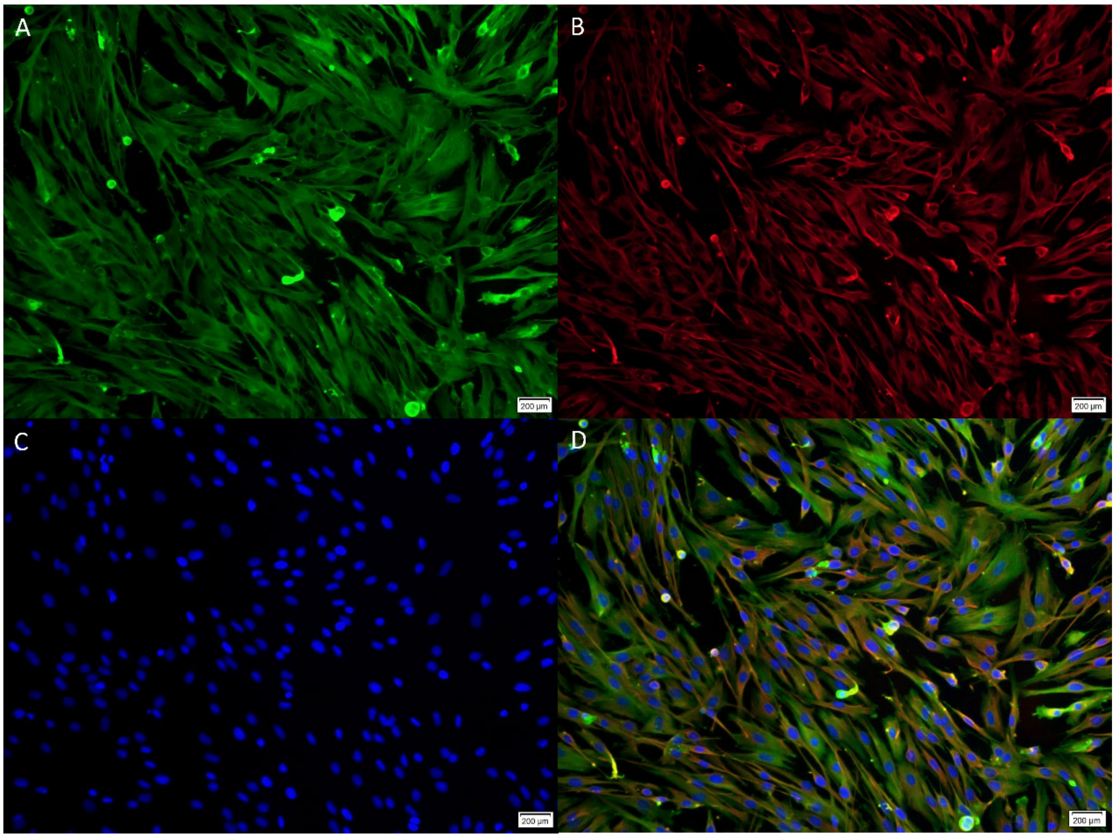

Supplementary Figure 1 The immunofluorescence of VICs. (A) a-SMA (positive rate almost 100%), (B) Vimentin (positive rate 70-80%), (C) DAPI, (D) Merge result.

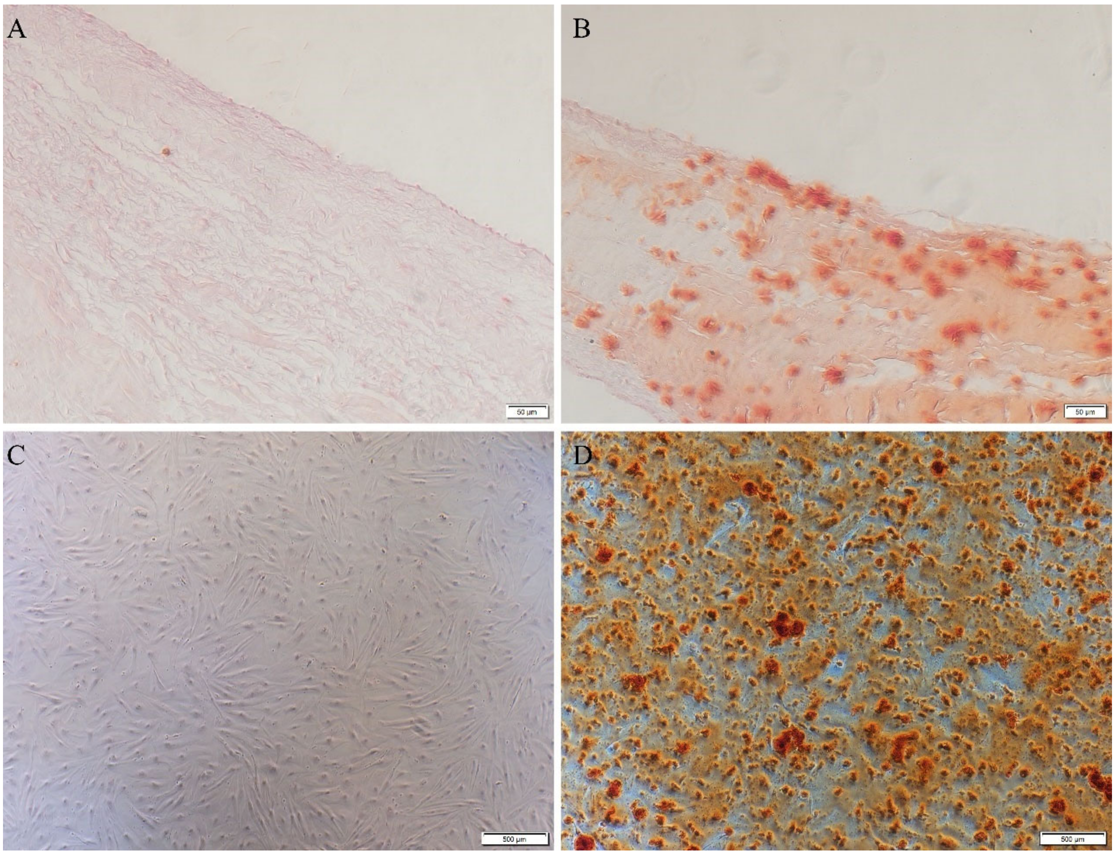

Supplementary Figure 2 Alizarin red staining for valve samples and VICs. (A) Aortiv valve in the control group with no obvious calcium nodules, (B) Positive alizarin red staining results in calcified aortic valves, (C) VICs in normal medium, (D) VICs in osteogenic medium on day 7.

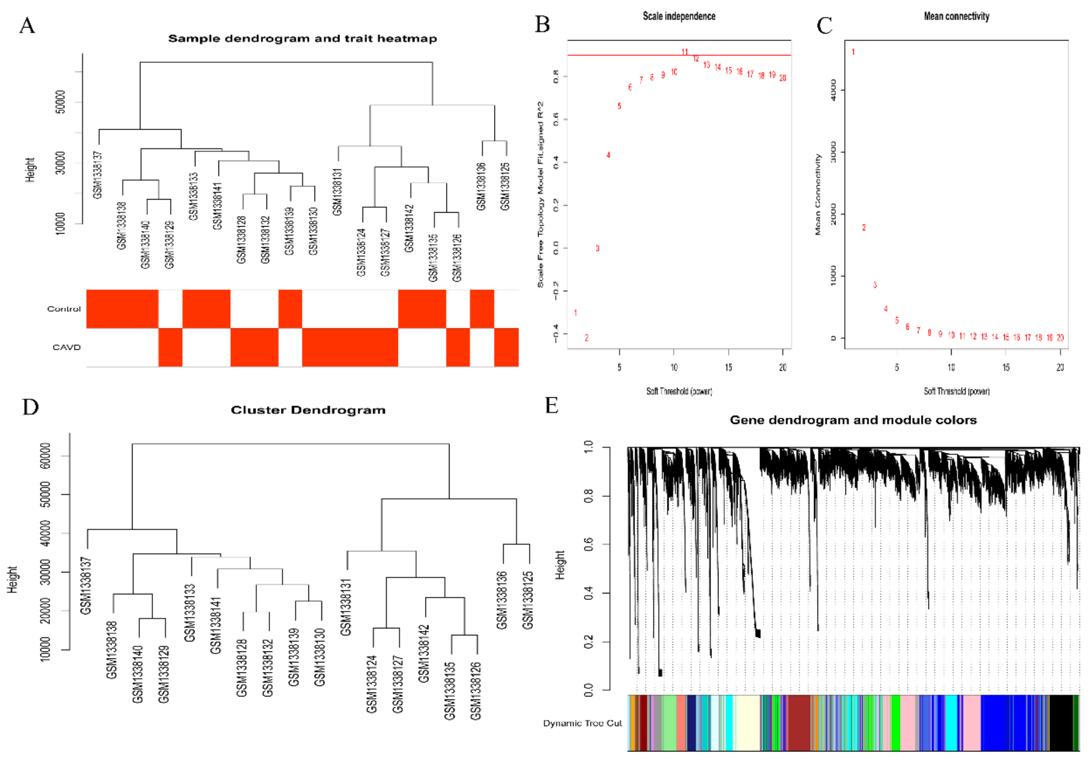

Supplementary Figure 3 The results of WGCNA in GSE55492 (A) Sample dendrogram and trait heatmap, (B) soft-thresholding power selection, (C) Cluster Dendrogram, (D) Gene dendrogram and module colors

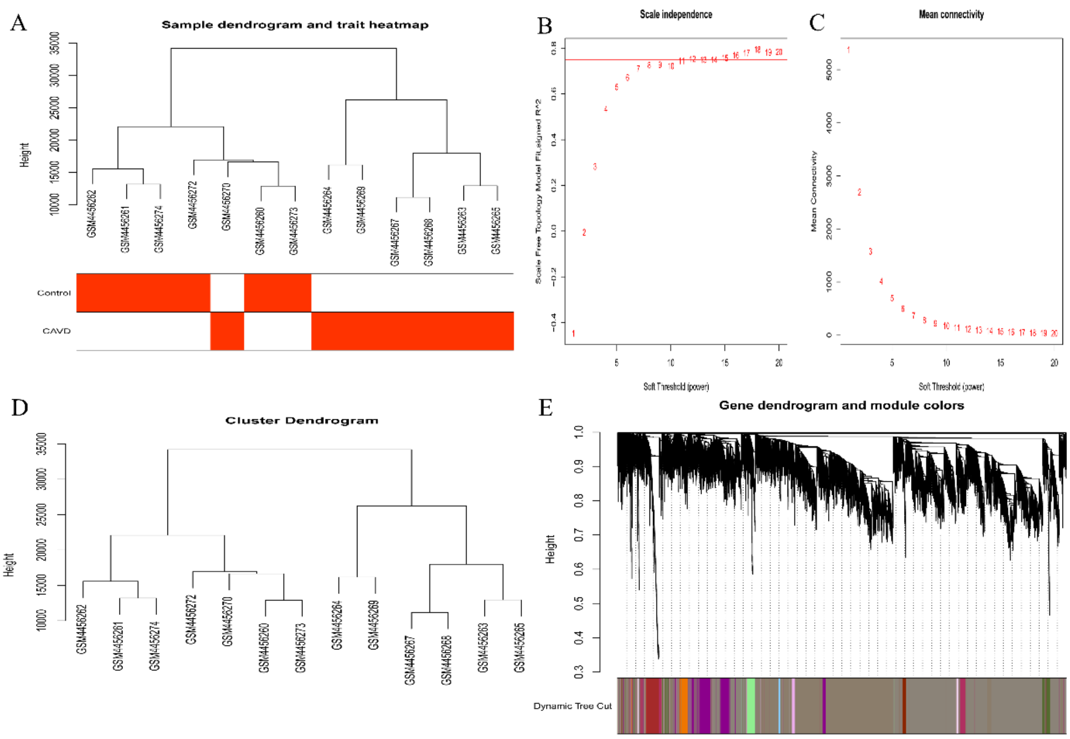

Supplementary Figure 4 The results of WGCNA in GSE148219 (A) Sample dendrogram and trait heatmap, (B) soft-thresholding power selection, (C) Cluster Dendrogram, (D) Gene dendrogram and module colors
